# Genomic, transcriptomic, and proteomic insights into the symbiosis of deep-sea tubeworm holobionts

**DOI:** 10.1038/s41396-019-0520-y

**Published:** 2019-10-08

**Authors:** Yi Yang, Jin Sun, Yanan Sun, Yick Hang Kwan, Wai Chuen Wong, Yanjie Zhang, Ting Xu, Dong Feng, Yu Zhang, Jian-Wen Qiu, Pei-Yuan Qian

**Affiliations:** 1 Department of Ocean Science, Division of Life Science and Hong Kong Branch of The Southern Science and Engineering Guangdong Laboratory, The Hong Kong University of Science and Technology, Hong Kong, China; 2 Department of Biology, Hong Kong Baptist University, Hong Kong, China; 3 CAS Key Laboratory of Ocean and Marginal Sea Geology, South China Sea Institute of Oceanology, Chinese Academy of Sciences, 510301 Guangzhou, China; 4 Laboratory for Marine Geology, Qingdao National Laboratory for Marine Science and Technology, 266061 Qingdao, China; 5 College of Life Sciences and Oceanography, Shenzhen University, Shenzhen, China

**Keywords:** Comparative genomics, Transcriptomics, Proteomics

## Abstract

Deep-sea hydrothermal vents and methane seeps are often densely populated by animals that host chemosynthetic symbiotic bacteria, but the molecular mechanisms of such host-symbiont relationship remain largely unclear. We characterized the symbiont genome of the seep-living siboglinid *Paraescarpia echinospica* and compared seven siboglinid-symbiont genomes. Our comparative analyses indicate that seep-living siboglinid endosymbionts have more virulence traits for establishing infections and modulating host-bacterium interaction than the vent-dwelling species, and have a high potential to resist environmental hazards. Metatranscriptome and metaproteome analyses of the *Paraescarpia* holobiont reveal that the symbiont is highly versatile in its energy use and efficient in carbon fixation. There is close cooperation within the holobiont in production and supply of nutrients, and the symbiont may be able to obtain nutrients from host cells using virulence factors. Moreover, the symbiont is speculated to have evolved strategies to mediate host protective immunity, resulting in weak expression of host innate immunity genes in the trophosome. Overall, our results reveal the interdependence of the tubeworm holobiont through mutual nutrient supply, a pathogen-type regulatory mechanism, and host-symbiont cooperation in energy utilization and nutrient production, which is a key adaptation allowing the tubeworm to thrive in deep-sea chemosynthetic environments.

## Introduction

Siboglinid tubeworms are often conspicuous members of the benthic communities of deep-sea hydrothermal vents and cold seeps [1, 2]. They are mouthless and gutless yet can have high productivity [3]. Symbiosis with γ-proteobacteria, a group of chemosynthetic bacteria, is a key adaptation allowing tubeworms to thrive in vent and seep ecosystems [4]. Larvae of the tubeworms obtain free-living γ-proteobacteria from the ambient environment through a symbiont-specific infection process [5, 6]. In the adult stage, the symbiotic bacteria are housed in a specialized organ of their host called the trophosome and no longer in direct contact with the ambient environment [5]. Substrates for chemosynthesis including sulfide and oxygen are obtained from ambient seawater through the branchial plume of the host or from the sediment through the posterior end of the host and delivered to the symbiont through the host’s circulation system which uses hemoglobin [7, 8].

The genomes of both the host and the symbiont contain critical genetic information about the symbiosis. Among the 194 species in 34 genera of Siboglinidae, none has a published genome, although the genomes of several species are being sequenced by multiple groups. Only eight endosymbiont genomes (from *Escarpia spicata*, *Lamellibrachia luymesi*, *Galathealinum brachiosum*, *Ridgeia piscesae*, *Riftia pachyptila*, *Seepiophila jonesi*, *Tevnia jerichonana* and *Osedax frankpressi*) have been sequenced [9–13]. Previous studies of the symbiosis in siboglinids have primarily focused on the giant tubeworm *R. pachyptila*, which revealed that its symbiont uses both the Calvin–Benson cycle and the rTCA cycle for carbon fixation; meanwhile, the symbiont has a complete pathway of heterotrophic metabolism, and thus can live mixotrophically [7, 14]. Siboglinid symbionts vary in their capability of using the Calvin-Benson and rTCA cycles [9, 10], indicating a greater number of comparative genomic analyses is required to fully understand the mechanisms by which tubeworms adapt to different environmental conditions and thrive in vent and seep habitats. A comparative analysis of the metaproteomics of two vent-dwelling tubeworms (i.e., *R. pachyptila* and *T. jerichonana*) living in quite different geofluid environments at the host’s plume level shows highly consistent protein expression profiles in sulfur metabolism, carbon fixation and oxidative stress [11]. Although previous studies found that the symbionts in the trophosome have evolved a pathogen-type defense mechanism to protect themselves from the host, symbiont virulence intensity and its regulation of host immunity for symbiosis maintenance have not been studied; many studies have investigated siboglinid symbiosis only from the perspective of either the host or symbiont [9, 14, 15]. Given that different siboglinid tubeworms live in various chemosynthesis habitats from hydrothermal vents and cold seeps to sunken wood, it is necessary to analyze the host and symbiont as a holobiont in order to reveal the adaptive mechanisms of tubeworm symbiosis.

In the present study, we sequenced the endosymbiont genome, metatranscriptome, and metaproteome of the siboglinid tubeworm *Paraescarpia echinospica* (the holobiont) inhabiting cold seeps in the South China Sea of the West Pacific Ocean [16, 17]. As the first integrated genomic, transcriptomic, and proteomic analysis of cold-seep tubeworm, the present study aimed to decipher the interdependence between the host and symbiont with particular emphases on how the symbiont uses various metabolic pathways to generate energy, how the host and symbiont cooperate in nutrient provisions, and how the two partners regulate each other. Furthermore, a comparative analysis was conducted with other published vestimentiferan symbiont genomes to reveal genetic basis of adaption to the vent- and seep-environments [10].

## Materials and methods

### Sampling *Paraescarpia echinospica* and metagenomic sequencing

*P echinospica* individuals were collected from a cold-seep area situated on the northern continental slope of the South China Sea at a water depth of 1147 m (22.11619° N, 119.2856° E). Sampling was conducted using the remotely operated vehicle (ROV) ROPOS onboard the R/V Tan Kah Kee on 19 April 2018 (see Supplementary Fig. S1 showing the tubeworms prior to sampling and a complete individual preserved in 95% ethanol). The tubeworms were placed into an insulated “Biobox” with a closed lid to minimize changes in temperature in the water inside the container. It took ~40 min for the ROV to ascend from the seabed to the main deck of the research vessel. Once the worms were brought onboard the research vessel, they were dissected, with their trophosome (an organ that harbors symbionts), plume (a gill-like organ) and vestimentum (primarily made up of muscle) fixed separately in RNA*later*® (Invitrogen, USA), and then stored at −80 °C. Total DNA of the trophosome was extracted using the DNeasy Blood & Tissue Kit (Qiagen, Halden, Germany) according to the manufacturer’s protocol. The symbiont genome was sequenced with both the Oxford Nanopore Technology and Illumina platforms and assembled. Briefly, an 8–10 kb Nanopore DNA library was constructed using the Ligation Sequencing Kit 1D (Oxford Nanopore, Oxford, UK) according to the manufacturer’s protocol and sequenced with the FLO-MIN106 R9.4 flow cell coupled to the MinION^TM^ platform (Oxford Nanopore Technologies, Oxford, UK) at the Hong Kong University of Science and Technology. The raw reads were base-called according to the protocol in the MinKNOW and written into. *fast5* files. Illumina DNA sequencing was performed using the Illumina HiSeq™ X-Ten to produce 150 bp paired-end reads at the Beijing Genomics Institute (BGI) in Shenzhen.

### Symbiont genome assembly and functional annotation

Trimmomatic v0.33 [18] was used to trim the Illumina adapters and low-quality bases (base quality ≤ 20). The clean reads were assembled using SPAdes v3.9.1 [19] with *k*-mer sizes of 21, 33, 55, 77, 99, and 127 bp, and the products were pooled. Genome binning was then conducted as in previous studies [20, 21]. Briefly, the clean reads were first mapped to the assembled contigs using Bowtie2 v2.2.9 [22], and the coverage of each contig was calculated using SAMTOOLS v1.3.1. Open reading frames (ORFs) were then predicted using Prodigal v2.6.3 [23] and protein functional domains were predicted using HMMER 3.1b2 [24] under the 100 + HMM model. Taxonomic affiliation of all HMM positive ORFs were determined using BLASTp [25] against NCBI nonredundant (NR) protein database, and the taxonomic assignment of each protein was imported to MEGAN v5.7.0 [26] using the lowest common ancestor (LCA) method with the parameters of Min Score 50, Max Expected 0.01, Top Percent 5 and LCA Percent 100. The results were analyzed in RStudio (https://www.rstudio.com/) with the libraries of vegan, plyr, RColorBrewer and alphahull. Sequences representing the draft symbiont genome were then extracted from the assembled contigs of both the host and the symbiont, based on the combination of sequencing coverage and GC content (Supplementary Fig. S2a) [21]. Contigs belonging to the potential bacterial genome were further determined using principal component analysis (PCA) of tetranucleotide frequencies (Supplementary Fig. S2b), assessed using CheckM v1.0.6 [27], and further scaffolded using SSPACE-LongRead v1.1 [28] by adding Nanopore long reads. The newly assembled scaffolds were binned again using the above-mentioned pipeline. GapFiller v1.10 [29] was then used to fill the gaps in the binned symbiont genome, and the completeness and potential contamination of the binned genome were estimated using CheckM v1.0.6 [27]. Coding sequences (CDS) in the *P. echinospica* symbiont genome were predicted and translated using Prodigal v2.6.3 [30]. The translated protein sequences were functionally annotated with RPS-BLAST v2.2.15 (*e*-value < 10^–05^) against the databases of Clusters of Orthologous Groups (COGs) for prokaryotes, Gene Ontology (GO) and Pfam using WebMGA online analysis [31]. Sequences were annotated with KEGG (Kyoto Encyclopedia of Genes and Genomes) numbers against the KEGG database using BLASTp, and KEGG Mapper was run on the KAAS to construct the metabolic pathways of the symbiont from these sequences [32].

Raw sequencing data of the *P. echinospica* metagenome have been deposited in NCBI’s Sequence Read Archive database under BioProject PRJNA472657 and BioSample SAMN09239911. The 16S rRNA gene sequence of the *P. echinospica* symbiont has been deposited in GenBank under the accession number MH628048. The complete genome sequences of the symbiont have been deposited in DDBJ/ENA/GenBank under the accession number RZUD00000000.

### Genomic comparison and phylogenomic analysis of siboglinid symbionts

**Fig. 1:**
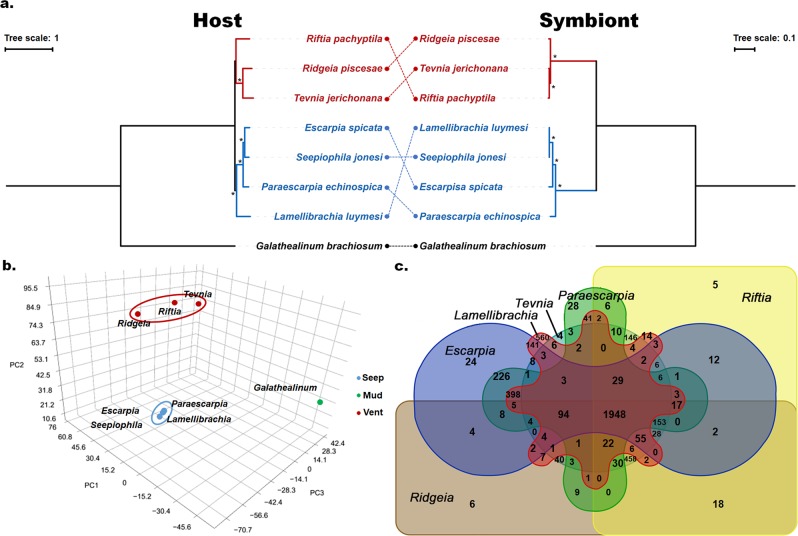
**a** Cophylogeny analysis of bacterial symbionts (right side) and their associated siboglinid hosts (left side). Vent- and seep-living vestimentiferans are colored in red and blue respectively. All nodes have 100% bootstrap support. **b** PCA analysis on the orthologous proteins in 1305 OGs of the endosymbionts of Siboglinidae under the BLOSUM62 model. **c** Venn diagram depicting unique and shared orthologous gene clusters in each of the six endosymbiont genomes

Four seep-living and three vent-dwelling vestimentiferans endosymbiont genome sequences (Table 1) were compared using BLASTn 2.2.26 [33], then visualized using BRIG [34] and Circoletto [35] to provide an overview of genome sequence similarity (Supplementary Fig. S3). In particular, the orthologous groups (OGs) from the above seven endosymbiont genomes and a mud-dwelling siboglinid endosymbiont genome were detected using Proteinortho v5.16b [36] (BLAST threshold *E* = 1 × 10^−10^). Only single-copy genes in each OG that were found in all taxa were retained for phylogenomic analysis, resulting in 1305 OGs. Sequences of each OG were aligned using MUSCLE and trimmed using TrimAL v1.4 [37]. After concatenating these alignments, a phylogenetic tree was constructed using RaxML version 8.2.4 [38] under the GTR + Γ model with the partition information of each orthologous gene and 1000 bootstrap replicates. Similarly, a phylogenetic analysis of the siboglinids based on the 13 concatenated mitochondrial genes [39] was performed using RaxML version 8.2.4 [38] under the GTR + CAT model (Fig. 1a). PCA analysis on the orthologous proteins in 1305 OGs was performed using Jalview [40] under the BLOSUM62 model, the similarity scores between each pair of sequences were calculated to form the matrix, the components were generated and then visualized using BioVinci (Bioturing, San Diego, CA, USA) (Fig. 1b). A Venn diagram was constructed using Venn webtool (http://bioinformatics.psb.ugent.be/webtools/Venn/) to illustrate the shared and unique orthologous genes among the seep- and vent-dwelling vestimentiferan endosymbionts (Fig. 1c). Based on the results from Proteinortho, orthologous genes that were only present in symbionts from a particular habitat were classified as unique genes to that habitat, e.g. vent-unique or seep-unique genes. In addition, a hidden Markov model (HMM)-based approach delta-bitscore (DBS) [41, 42] was used to identify the functional divergence of shared orthologous proteins in seep- and vent-dwelling vestimentiferan endosymbiont genomes, their adaptability to the host and habitat was mined through these loss-of-function mutations.

**Table 1:** The general genomic features of vestimentiferan endosymbionts

Symbiont	Accession No.	Genome size (Mb)	No. of contigs	*N*_50_ (kb)	GC%	No. of CDS	No. of functions assigned	Host
***Paraescarpia*** **symbiont**	RZUD00000000	4.06	14	381.7	54.8	3 525	2 906	Seep-living vestimentifernas
***Escarpia*** **symbiont**	QFXE00000000	4.06	23	313.6	54.2	–	–	
***Lamellibrachia*** **symbiont**	QFXD00000000	3.53	337	20.6	54.3	–	–	
***Seepiophila*** **symbiont**	QFXF00000000	3.53	323	20.6	54.3	–	–	
***Ridgeia*** **symbiont**	LDXT00000000	3.44	97	84.0	58.9	3 158	2 699	Vent-dwelling vestimentiferans
***Riftia*** **symbiont**	AFOC00000000	3.48	197	29.7	58.8	3 488	2 890	
***Tevnia*** **symbiont**	AFZB00000000	3.64	184	92.7	58.2	3 367	2 827	

Genome data—symbiont of *Paraescarpia*: this study; symbionts of *Esarpia*, *Lamellibrachia* and *Seepiophila*: Li et al. [10]; symbiont of *Ridgeia*: Perez and Juniper [12]; symbionts of *Riftia* and *Tevnia*: Gardebrecht et al. [11]

### Tubeworm holobiont transcriptome sequencing

The same *P*. *echinospica* individual used for metagenome sequencing was also subjected to transcriptome sequencing. Total RNA of the plume, that of the vestimentum and that of the trophosome were extracted using Trizol (Invitrogen, USA) following the manufacturer’s protocol. A cDNA library of each body region was then constructed and sequenced on the HiSeq™ 4000 platform (Illumina, San Diego, CA) at BGI in Shenzhen to produce 100 bp paired-end reads. Since the trophosomal RNA includes the sequences from both the host and the symbiont, another library of the trophosome was constructed after removing the prokaryotic RNA so as to sequence the remaining eukaryotic RNA [43]. Therefore, two sets of sequencing data were produced for the trophosome: one including the transcripts of both the host and the symbiont, and the other including only host transcripts.

### De novo holobiont transcriptome assembly and sequence analysis

Adapters and low-quality reads (base quality ≤ 20) were trimmed with Trimmomatic v0.33 [18]. Clean reads from the plume, vestimentum, and trophosome (including prokaryotic reads) were pooled and assembled using Trinity version 2.1.0 [44] under default settings. Only the highest expressed isoforms were retained. CD-HIT-EST [45] was used to further reduce redundant sequences with a threshold of 90% similarity. TransRate [46] was used to detect errors such as chimeric artifacts, incomplete assembly and base errors in the assembled holobiont transcriptome. TransDecoder [47] was used to detect coding regions while BLAST-2.2.31+ [25] was applied to search holobiont proteins against NCBI NR protein database. Taxonomical assignment of the annotated transcripts was then performed using the LCA assignment algorithm in MEGAN v5.2.3 [26] with the top 10 hits of each transcript in the NR database, which allowed the sorting of both the host and symbiont transcripts. The transcriptomes of the host and symbionts were produced and separated. BUSCO v3 was used to evaluate the comprehensiveness of the *P*. *echinospica* transcriptome assembly [48]. Blast2GO v4.0.7 [49] was applied to assign GO terms to the transcripts. Transcript expression levels in each region (plume, vestimentum, and trophosome) were quantified and expressed in transcripts per million (TPM) using Salmon [50]. To understand the region-specific gene functions, the transcripts of each region with at least ten parts per million were retained [51] and blasted against the databases of Eukaryotic Orthologous Groups of proteins (KOG), COGs of proteins for prokaryotes and KEGG using RPS-BLAST v2.2.15 [31] (*e*-value < 10^−05^). The resultant KEGG Orthology assignments were mapped to KEGG pathways with KEGG Mapper on the KEGG Automatic Annotation Server v2.0 (KAAS) [32]. A gene was considered to be specifically expressed in a particular region if its TPM value in the region accounted for more than 75% of the total TPM of all three regions [51]. Besides, transcriptome data of *R. pachyptila*, *R. piscesae*, *E. spicata*, *L. luymesi*, *S. jonesi* and *G. brachiosum* were obtained from the NCBI SRA database for phylogenomic analyses (Supplementary Table S1, see Supplementary Information Methods for details).

Raw sequence data of the three regions have been deposited in NCBI Sequence Read Archive database under BioProject PRJNA494962 and SAMN09239911. Holobiont metatranscriptome sequences have been deposited in DDBJ/EMBL/GenBank under the accession number GHDL00000000. *P echinospica* transcriptome sequences (without prokaryotes) have been deposited in DDBJ/EMBL/GenBank under the accession number GHDM00000000.

### Metaproteomic analysis

Three individuals of *P. echinospica* were used to determine the protein expression pattern in the trophosome. Specific details of protein extraction, SDS-PAGE, in-gel trypsin digestion, LC–MS/MS, protein identification and quantitation can be found in Supplementary Information Methods. In brief, tissues (~0.1 g of wet weight) were collected from the trophosome region of three *P. echinospica* individuals, which served as three replicates. Proteins were extracted, purified, quantified, separated using SDS-PAGE and in-gel digested with trypsin. Resulting peptides were separated and analyzed on a liquid chromatography system coupled with mass spectrometry. The host and symbiont proteins were identified and quantified using Mascot version 2.3.0, and all converted mass spectrometry.*mgf* data were searched against the translated protein databases of *P. echinospica* and its endosymbionts. Protein abundance was represented as an emPAI value, and the 70 most abundant proteins in the trophosome and its endosymbiont were chosen to visualize protein expression. The mass spectrometry metaproteomic dataset has been deposited in the ProteomeXchange Consortium via PRIDE [52] with the accession number PXD013944.

### Real-time PCR validation

Real-time PCR was employed to validate the expression patterns of selected genes in the trophosome, as well as the plume and vestimentum for comparison. The primers of each gene were designed using the on-line NCBI Primer-BLAST tool (Supplementary Table S2, see Supplementary Information Methods for details). Total RNA was extracted from each region from three *Paraescarpia* individuals using the Trizol method. Residual contaminant DNA was removed using the TURBO DNA-free kit (Thermo Fisher Scientific). The first strand cDNA was then synthesized using the High Capacity cDNA Reverse Transcription Kit (Applied Biosystems). Real-time PCR was performed with the SYBR^®^ Green RT-PCR Reagents Kit (Applied Biosystems) on LightCycler 480 II (Roche) (see Supplementary Information Methods for procedural details). All samples and negative controls were amplified in triplicate. Triplicates were applied for each gene, and the relative gene expression level was calculated based on the 2^ΔΔCt^ method [53]. The standard deviation (SD) was calculated and Student’s *t* tests were performed with Microsoft Excel.

## Results and discussion

### The symbiont genome and comparative genomics

An examination of the 16S rRNA microbial community data revealed a single bacterial ribotype in the trophosome of *P. echinospica* with its phylogenetic position shown in Supplementary Fig. S4. Sequencing the trophosomal genomic DNA using the Illumina platform produced 235,260,418 paired-end reads. After assembling the reads, binning was conducted on contigs over 500 bp, and the results showed that the sequences of *P. echinospica* and its symbiont were well separated by sequencing coverage and GC content (Supplementary Fig. S2). There was only one 16S rRNA gene sequence among the potential symbiont contigs, which was identical to that obtained from the 16S rRNA gene clone library sequence, further confirming that *P. echinospica* potentially harbored a single genotype of bacterial endosymbiont. Meanwhile, sequencing the trophosomal genomic DNA using the Nanopore MinION platform produced 1,158,101 reads, with an average length of 2.1 kb and an N50 statistic of 3.3 kb. A draft genome of the *P. echinospica* symbiont, assembled using both the Illumina and Nanopore reads, was 4.06 Mb in total length with 14 scaffolds. The maximum scaffold length was 942.6 kb, and the N50 length was 381.7 kb (Table 1). CHECKM analysis showed that this genome was 97.4% of completeness with 2.6% contamination and encoded 3525 predicted CDS. Among those CDS, 2906 (82.4%) had at least one significant hit in the COG, KEGG, Pfam and GO databases (Supplementary Table S3). Both the percentage and the number of genes in different GO and COG categories are shown in Supplementary Fig. S5. The general genomic features of the endosymbiont of *Paraescarpia* and its close relatives based on 16S rRNA gene analysis (Supplementary Fig. S4) are shown in Table 1, which indicates that the assembled *P. echinospica* symbiont is of high quality.

Our phylogenomic analysis of siboglinid holobionts showed that siboglinids and their endosymbionts did not co-speciate, while the endosymbionts were well clustered into two clades by vent and seep habitats (Fig. 1a, Supplementary Fig. S6). PCA analysis also showed that the endosymbionts of Siboglinidae were clustered strictly according to habitat type, with the seep- and vent-vestimentiferans being well separated (Fig. 1b). Gene orthology analysis showed that 1430 OGs and 677 OGs were unique to seep- and vent-dwelling vestimentiferan endosymbionts, respectively. Together, these results indicate independent evolutionary history of endosymbiosis between seep- and vent-dwelling vestimentiferans.

**Fig. 2:**
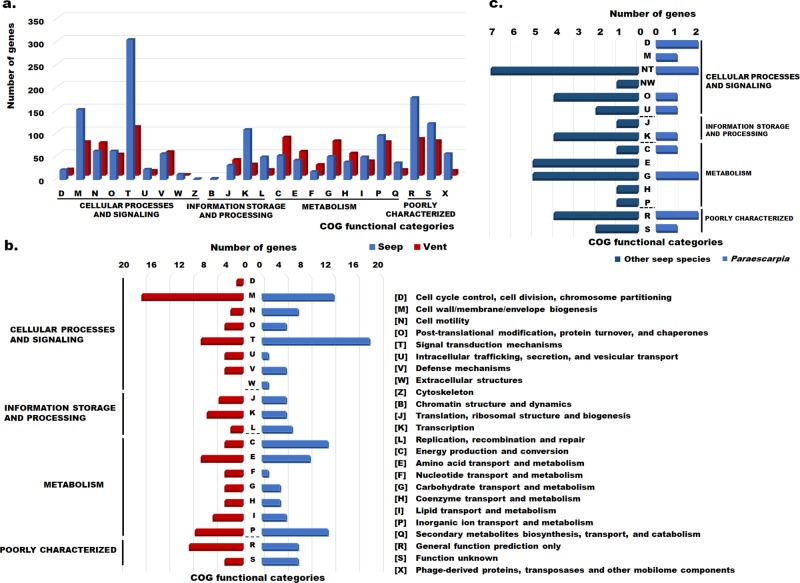
**a** Number of seep-unique and vent-unique orthologous genes in different COG categories. **b** Number of vent and seep loss-of-function genes in different COG categories. Red and blue colors represent genes belonging to vent- and seep-living symbionts respectively. **c** Number of seep loss-of-function genes in different COG categories. Light and dark blue colors represent genes belonging to the *Paraescarpia* symbiont and other seep-living symbionts respectively

To understand the genetic basis of such habitat-specific endosymbiosis, we analyzed the functional composition of the seep- and vent-unique genes. The seep-unique genes contributed more than vent-unique genes to cell wall/membrane/envelope biogenesis [M], signal transduction [T] and mobile genetic elements [X] (Fig. 2a). The [M] category contains a lipid II flippase MurJ (*murJ*) which transports lipid in cell wall formation [54], a mechanosensitive channel of small conductance (*mscS*) [55] and an outer membrane efflux protein TolC (*tolC*) [56, 57], which control the efflux of solutes and solvent from the outer membrane. Due to their potential for material transportation and osmosis regulation, these proteins are considered to be critical for bacterial survival, including antimicrobial resistance, symbiosis, and adaptation to adverse environments [54–57]. Their presence in seep-dwelling vestimentiferans may mediate adaptation of the symbionts to changes in their osmotic environment. The [T] category contains signal transduction histidine kinases (*baeS*, *ntrY*), which are known to sense and transmit environmental stimuli to response regulator containing CheY receiver, GGDEF domain, DNA-binding domains (*citB*, *atoSC*, *ompR*) which may activate symbiont responses to environmental signals and then the chemotaxis protein CheC (*cheC*) may enable symbionts to move toward more favorable environments [58]. Genes in [T] category are critical for bacterial chemotactic adaptation.

In natural habitats, vent-vestimentiferans grow at the basaltic base of vent chimneys and exclusively use their plumes for substances acquisition from the seawater, while seep-vestimentiferans grow in soft sediments and bury their posterior ends at greater depths in the sediments for absorbing substances [7, 59, 60] (Supplementary Fig. S1b). The endosymbionts of seep- and vent-vestimentiferans likely come from the free-living population in sediments and seawater, respectively [61, 62]. Vestimentiferans have the potential to adopt symbionts that optimally adapt to the local environment [60, 63]. In comparison to vent fluids, seep sediments often contain high concentrations of dissolve inorganic nutrient (e.g. nitrates, nitrites, ammonium, and phosphorus) [64–66] and harbor higher microbial community diversity and richness [67–70]. Therefore, the complex environment of the seep sediment may have driven the molecular adaptation in the endosymbiont of seep-dwelling vestimentiferans. Consequently, it led to a large number of seep-unique genes in the [X] category in the endosymbionts of seep-vestimentiferans, indicating these endosymbionts have the capacity to acquire more foreign genetic elements than the endosymbionts of vent-vestimentiferans. Gene acquisition is important in the adaptive evolution of prokaryotes as the acquisition of mobile genetic elements (e.g. genes encoding integrase, transposase, and phage-related proteins) may change the virulence potential of symbionts and confer their resistance to toxic compounds and other virulence factors in seep sediments [71, 72]. In addition, seep-unique genes encoding phage or phage-derived proteins have the potential to aid the symbionts in host immune evasion [73], and virulence genes that participate in bacterial capsules (e.g. capsule polysaccharide export proteins KpsS, KpsC, KpsE) may enable the symbionts to defend against phagocytosis as well as other aspects of the host immune system [72]. These results show that the seep-living siboglinid endosymbionts are more prone than the vent-dwelling siboglinid endosymbionts to resist environmental stress and use pathogen-like mechanisms to evade host immune responses to survive intracellularly. A list of seep- and vent-unique genes is included in Supplementary Excel Table S4.

To show the effects of variation in shared orthologous clusters in symbionts, we used a profile HMM-based method DBS to capture functional genetic changes in conserved domains within shared orthologous protein sequences [41, 42]. Figure 2b shows the number of genes, classified by functions, with loss-of-function mutations in the endosymbionts of seep-living and vent-dwelling vestimentiferans. Notably, a CRISPR-associated protein Cse1 (*cse1*) in the endosymbionts of seep-living vestimentiferans, a bacterial defense protein against foreign genetic elements [74], has lost its function, which may have caused a larger number of foreign genetic elements in seep-unique genes than in vent-unique genes (Fig. 2a). Furthermore, 28 genes were unique to the endosymbiont of *Paraescarpia* (Fig. 1c) and another 15 genes lost their functions (Fig. 2c). In the *Paraescarpia* symbiont-unique genes, nitroreductase has the potential to degrade or transform toxic nitro-containing compounds from the environment [75], which provides an advantage for *Paraescarpia* symbionts adapting to seep sediments with highly total nitrogen concentrations [64, 65]. Many symbionts (e.g., siboglinid symbionts, rhizobia, enteropathogenic *Escherichia coli*) use a type II (T2SS) or a type III secretion system to evade phagocytosis and facilitate infection [10]. Instead, genes encoding type VI secretion system (T6SS) proteins (*vasD*, *impJKL*) and the hemolysin activation/secretion protein were found in the list of the *Paraescarpia* symbiont-unique genes. T6SS and the hemolysin activation/secretion protein play an important role as a transporter or pore in transporting proteins within a bacterial cell or between cells across the cell envelope. Furthermore, as an important virulence factor in Gram-negative bacteria allowing them to defend against competing organisms, T6SS mediates only bacterial intercellular interactions during symbiosis establishment rather than host cells [76, 77].

We concluded that the adaptation of vestimentiferan holobionts to vents and seeps with different physical-chemical and biotic factors is largely an attribute to differences in their symbiont genetic components and that the endosymbionts of seep-living vestimentiferans have more advantages than the endosymbionts of vent-dwelling vestimentiferans in terms of both environmental adaptation (free-living stage) and host-bacterium interaction (symbiotic stage). Among the seep-vestimentiferan endosymbionts, one with *Paraescarpia* has a higher potential than those with other species to reduce the toxicity of organic nitrogen compounds from the environment and transport proteins (including virulence factors) between cells for provision of intermediate product and nutrients. The loss-of-function orthologous genes from vent- and seep-living species are listed in Supplementary Excel Table S5.

### The holobiont metatranscriptome and metaproteome

**Fig. 3:**
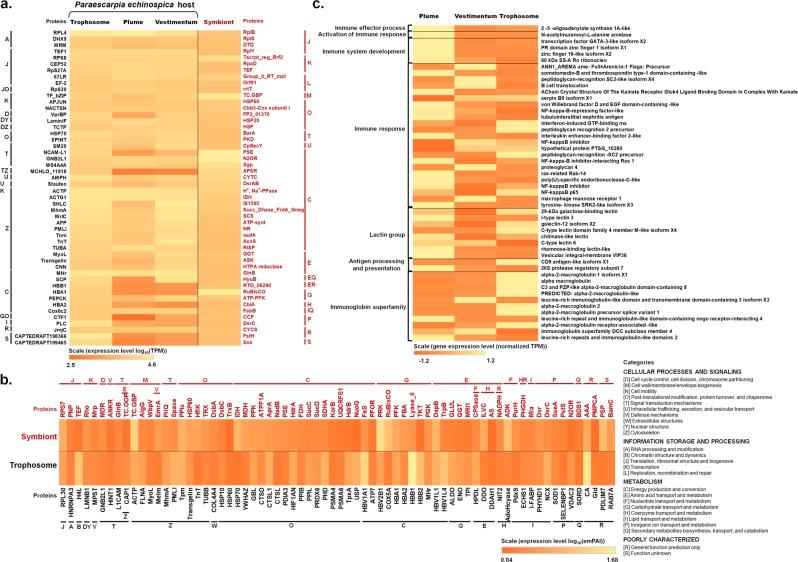
Heat map of **a** the 50 most highly expressed genes of *P. echinospica* and those of its symbiont as identified in the metatranscriptome analysis, **b** the 70 most abundant proteins of the trophosome and those of its symbiont as identified in the metaproteome analysis, and **c** the top 50 most highly expressed immune-related genes in the plume, vestimentum and trophosome. Each grid represents an identified gene/protein in the respective sample. The color represents the gene expression level (based on Log-transformed and normalized TPM/emPAI values of the selected genes/proteins). Protein abbreviations annotated from the host and the symbiont are listed on the two sides (see the list of abbreviations for the full names of proteins in Supplementary Information and Supplementary Tables S7 for details). Based on KOG and COG annotation, proteins are classified as shown in the lower right of the graph. Functionally redundant genes/proteins and genes/proteins of unknown function are excluded from this figure. The complete dataset is shown in Supplementary Excel Table S8

RNA-Seq of the plume, the vestimentum and the two sets of trophosome (the holobiont and only the host) produced 39,111,919, 40,807,720, 36,212,117 and 23,460,651 paired-end reads, respectively. After trimming, 37,098,488, 38,670,009 and 34,158,322 clean and high-quality reads from the plume, vestimentum, and trophosome (holobiont reads) were assembled to produce the holobiont transcriptome. Similarly, after trimming, 21,380,499 reads from the trophosome (almost without prokaryotes) were assembled with the reads of the plume and vestimentum to produce the *P. echinospica* transcriptome. In the holobiont transcriptome, over 90.5% of the 1112 bacterial transcripts were specific to the trophosome [5]. Among the 142,750 transcripts retained, 23,810 coding regions were predicted by TransDecoder while functional annotation matched 1087 translated proteins of the symbiont, each of which had at least one significant hit in the NCBI NR, COG, KEGG or GO databases. On the other hand, for the *P. echinospica* transcriptome, among the 118,820 transcripts retained, 22,284 coding regions were predicted by TransDecoder. Functional annotation matched 20,733 translated proteins, each of which had at least one significant hit in the NCBI NR, KOG, KEGG, or GO databases (Supplementary Table S6). BUSCO analysis shows that the *P. echinospica* transcriptome is 97.7% of completeness assessed with 978 metazoan BUSCOs, which compares favorably with the completeness of transcriptome assemblies of several other species of vestimentiferans [78]. The 50 most highly expressed genes in the *P. echinospica* and their respective expression levels in the plume, vestimentum and trophosome, as well as the 50 most highly expressed genes in the symbiont are shown in Fig. 3a.

To find the protein evidence in the holobiont, total proteins extracted from the trophosome region of another three *Paraescarpia* individuals were identified and quantified by LC-MS/MS, resulted in 1767 host proteins and 474 endosymbiont proteins (Supplementary Information Methods). The 70 most abundant proteins of the trophosome and symbionts are shown in the heat map along with their relative abundances (Fig. 3b). Correlation of proteomic data and RNA profiling is shown in Supplementary Fig. S7.

### Host-microbe interdependence

#### Energy sources

**Fig. 4:**
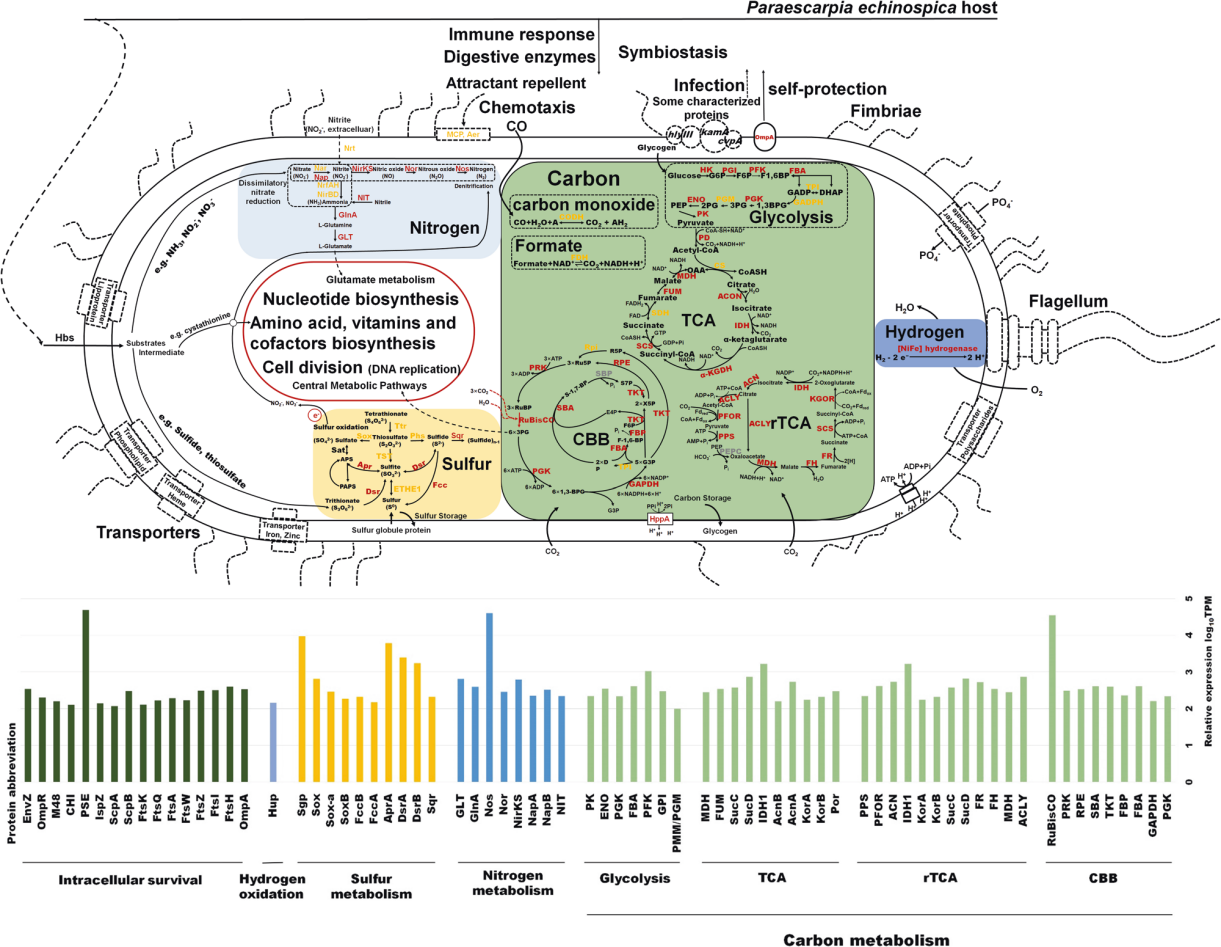
An overview of metabolic pathways of the *P. echinospica* endosymbiont. Different metabolic pathways are presented in squares of different colors. Nitrogen metabolism is in a light blue square, including dissimilatory nitrate reduction, denitrification, and ammonia assimilation. Carbon metabolism is in a green square, including CBB and rTCA cycles for carbon fixation, TCA and glycolysis cycles for organic carbon utilization and bidirectional reactions of carbon monoxide and formate. Sulfur metabolism is in a yellow square. The sulfur oxidation depends on the Dsr, Apr and Sox systems. The sulfur globule protein is highly expressed and acts as an energy storage compound. The hydrogen oxidation is in a blue violet square. The above energy-conversion pathways provide substrates and energy for the production of nutrients such as amino acids and vitamins (Table 2). Enzymes found in both the symbiont genome and transcriptome are shown in red, whereas those found in the symbiont genome only are shown in yellow, and the missing enzymes are shown in gray. The histogram at the bottom shows the relative gene expression levels (log_10_TPM) of enzymes in different metabolic pathways and key proteins involved in intracellular survival mechanisms. The membrane transport proteins, bacterial chemotaxis proteins and some of the characterized proteins for bacterial infection, which were encoded in the symbiont genome but not expressed, are marked with dashed circles. The flagellum, fimbriae and pilus of the symbiont, which were encoded in the symbiont genome but not expressed, are indicated in dashed line. The full names of enzymes are given in the list of abbreviations in Supplementary Information, and the involved genes are listed in Supplementary Table S9

Similar to other vestimentiferans endosymbionts in previous study [10], the *P. echinospica* symbiont genome included the genes responsible for all of the essential metabolic pathways for energy production and conversion in free-living chemoautotrophic sulfur-oxidizing bacteria (Fig. 4). Our results indicate that the *P. echinospica* symbiont was highly versatile in its energy use, with the ability to use thiosulfate, carbon monoxide (CO), and hydrogen as alternative energy sources. Interestingly, anaerobic oxidation of CO has not been reported in the endosymbiont of siboglinids before but has been found in the symbiont of the gutless marine worm *Olavius algarvensis* [79]. The identification of anaerobic carbon monoxide dehydrogenase (CODH) gene (*cdhA*) in *P. echinospica* symbiont genome indicates CO can be a potential energy source for tubeworm symbionts. This ability is likely a key adaption allowing *P. echinospica* to thrive in more reducing habitats. However, unlike the abundant expression of anaerobic CODHs in the *O.* *algarvensis* symbiont (high CO concentrations (17–51 nM) in its habitat), anaerobic CODHs were not found in the transcriptome or proteome of the *P. echinospica* symbiont. We speculate that this might be due to the low content of CO in its habitat (currently no data on CO concentrations). The transportation of host-supplied substrates in *P. echinospica* holobiont is given in Supplementary Information (see the section on substrates supply and energy conversion, Supplementary Figs. S8–S11 and Supplementary Tables S9, S10).

#### Carbon fixation

Two carbon fixation pathways (i.e., the Calvin–Benson–Bassham (CBB) cycle and the reductive tricarboxylic acid (rTCA) cycle) have been reported in the endosymbionts of the siboglinids [9, 10]. The CBB and rTCA cycles also coexisted in the symbiont of *P. echinospica* (Fig. 4). In the CO_2_ fixing process by CBB cycle, pyrophosphate-dependent phosphofructokinases (*PPi-PFK*) were co-encoded with proton-translocating pyrophosphatase (*hppA*) in the symbiont of *P. echinospica* and their co-transcription was confirmed in our transcriptome analysis which allows the symbiont to consume less energy at least 9.25% [80, 81]. Furthermore, the rTCA cycle is more energetically efficient than the CBB cycle [80, 82], and the rTCA cycle genes (*korB*, *por*, *sdhA*) were highly expressed in the *P. echinospica* symbiont, suggesting an active rTCA cycle in this symbiont (Fig. 3b and Fig. 4). These observations indicated that such metabolic strategy with a low energy demand could give the *P. echinospica* an advantage when living under energy- and nutrient-poor environmental conditions.

#### Holobiont nutrition

The symbiont of *P. echinospica* possessesed the typical metabolic pathways of nutrient generation, including the biosynthesis of carbohydrates, amino acids and vitamins/cofactors, which supply nutrients to the host. Specifically, *P. echinospica* could produce 4 vitamins/cofactors and 14 amino acids at least, whereas its symbionts can produce 13 vitamins/cofactors and 18 amino acids (Table 2). Nutrient interdependence between *P. echinospica* and its symbiont could be demonstrated by their cooperation in nutrient production, for example, the biosynthesis of methionine. Methionine that serves as an essential amino acid of most metazoans was detected in *Lamellibrachia* sp. and *Escarpia* sp. [83]. Currently, the endosymbionts of Siboglinidae cannot use cystathionine to synthesize methionine because of the lack of gene *metC* or *patB*, instead they use homoserine to synthesize methionine (Supplementary Fig. S12a). In *Paraescarpia* holobiont, host transcriptome contained the key genes (e.g., *CBS* and *serB*, etc.) responsible for producing cystathionine, and the symbiont genome contains *patB* and *metH* genes for using cystathionine to synthesize methionine (Supplementary Fig. S12b). Thus, we hypothesize that the *Paraescarpia* symbiont has the potential to use the cystathionine produced by the host to synthesize methionine for the holobiont use which indicates the complementary ability in nutrient production in *Paraescarpia* tubeworm holobiont.

**Table 2:** Capability of biosynthesis of amino acids, vitamins and cofactors in *Paraescarpia echinospica* and its symbiont

Nutrients	Description	Symbiont	*P. echinospica*
Biosynthesis of amino acids			
NEFAAs	A, C, D, E, G, P, Q, R, S, Orn	+	+
EFAAs	H, I, K, L, M, V, W, bA	+	−
	Ta, hypoTa, T, Y	−	+
	F, N	−	−
Biosynthesis of vitamins and cofactors			
Vitamin B1	Thiamine	+	−
Vitamin B2	Riboflavin	+	−
Vitamin B3	Nicotinate and nicotinamide	−	+
Vitamin B5	Pantothenate	+	+
Vitamin B6	Pyridoxine	+	+
Vitamin B7	Biotin	+	−
Vitamin B9	Folate	+	−
Vitamin B12	Cobalamin	−	−
Vitamin K2	Menaquinone	+	−
Coenzyme A	CoA	+	+
Coenzyme Q	Ubiquinone	+	−
	Protoheme (heme)	+	−
	Siroheme	+	−

Amino acids: A—Alanine; bA—β-Alanine; C—Cysteine; D—Aspartate (aspartic acid); E— Glutamic acid; F—Phenylalanine; G—Glycine; H—Histidine; hypoTa—Hypotaurine; I—Isoleucine; K—Lysine; L—Leucine; M—Methionine; N—Asparagine; Orn—Ornithine; P—Proline; Q—Glutamine; R—Arginine; S—Serine; T—Threonine; Ta—Taurine; V—Valine; W—Tryptophan; Y—Tyrosine; Complete and missing pathways are indicated by ‘+’ and ‘−’, respectively

**Fig. 5:**
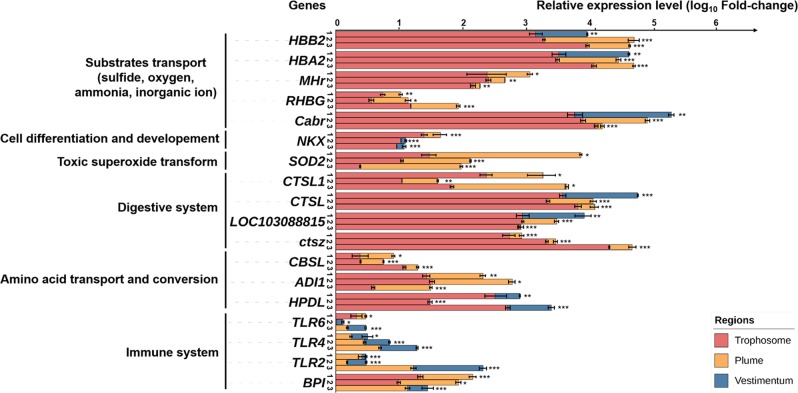
Real-time PCR results showing gene expression patterns among three regions: Red, trophosome; Yellow, plume; Blue, vestimentum. The *x*-axis was log_10_ scaled. The numbers 1, 2, 3 represent the number of tubeworm individuals. The full names of genes are shown in the list of abbreviations in Supplementary Information. (*P > 0.05, ** 0.01 < *P* < 0.05, ***P < 0.01)

The genome of the *P. echinospica* symbiont encoded only ten transporters, including transporters for minerals, polysaccharides and lipids (Supplementary Table S9), it had no substrate-specific transporters for amino acids and vitamins, suggesting that the *P. echinospica* symbiont cannot transport the nutrients to the host efficiently. The symbiont genomes of *Riftia pachyptila* [14], *Calyptogena* clam [84] and the Bathymodiolin mussel [85] encode few substrate-specific transporters as well, which suggests the symbionts are either leaky or digested by their host for nutrients. Furthermore, transcriptome and real-time PCR analyses of *P. echinospica* showed that several digestive enzymes were specific to the trophosome (Supplementary Table S11, Fig. 5), and these enzymes can aid in the digestion of symbionts [86]. Thus, in the tubeworm *P. echinospic*a, digestion of symbionts and carbon translocation from symbionts to the host are key processes by which the host acquires nutrients from the symbionts and controls the symbiont population.

### Virulence and nutrient acquisition of symbiotic bacteria

A large number of genes were found to encode proteases in the symbiont genome, and multiple bacterial proteinases were found expressed highly in the symbiont transcriptome (Table 3). The proteinases have broad specificity, as they can degrade host proteins, including those associated with immune response proteins such as various immunoglobulins, cytokines and chemokins, etc [87]. Notably, the *Paraescarpia* symbiont transcriptome and proteome contained high expression (2nd in transcriptome, 31th in proteome) of putative secreted esterase (PSE) (Fig. 3a, b), which is important in the bacterial virulence and pathogenesis [88] and may also function as a digestive enzyme for degradation of host animal cells [87]. Similarly, the symbiont transcriptome of *P. echinospica* contained the endochitinase ChiA (EC 3.2.1.14) and peptidase M48 (EC 3.4.24) responsible for the degradation of the structural barriers of the host by pathogens [89, 90] as well as various proteolytic enzymes in the symbiont proteome, such as the HtrA (DegP) serine protease (RLJ17779.1), peptidase M16 (RRS32426.1) and peptidase S41 (RDH90197.1). Serine protease is a cell envelope proteinase that can diminish function of the signal proteins manufactured by the host, it modulates the host immune response as a virulence factor by being anchored to the cell by sortase A that inactivates the complement factor of the host cell which is a key component of innate immune response [87, 91]. Cysteine proteases enhance bacterial ability to evade host innate immune response by degradation of host extracellular matrix material [87]. Thus, we hypothesize that the endosymbionts have the ability to modulate host immune response, degrade host cells and obtain nutrients by using their highly expressed proteases as virulence factors.

**Table 3:** Highly expressed bacterial proteinases in the endosymbiont of *Paraescarpia echinospica*

Transcript ID	Bacterial proteinases		TPM value
	Annotation	Description	
Pec_DN78589C0G1I1	Putative secreted esterase	Proteolysis; serine-type endopeptidase activity	99446.0
Pec_DN33952C0G1I1	Membrane protease subunit HflK	Peptidase activity; membrane; proteolysis; integral component of membrane	908.8
Pec_DN70443C1G1I1	ATP-dependent Lon protease	ATP-dependent Serine peptidase MEROPS family S16	427.9
Pec_DN73964C0G1I1	ATP-dependent metalloprotease	Cell division protease FtsH (*ftsH*, *hflB*)	397.0
Pec_DN5083C0G2I1	ATP-dependent Clp protease ATP-binding subunit	Peptidase activity; proteolysis; ATP-dependent peptidase activity	295.2
Pec_DN103576C0G1I1	ATP-dependent HslUV protease ATP-binding subunit HslU	HslUV protease complex; proteolysis; peptidase activity, acting on L-amino acid peptides	284.0
Pec_DN101171C0G1I1	ATP-dependent HslUV protease, peptidase subunit HslV	HslUV protease complex; proteolysis;	258.0
Pec_DN4612C0G1I1	Peptidase S49	*sppA*; protease IV; peptidase activity; proteolysis	239.6
Pec_DN49575C1G1I1	ATP-dependent chaperone	*clpB*; ATP-dependent Clp protease ATP-binding subunit ClpB	237.5
Pec_DN109493C0G1I1	Peptidase S41	Serine-type peptidase activity	234.0 s
Pec_DN26577C0G1I1	Peptidase C-terminal protease	Proteolysis; serine-type peptidase activity	225.1
Pec_DN49575C0G1I1	Disaggregation chaperone	*clpB*; ATP-dependent Clp protease ATP-binding subunit ClpB	223.7
Pec_DN11727C0G1I1	Cysteine protease	Proteolysis; cysteine-type peptidase activity	207.7
Pec_DN40710C0G1I1	Membrane protease subunit HflC	Peptidase activity; membrane; proteolysis; integral component of membrane	200.0
Pec_DN109409C0G1I1	Zn-dependent protease	Peptidase activity; proteolysis; chaperone-mediated protein folding	138.4

The symbiont genome of *P. echinospica* contained the OmpA-OmpF porin (OOP family) and OmpR families (OmpR-EnvZ and PhoP/Q systems) (Supplementary Table S12), which play important pathogenic roles such as bacterial adhesion and invasion in symbiotic-pathogenic bacteria [92–94]. These proteins can also promote bacterial intracellular survival and evasion of host defenses. Moreover, genes *ompA*, *ompR,* and *envZ* were highly expressed in the symbiont transcriptome and proteome (Fig. 3a, b). OmpR-EnvZ controls the bacterial virulence as a result of its ability to survive intracellularly [92] and PhoP/Q promotes bacterial resistance to the host’s innate immune response through bacterial surface modification [95]. The modified bacterial surface can enhance bacterial ability of immune evasion by the effects on activating factors and not activate host immune response [92, 93, 95]. These two are indispensable to the self-protection of bacteria after entering the host epidermal cells. Thus, the high expression of the OmpR-EnvZ system and OmpA proteins represents an adaptation in the symbionts that mediates host tolerance of symbiotic bacteria for their intracellular surviving, which may be critical for maintaining a stable symbiotic relationship in the *P. echinospica* holobiont. The bacterial infection process and proliferation post infection in *P. echinospica* are explained in the section of symbiont infection in Supplementary Information.

### Host innate immune responses

Unexpectedly, the expression level of immune-related genes in the bacteria-concentrated region of the trophosome was not higher than other two regions (Fig. 3c, Fig. 5). Among them, besides the bactericidal/permeability-increasing (BPI) protein highly expressed in the trophosome and plume as part of the innate immune system (Fig. 5) [96], Toll-like receptors (TLR2, TLR4, and TLR6) are key to the innate immune system and can recognize intruders and activate immune responses. TLR6 functionally interacts with TLR2 to mediate the cellular response to bacterial lipoproteins [97]. In the present study, expression level of the genes encoding TLRs was higher in the plume (the region in contact with free-living bacteria) and vestimentum than in the trophosome (Fig. 5). However, previous study showed the expression of TLR and PGRP genes in the trophosome was between five and 100-fold higher than that in the plume of vent-vestimentiferan *R. piscesae*, and expression of innate immunity genes in the trophosome of *R. piscesae* were higher than in the plume and as a whole may regulate the immune response to shape the symbiosis and to maintain symbiostasis [15]. Combining these observations with the above findings in the *Paraescarpia* endosymbionts, we speculate that the regulatory mechanism of *Paraescarpia* endosymbionts on host immunity make the trophosome possess a relatively weak immune system. More detailed information of the immune-related genes in the three regions is shown in the section on host innate immune responses in Supplementary Information (Supplementary Table S13). Consequently, we hypothesize that the endosymbiont of *Paraescarpia* has evolved elaborate strategies to distract the host’s protective immunity and evade its defenses.

## Conclusions

Our integrated genomic, transcriptomic, and proteomic analysis of the *P. echinospica* holobiont revealed metabolic, nutritional, and regulatory interdependencies in symbiosis, a key adaptation allowing the tubeworm to thrive in cold-seep chemosynthetic ecosystems. Genomic comparisons of vestimentiferan endosymbionts showed that the *Paraescarpia* symbionts had a high potential to evade the host immune response, reduce the toxicity of organic nitrogen compounds and transport proteins between cells. Analyses of the energy and nutrient pathways indicated a strong interdependence between *P. echinospica* and its symbionts in energy consumption and nutrient production. The bacterial symbionts may be able to degrade host proteins and use host cells as a nutrient source by using various virulence factors as digestive enzymes. The symbiont of *Paraescarpia* is believed to have evolved strategies to mediate host innate immunity as immune response genes performed not prominently as expected in the trophosome. Our findings suggest that the maintenance of host-microbiota dynamics is determined by the holobiont’s evolved interdependence, which provides a new insight into the adaptation of deep-sea chemosynthetic holobionts.

## Supplementary information

Supplementary Information

Supplementary Excel Table S4

Supplementary Excel Table S5

Supplementary Excel Table S8
