# Supplementary material for: Genomic, transcriptomic, and proteomic insights into the symbiosis of deep-sea tubeworm holobionts
Source: ISME J. 2019 Oct 8;14(1):135–50. doi: 10.1038/s41396-019-0520-y (PMC6908572; doi:10.1038/s41396-019-0520-y)
Supplement: Supplementary file 1 — Supplementary Information [file 41396_2019_520_MOESM1_ESM.docx]

Supplementary Information for:

**Genomic, transcriptomic and proteomic insights into the symbiosis in deep-sea tubeworm holobionts**

**Authors:** Yi Yang^1^, Jin Sun^1^, Yanan Sun^1^, Yick Hang Kwan^1^, Wai Chuen Wong^1^, Yanjie Zhang^2^, Ting Xu^2^, Dong Feng^3,4^, Yu Zhang^5^, Jian-Wen Qiu^2*^, Pei-Yuan Qian^1*^

Materials and Methods

***Environmental factors***

In South China Sea, *Paraescarpia echinospica* commonly occurs in the outskirts of the cold seep macrobenthic communities dominated by the bathymodiolin mussels *Gigantidas haimaensis* [1] or *Gigantidas platifrons* (previous called *Bathymodiolus platifrons*) [2]. Zhang *et al*. (2017) used a Raman Insertion Probe to measure water and sediment chemistry at Site F (also known as Formosa Ridge) [3]. They found as high as 1.54 mM CH_4_, but no SO_4_^2-^in the diffuse fluid underneath the Gigantidas-Shinkaia community. In the outskirt of the chemosynthetic community, they found sulfide concentration to decline from ~10 mM at 60 cm below sediment surface (bss), to ~2mM at 10 cm bss. However, no data are available on the chemical composition of the sediment pore water or surface water at the sampling site for *P. echinospica*, which is also ~ 60 m from the center of the Gigantidas-Shinkaia community. So we refer to the study of Zhang *et al*. (2017) and give a rough estimate of the local geochemical environment of the sampling site.

***Microbial phylogenetic analysis and cophylogenetic analysis of holobionts***

To determine the microbial community composition of the tubeworm, approximately 1,500-bp 16S rRNA gene fragment was amplified by PCR from the trophosome using universal bacterial primers 8f and 1492r [4]. A clone library of 16S rRNA gene was constructed, and 30 clones were randomly picked up from the library and sequenced. The resultant sequences were analyzed using RDP Naive Bayesian rRNA Classifier v2.11 [5] with an 80% confidence threshold to reveal prokaryotic species composition in the trophosome. A phylogenetic tree was constructed based on 16S rRNA gene sequences, approximately 1,500 bp of 16S rRNA gene sequence was predicted from the binned genome using RNAmmer (version 1.2) [6], 16s rRNA gene sequences from other bacteria were obtained from GenBank. To understand the phylogenetic position of the *Paraescarpia* symbiont, it’s 16S rRNA gene sequence, and the 16S rRNA gene sequences of 49 other symbiotic marine bacteria downloaded from GenBank, were used to construct a phylogenetic tree using the Maximum Likelihood (ML) method, with 1000 bootstrap replicates [7]. The sequences were aligned in MEGA 7 using the Muscle algorithm [8]. The GTR+F+I+G4 model was selected based on the result of ModelFinder [9], and the phylogenetic tree was generated using IQ-TREE version 1.6.10 [10].

Transcriptome data of *Riftia pachyptila*, *Ridgeia piscesae*, *Escarpia spicata*, *Lamellibrachia luymesi*, *Seepiophila jonesi* and *Galathealinum* *brachiosum* were obtained from the NCBI SRA database (Table S1), SRA data files were converted to fastq format using SRA Toolkit version 2.9.6-1 (<https://trace.ncbi.nlm.nih.gov/Traces/sra/sra.cgi?view=software>). After checking and removing bacterial reads, the Illumina paired-end and 454 transcriptome data were assembled using Trinity version v2.8.5 [11], under default settings (‘--trimmomatic’ for running Trimmomatic-0.39 [12] to quality trim reads, ‘--single’ for 454 single reads and ‘--left --right’ for Illumina paired-end reads). Only the highest expressed isoforms were retained. CD-HIT-EST [13] was used to further reduce redundant sequences with a threshold of 90% similarity. Transcript expression levels were quantified and expressed in transcripts per million (TPM) using Salmon [14]. The transcripts with at least 10 parts per million (ppm) were retained [15]. TransRate [16] was used to detect errors such as chimeric artifacts, incomplete assembly and base errors in the assembled tubeworm transcriptome. TransDecoder [17] was used to detect coding regions. BUSCO v3 was used to evaluate the duplicates of the tubeworm transcriptome assembly [18]. The transcriptome data of *Paraescarpia echinospica* was from this study. Transcriptomes of the above seven siboglinid tubeworms are used in phylogenomic analyses. OrthoMCL [19] was applied to identify ortholog groups (OGs) from each transcriptome (BLAST threshold E = 1 × 10^-5^). Only single-copy genes in each OG that were found in at least 6 taxa were retained for phylogenomic analysis, resulting in 408 OGs among the 7 species. Sequences of each OG were aligned using MUSCLE and trimmed using TrimAL [20]. After concatenating these alignments, the phylogenetic tree was constructed using RaxML version 8.2.4 [21] under the GTR+Γ model with the partition information of each orthologous gene and 1,000 bootstrap replicates (Figure S6). The corresponding phylogenomic analysis of the endosymbionts of siboglinids have been included in the main text (Figure 1a).

***Holobiont transcriptome analysis***

Blast2GO v4.0.7 [22] was applied to assign Gene Ontology (GO) terms for the transcripts, and REVIGO was used to summarize and visualize the long list of GO terms [23]. Transcript expression levels in each region (plume, vestimentum and trophosome) were quantified respectively and expressed as Transcripts Per Million (TPM) using Salmon [24]. A gene was considered to be specific to a particular region if its TPM value in the region accounted for more than 75% of the total TPM of all three regions [25]. Based on two sets of trophosomal sequencing data, the annotation of complete meta-transcriptomic data set from both symbiont (trophosome) and host is shown in Excel Table S8-1 and 8-2, and the annotation of another host transcriptomic data set from the trophosome is shown in Supplementary Excel Table S8-3. Statistically overrepresented GO terms in the different regions were identified through Gene Ontology Enrichment Analysis Software Toolkit (GOEAST) [26]. WEGO (http://wego.genomics.org.cn/cgi-bin/wego/index.pl) was used to plot GO annotations of highly expressed genes (TPM ratio > 0.75) in the plume, vestimentum and trophosome. A phylogenetic tree was constructed based on potential hemoglobin protein sequences from marine worms, approximately 78 bp of hemoglobin subunit sequences from *P.* *echinospica* were obtained from the transcriptome of *P.* *echinospica* using BLASTP with an *E* value of 1×10^-5^, hemoglobin sequences from other marine worms were obtained from GenBank. Phylogenetic analyses were conducted using the Maximum Likelihood (ML) method, with 1000 bootstrap replicates [6]. The sequences were aligned in MEGA 7 using the Muscle algorithm [27]. The WAG+I+G4 model was selected based on the result of ModelFinder [8], and the phylogenetic tree was generated using IQ-TREE version 1.6.10 [9].

***Proteomic approach***

Protein extraction, SDS-PAGE, and in-gel trypsin digesion

The trophosome from three *P. echinospica* was dissected and fixed in a lysis buffer (8M urea, 40mM HEPES, pH=8.0), and sonicated by QSonica (Newtown, CT, USA). Afterwards, the sample was centrifuged at 15,000 g for 15min, and the supernatant was further purified with protein 2-D cleanup kit (Bio-Rad, Hercules, CA, USA). Approximately 30 µg of protein was subjected to SDS-PAGE on a 4-12% gradient gel, and the gel was stained by colloidal coomassie blue [28]. Then, the SDS-PAGE gel was cut into ten slices and dehydrated by 100mM NH_4_HCO_3_, 50mM NH_4_HCO_3_ and 50% ACN, and 100% ACN. Protein was reduced by 10mM DTT for 45 mim at 56^o^C and alkylated by 55mM iodoacetamide for 20 min in dark. Protein was digested by sequencing grade Trypsin (Promega) for 14 hours at 37^o^C. The peptide was extracted using 5% formic acid in 50% ACN and 100% ACN sequentially, and the extract was pooled, dried in a speed-vacuum and desalted with a C18 Sep-Pak column (Waters).

LC-MS/MS

Each fraction was reconstituted in 20 μL of 0.1% formic acid and analysed by a Dionex UltiMate 3000 RSLCnano coupled with an Orbitrap Fusion Lumos Mass Spectrometer (Thermo Fisher). The following 120min mobile phase mixing gradient was set in nanoLC: an initial 10min from 0% (i.e. 100% mobile phase A, 0.1% formic acid in H_2_O) to 2% mobile phase B (0.1% formic acid in ACN), 2min from 2% to 6% B, 70min from 6% to 20% B, 10min from 20% to 30% B, 8min from 30% to 100% B, 5min at 100% B and 15min at A. The flow rate was controlled at 300nL/min. The following settings was set in Orbitrap: positive ion mode, MS resolution: 60,000, scan range: 400−1500m/z, AGC target: 4.0e5, dynamic exclusion duration: 40s, HCD in MS/MS, HCD collision energy: 30%, isolation window: 1.6m/z, and first mass: 110 m/z.

Database search

The raw mass spectrometry data was converted to .*mgf* file by 1.1.0.18 and submitted to Mascot version 2.3.0 for protein identification and quantification. The searched reference database includes the *P. echinospica* translated protein database, its endosymbiont protein database and their corresponding reversed sequences which was served as decoy, respectively. The searching criteria was set as: 5ppm for precursor, 0.6 Da for fragments, fixed modification: carbamidomethyl (cysteine), variable modification: oxidation (methionine), and up to two missed trypsin cleavage was allowed. Peptide with an expectation level less than 0.05 was removed, and the false discovery rate for the protein identification was dynamically controlled as 1%. All sequences used in databases were obtained from the *Paraescarpia* holobiont meta-transcriptome.

***Real-time PCR validation***

The primers for real-time PCR (Table S2) were designed using the online NCBI Primer-BLAST tool (https://www.ncbi.nlm.nih.gov/tools/primer-blast/). PCR product length was set within the range of 100bp – 200bp, and optimal melting temperature was set as 60.0 °C. Only Primer-pair with the least possibilities of self-complementarity and self 3' complementarity was selected for each gene. The eukaryotic translation initiation factor (*Eif3f*) was selected as the internal standard gene, as its expression level remain almost constant across all the samples. Real-time PCR was performed on LightCycler 480 II (Roche) with the following procedures: 1) polymerase activation at 95 °C for 10 min, and 2) annealing and extending at 57 °C for 1 min with a total of 40 cycles. The specificity of primer pairs for the PCR amplification was checked by the melting curve method. Negative controls were used to exclude non-specific amplification of DNA contamination.

***Correlation of proteomic data and RNA profiling.***

Gene expression level of *Paraescarpia* holobiont was evaluated by the transcriptome and real-time PCR. The abundances of protein were obtained from proteomic data. In order to test the degree of similarity between gene expression and protein concentration of the trophosome, relationship of real-time PCR and proteome data was assessed by the Spearman rank coefficient (rs) analysis of relative gene expression levels (Log_10_ Fold-change) and relative protein abundances (Log_10_ emPAI%), emPAI% indicates the percentage of a specific protein’s emPAI value in the sum of total proteins’ emPAI values. The correlation between real-time PCR and proteome data is shown in Figure S7a. In addition, the total annotated 425 proteins of the *P. echinospica* symbiont and the percentage of their functional categories is shown in the Figure S7b, in the same way, the total annotated 831 transcripts of the *P. echinospica* symbiont is shown in the Figure S7b. The Venn diagram of the total 474 proteins and the 474 most highly expressed transcripts of the *P. echinospica* symbiont is shown in Figure S7c, indicating the overlap ratio of transcriptomic and proteomic data. The correlation between transcriptome and proteome data was assessed by the Spearman rank coefficient (rs) analysis of relative gene expression levels (Log_10_ TPM) and relative protein abundances (Log_10_ emPAI%).

Results and Discussion

***Microbial phylogeny and holobionts cophylogeny analysis.*** Analyzing 30 clones of 16S rRNA gene revealed high similarity (99.9-100%) among them, indicating there was only one bacterial species in *P. echinospica* trophosome. A phylogenetic tree constructed using 16S rRNA gene sequences from 50 species shows that the endosymbiotic bacteria of *P. echinospica* belongs to the class *γ-Proteobacteria*, being nested in a clade containing several endosymbionts of seep tubeworms (i.e., *Lamellibrachia*, *Seepiophila* and *Escarpia*) (Figure S4). Among them, the *Escarpia southwardae* endosymbiont had the highest sequence similarity (99.9%) with that of *P. echinospica.* Comparing with the assembled genomes of seven tubeworm endosymbiont genomes (Table 1), the result showed that the assembled *Paraescarpia* symbiont genome had much higher continuity and was better than other assemblies because a combination of short Illumina and long ONT reads was used in our study.

The phylogenomic tree of siboglinid tubeworms showed the same topology with the phylogenetic tree based on 13 mitochondrial genes (Figure 1a, Figure S6) which is also consistent with the phylogenomic tree of Siboglinidae in previous study [29]. Our phylogenomic analysis of siboglinid holobionts showed that bacterial symbionts and their associated tubeworm host did not co-speciate.

***Substrates supply and energy conversion.*** Genes responsible for substrates transportation include a myohemerythrin (*MHr*), hemoglobin subunits (*HBA1*, *HBA2*, *HBB1*, and *HBB2*) and a phosphoenolpyruvate carboxykinase (EC 4.1.1.32) (*PEPCK*) in the host, and ones related to carbon fixing (*rubisco*), sulfur oxidation (*dsrAB*), the sulfur storage compound (*sgpA*), and nitrogen and hydrogen metabolisms (*nosZ* and *hppA*) in the symbiont. Previous studies have shown that the deep-sea tubeworm symbionts have the ability to adjust the relative expression of each pathway depending on the prevailing geochemical and energetic environment [30, 31]. The expression of different metabolic pathways in the *P. echinospica* symbiont was demonstrated by its transcriptome and proteome. Furthermore, the genes related to molecular chaperones and bacterial nucleoid-associated proteins were highly expressed in the symbiont (Figure 3a and 3b), which included the chaperonins GroEL, DnaK, HtpG and HscA, the DNA-binding transcriptional activator GlrR and the transcription elongation factor GreA. These genes might have a symbiosis-specific function in the *Bathymodiolus azoricus* symbionts [32] and thus, we suspect they have similar functions in *P. echinospica*.

From GO annotation, number and percentage of highly expressed genes in the plume, vestimentum and trophosome are shown in Figure S8, genes related to electron carrier, enzyme regulation, transporter, establishment of localization and metabolic process were more in the trophosome than in other two regions. To see more characterized genes associated with symbiosis, highly expressed genes in the trophosome (the symbiont-harbouring region) were paid attention. Metabolic genes take the largest proportion of highly expressed genes in the trophosome (Figure S9), which shows energy production and conversion, metabolites biosynthesis, transport and catabolism occur actively in the trophosome. For instance, genes for the hemoglobin complex, oxygen transporter activity and oxygen binding were all highly expressed and significantly enriched in the trophosome as compared to the plume and vestimentum (Figure S10, Table S10), indicating that *P. echinospica* actively transported oxygen and substrates to its sulfur-oxidizing symbionts for energy conversion in the trophosome, which is key to support chemosynthesis [33]. Vestimentiferans have three types of extracellular hemoglobins (Hbs), vascular V1, V2 and coelomic C1, which are built by different associations of four globin subunits and linker chains [34]. Expression of Hb transcripts in the plume, vestimentum and trophosome of *P. echinospica* show that Hb subunit A1, A2, B1, B2 and linker L1, L4 were all expressed higher in the trophosome than the other two regions (Figure3 and Figure 5). Linker chains of V1 Hb and globin subunits A2, B2 are H_2_S-binding, globin subunits A1 and B1 are O_2_-binding [34, 35], which make three different Hbs function as oxygen-sulfide transporters. The above results confirmed Hbs were significant transporters in *P. echinospica*, supplying the chemosynthetic symbionts in the trophosome with oxygen and sulfide. In the trophosome, the number of highly expressed Hb subunit A1, B1 transcripts is about three times that of V1 Hb linker chains and Hb subunit A2, B2, ensuring sufficient oxygen to symbionts for various oxidation reactions. The phylogenetic analysis of potential Hb subunits sequences from Polychaeta marine worms is shown in Figure S11.

Genes involved in energy production and conversion account for the largest proportion of the total number of the most expressed genes in the host and symbionts (Figure 3a and 3b), indicating their significance to symbiotic relationships. The high expression of sulfur metabolic genes in the symbiont, especially *aprAB* and *dsrAB*, supports the notion that sulfur oxidation serves as the symbiont’s main energy source (Figure 4). Our genomic results show that the endosymbionts of *P. echinospica* can use thiosulfate as an alternative sulfur source in addition to sulfide, which can be viewed as an adaptation to the reduced sulfide supply in its habitat [30, 36]. However, our transcriptomic and proteomic results showed that only genes in the sulfide oxidation pathway (i.e., *aprAB* and *dsrAB*) were highly expressed, indicating under the field conditions, the Sox thiosulfate oxidation pathway was not activated. Two NiFe hydrogenases (*hupL*, *hupS*) were found in the genome the *P. echinospica* symbiont, indicating that the *P. echinospica* symbiont has the ability to utilize hydrogen as an additional energy source, which is widespread in deep-sea tubeworm symbionts [37]. In concordance with the high abundance of nitrate and ammonium in methane seeps [38], the genes responsible for denitrification (i.e., *napAB*, *norBC* and *nosZ*) and ammonia assimilation (i.e., *glnA* and *gltBD*) in the *P. echinospica* symbiont genome were highly expressed (Figure 4; Supplementary Excel Tables S8-1), indicating that the *P. echinospica* symbiont can help the host to assimilate ammonia into glutamate for nutrition (Figure 4). In addition, the symbionts may use nitrate or nitrite as an alternative electron acceptor for sulfide oxidation under hypoxic conditions [36], the sulfur-driven autotrophic denitrification process can certainly help *P. echinospica* consume the accumulated hydrogen sulfide and nitrates (or nitrites).

***Mixotrophic ability of symbionts.*** Similar to heterotrophic symbionts of deep-sea *Osedax* worms [39], the *P. echinospica* symbiont possesses 35 genes responsible for flagellar assembly indicating the capability for movement and bacterial chemotaxis (Table S12). Also, the *P. echinospica* symbiont has 21 genes for developing a complex chemosensory system, including *fli* genes that encode flagellar motor switch proteins, *che* and *mot* genes that encode chemotaxis proteins, *mcp*, *tsr*, *tar*, *trg* and *tap* genes that encode methyl-accepting chemotaxis proteins (MCPs) (Table S12). MCPs have been known for bacterial extracellular signal recognition and chemotaxis initiation in *Escherichia coli* [40], indicating complex interactions between the symbiont and external environment. Nevertheless, only few genes (such as *mcp*, *cheBR* and *fliKS*) associated with bacterial chemotaxis and flagellar assembly were expressed in the *P. echinospica* symbiont transcriptome (Figure 4, Excel Table S8-1), indicating that these genes may only be active at free-living stage. Besides, genes encoding the complete pathways of formate oxidation, glycolysis and citrate cycle were identified in the *P. echinospica* symbiont genome (Figure 4, Table S9), showing the symbiont’s potential ability of oxidizing organic substrates. The capacity to oxidize formate was recently discovered in the endosymbionts of *Escarpia* sp. and *Lamellibrachia* sp.2 [41], however, the three published vent-living tubeworm endosymbionts [42, 43] cannot oxidize formate without *FDH* gene, suggesting the seep species are more adaptable to different carbon sources, such as formate-rich habitats. Furthermore, transporters of capsular polysaccharide, lipopolysaccharide and lipoprotein, enzymes of carboxypeptidase, lipases and glycosidases were found in the *P. echinospica* symbiont genome (Table S9), indicating the symbiont has the potential to use sugars, peptides or lipids. The abilities to organic substrates utilization and transportation indicate the *P. echinospica* symbiont can survive as a heterotroph, and is not purely an autotroph. Taken together, our results indicate the *P. echinospica* symbiont genome has no genomic reduction and even larger than other vestimentiferan endosymbionts (Table 1), but many genes are not expressed in its symbiotic stage that only be useful in their free-living stage, such as genes for bacterial chemotaxis, flagellar assembly and organics utilization. A recent study of *Lamellibrachia* and *Escarpia* symbionts shows their high metabolic diversity was consistent with their relatively large genome size compared to the endosymbiont genomes of vent vestimentiferans [41], supporting our conclusion that the *P. echinospica* symbiont is a mixotroph with a high metabolic flexibility and may help the holobiont better survive in various habitats.

***Storage compounds***. Cyanophycin (CGP) as a cellular carbon and nitrogen storage compound synthesized by Cyanophycin synthetase (*cphA*) were first found in the genome of deep-sea symbionts of *Ridgeia*, *Riftia* and *Tevnia* [42]. However, the absence of *cphA* gene in the *P. echinospica* symbiont genome indicate the inability of CGP production. Meanwhile, we found the sulfur globule protein (Sgp) was expressed highly in the symbiont transcriptome (Figure 3a, Table S8-1). The formation of intracellular sulfur globules in many sulfide-oxidizing bacteria during oxidation of reduced sulfur compounds when food is in short supply, sulfur has also been reported to produce inside the cell as a protective, detoxifying reaction [44]. Therefore, we conclude that the highly expressed sulfur globules in the *P. echinospica* symbiont can help the host cope with a nutritionally deficient environment in Haima cold seep even if the symbiont don't have CGP as a storage compound.

***Symbiont infection and host innate immune responses.*** Previous studies of Gram-negative bacterial pili, fimbriae and flagellum show these organelles were key structures for bacterial adherence to potential host organism, they can aid bacterial colonization of mucosal surface and their virulence-associated functions contribute to the infection pathogenesis after the first step of surface colonization [45]. The *P. echinospica* symbiont possesses the genes encoding type IV pili, type IV fimbriae and flagellum (Table S12), we consider these organelles function as a key virulence factor in the *P. echinospica* symbiont. For instance, in Vibrio cholerae and other pathogenic bacteria, type IV pili are multifunctional adhesive structures that participate in adhesion to eukaryotic epithelial cells, and allow bacterial translocation over the infected tissue [45], fimbriae can bind to specific structures on host cells as well as various connective tissue proteins, they acted as an invasion factor through mediating bacterial adhesion and subsequent events [45], which promote bacterial adherence to epidermal cells of *P. echinospica* larvae and contribute to regulate immunity and pathogenesis during bacterial infections. In our study, over 10 CDSs annotated with bacterial adhesin/invasin, surface adhesion protein and biofilm synthesis protein were found in the *P. echinospica* symbiont genome (Table S12), these proteins may facilitate the bacteria colonize a mucous coat of *P. echinospica* larvae before taken up across epidermal cells [42, 46]. Also, the surface protein transpeptidase sortase (srtA) in seep-unique genes was found to be required for adhesion to host epithelial cells and for colonization of the host intestine [47]. After entering host cells, the inter- and intracellularly migration of *P. echinospica* symbiont to the new developed trophosome would be mediated by some characterized proteins, for example, a putative hemolysin III homolog (*hlyIII*), a lysine 2,3-aminomutase (*kamA*) and the colicin V production protein (*cvpA*) were found in the symbiont genome (Table S12), which might enable the bacteria to change the permeability of the cell membrane [42, 48]. The *P. echinospica* symbiont genome contains abundant genes for DNA replication and cellular division (Table S12), and these genes were also found in the transcriptome analyses, indicating the capability for proliferation in the host post infection.

In concordance with the fact that trophosome hosts endosymbionts, we detected immune related genes expressed in this region (Figure 3c and Table S13), even if they are not active as expected. The genes of peptidoglycan recognition proteins (PGRP-2 and PGRP-SC2), which can bind to peptidoglycans of bacteria and trigger the prophenoloxidase cascade immune response [49] were expressed in the trophosome and vestimentum. PGRPs can mediate the symbiont tolerance, symbiont population control and regulation of symbiosis establishment and maintenance [50]. Also, seven different classes of lectins were detected in the plume and trophosome of *P. echinospica* (Supplementary Table S13), which stimulate immune system response by aiding in self-nonself discrimination and defensing against invading microorganisms or modulating interactions between the host and its beneficial symbionts [50]. For instance, F-type lectins and L-type lectins were highly expressed only in the trophosome, indicating their involvement in symbiont acquisition and maintenance in *P. echinospica* [50, 51]. For genes that control immune responses, genes of NF-κB inhibitor and NF-κB p65 were highly expressed in the plume and vestimentum, NF-κB plays key role in the immune system in response to microbe-associated molecular patterns (MAMPs) since NF-κB and macrophage migration inhibitory factor are often involved in downstream cell signaling cascades [52]. The genes responsible for scavenger receptor cysteine rich (SRCR) domains, such as epidermal growth factor, low density lipoprotein (LDL) receptor and immunoglobulin superfamily (lgSF), were found expressed highly in the trophosome. The proteins contain SRCR domains are often associated with host-symbiont interaction and MAMP recognition [53] in invertebrates. Also, the genes responsible for the cell cycle control and death [52], such as apoptosis inhibitor 5, programmed cell death 8, ubiquitin-like protein, apoptosis inhibitor IAP1 and mediator of apoptosis DAP3, were found in the trophosome of *P. echinospica*, with some of them highly expressed, such as apoptosis inhibitor IAP1 and BIR domain proteins.

***Correlation of proteomic data and RNA profiling.*** A significant positive correlation (r_s_ = 0.783, P = 0.013) of real-time PCR and proteome data shows high similarity of gene expression and protein concentration of the trophosome (Figure S7a). The percentages of functional categories of proteins and transcripts are highly similar in the *Paraescarpia* symbiont, indicating the similar gene expression pattern of transcriptome and proteome (Figure S7b). In addition, overlap ratio of the 475 most highly expressed transcripts and proteins is more than 50% (Figure S7c), among them, 20 shared transcripts/proteins were randomly selected to test the degree of similarity between gene expression and protein concentration of the symbiont. A significant positive correlation (r_s_ = 0.830, P = 0.000) of transcriptome and proteome data shows highly similarity of gene expression and protein concentration of the symbiont (Figure S7d).

Supplementary information is available at *the ISME Journal*’s website.

References

1. Xu T, Feng D, Tao J and Qiu JW. A new species of deep-sea mussel (Bivalvia: Mytilidae: Gigantidas) from the South China Sea: morphology, phylogenetic position, and gill-associated microbes. *Deep Sea Res Part I Oceanogr Res Pap* 2019; **146**: 79–90.

2. Feng D, Qiu JW, Hu Y, Peckmann J, Guan H, Tong H *et al.* Cold seep systems in the South China Sea: An overview. *J Asian Earth* Sci 2018; **168**: 3–16.

3. Zhang X, Du Z, Zheng R, Luan Z, Qi F, Cheng K *et al*. Development of a new deep-sea hybrid Raman insertion probe and its application to the geochemistry of hydrothermal vent and cold seep fluids. *Deep Sea Res Part 1 Oceanogr Res Pap* 2017; **123**: 1-12.

4. Paster BJ, Bartoszyk IM, Dewhirst FE. Identification of oral streptococci using PCR-based, reverse-capture, checkerboard hybridization. *Methods Cell Sci* 1998; **20**: 223–231.

5. Wang Q, Garrity GM, Tiedje JM, Cole JR. Naive Bayesian classifier for rapid assignment of rRNA sequences into the new bacterial taxonomy. *Appl Environ Microbiol* 2007; **73**: 5261–5267.

6. Lagesen K, Hallin P, Rodland EA, Staerfeldt HH, Rognes T, Ussery DW. RNAmmer: consistent and rapid annotation of ribosomal RNA genes. *Nucleic Acids Res* 2007; **35**: 3100–3108.

7. Hoang DT, Chernomor O, von Haeseler A, Minh BQ, Vinh LS. UFBoot2: improving the ultrafast bootstrap approximation. *Mol Biol Evol* 2018; **35**: 518–522.

8. Kumar S, Stecher G, Tamura K. MEGA7: Molecular Evolutionary Genetics Analysis version 7.0 for bigger datasets. *Mol Biol Evol* 2016; **33**: 1870-1874.

9. Kalyaanamoorthy S, Minh BQ, Wong TKF, von Haeseler A, Jermiin LS. ModelFinder: fast model selection for accurate phylogenetic estimates. *Nat Methods* 2017; **14**: 587–589.

10. Trifinopoulos J, Nguyen LT, von Haeseler A, Minh BQ. W-IQ-TREE: a fast online phylogenetic tool for maximum likelihood analysis. *Nucleic Acids Res* 2016; **44**: W232–W235.

11. Grabherr MG, Haas BJ, Yassour M, Levin JZ, Thompson DA, Amit I *et al.* Full-length transcriptome assembly from RNA-Seq data without a reference genome. *Nat Biotech* 2011; **29**: 644–652.

12. Bolger AM, Lohse M, Usadel B. Trimmomatic: a flexible trimmer for Illumina sequence data. *Bioinformatics* 2014; **30**: 2114–2120.

13. Li W, Godzik A. Cd-hit: a fast program for clustering and comparing large sets of protein or nucleotide sequences. *Bioinformatics* 2006; **22**: 1658–1659.

14. Patro R, Duggal G, Love MI, Irizarry RA, Kingsford C. Salmon provides fast and bias-aware quantification of transcript expression. *Nat Methods* 2017; **14**: 417–419.

15. Sun J, Zhang Y, Xu T, Zhang Y, Mu HW, Zhang YJ *et al.* Adaptation to deep-sea chemosynthetic environments as revealed by mussel genomes. *Nat Ecol Evol* 2017; **1**: 0121.

16. Smith-Unna R, Boursnell C, Patro R, Hibberd JM, Kelly S. TransRate: reference-free quality assessment of *de novo* transcriptome assemblies. *Genome Res* 2016; **26**: 1134–1144.

17. Haas BJ, Papanicolaou A, Yassour M, Grabherr M, Blood PD, Bowden J *et al.* De novo transcript sequence reconstruction from RNA-seq using the Trinity platform for reference generation and analysis. *Nat Protocols* 2013; **8**: 1494–1512.

18. Simão FA, Waterhouse RM, Ioannidis P, Kriventseva EV *et al*. BUSCO: assessing genome assembly and annotation completeness with single-copy orthologs. *Bioinformatics* 2015; **31**: 3210–3212.

19. Li L, Stoeckert CJ & Roos DS. OrthoMCL: identification of ortholog groups for eukaryotic genomes. *Genome Res* 2003; **13**: 2178–2189.

20. Capella-Gutierrez S, Silla-Martinez JM, Gabaldon T. trimAl: a tool for automated alignment trimming in large-scale phylogenetic analyses. *Bioinformatics* 2009; **25**: 1972-1973.

21. Stamatakis A, Ludwig T, Meier H. RAxML-III: a fast program for maximum likelihood-based inference of large phylogenetic trees. *Bioinformatics* 2005; **21**: 456-463.

22. Conesa A, Gotz S, Garcia-Gomez JM, Terol J, Talon M, Robles M. Blast2GO: a universal tool for annotation, visualization and analysis in functional genomics research. *Bioinformatics* 2005; **21**: 3674–3676.

23. Supek F, Bosnjak M, Skunca N, Smuc T. REVIGO summarizes and visualizes long lists of gene ontology terms. *Plos One* 2011; **6**: e21800.

24. Patro R, Duggal G, Love MI, Irizarry RA, Kingsford C. Salmon provides fast and bias-aware quantification of transcript expression. *Nat Methods* 2017; **14**: 417–419.

25. Sun J, Zhang Y, Xu T, Zhang Y, Mu HW, Zhang YJ *et al.* Adaptation to deep-sea chemosynthetic environments as revealed by mussel genomes. *Nat Ecol Evol* 2017; **1**: 0121.

26. Zheng Q, Wang XJ. GOEAST: a web-based software toolkit for Gene Ontology enrichment analysis. *Nucleic Acids Research* 2008; **36**: W358–W363.

27. Kumar S, Stecher G, Tamura K. MEGA7: Molecular Evolutionary Genetics Analysis version 7.0 for bigger datasets. *Mol Biol Evol* 2016; **33**: 1870–1874.

28. Sun J, Zhang H, Wang H, Heras H, Dreon MS and Ituarte S *et al*. First proteome of the egg perivitelline fluid of a freshwater gastropod with aerial oviposition. *J Proteome Res* 2012; **11**: 84240–4248.

29. Li Y, Kocot KM, Whelan NV, Santos SR, Waits DS, Thornhill DJ et al. Phylogenomics of tubeworms (Siboglinidae, Annelida) and comparative performance of different reconstruction methods. *ZOOL SCR* 2016; **46**: 200–213.

30. Gardebrecht A, Markert S, Sievert SM, Felbeck H, Thurmer A, Albrecht D et al. Physiological homogeneity among the endosymbionts of *Riftia pachyptila* and *Tevnia jerichonana* revealed by proteogenomics. *ISME J* 2012; **6**: 766–776.

31. Markert S, Gardebrecht A, Felbeck H, Sievert SM, Klose J, Becher D *et al.* Status quo in physiological proteomics of the uncultured *Riftia pachyptila* endosymbiont. *Proteomics* 2011; **11**: 3106–3117.

32. Zierenberg RA, Adams MWW, Arp AJ. Life in extreme environments: Hydrothermal vents. *Proc Natl Acad Sci USA* 2000; **97**: 12961–12962.

33. Zal F, Leize E, Lallier FH, Toulmond A, Van Dorsselaer A, Childress JJ. S-sulfohemoglobin and disulfide exchange: the mechanisms of sulfide binding by *Riftia pachyptila* hemoglobins. *Proc Natl Acad Sci USA* 1998; **95**: 8997–9002.

34. Ponnudurai R, Kleiner M, Sayavedra L, Petersen J, Moche M, Otto A *et al*. Metabolic and physiological interdependencies in the Bathymodiolus azoricus symbiosis. *ISME J* 2016; **11**: 463–477.

35. Bailly X, Leroy R, Carney S, Collin O, Zal F, Toulmond A *et al.* The loss of the hemoglobin H_2_S-binding function in annelids from sulfide-free habitats reveals molecular adaptation driven by Darwinian positive selection. *Proc Natl Acad Sci USA* 2003; **100**: 5885–5890.

36. Markert S, Gardebrecht A, Felbeck H, Sievert SM, Klose J, Becher D et al. Status quo in physiological proteomics of the uncultured Riftia pachyptila endosymbiont. *Proteomics* 2011; **11**: 3106–3117.

37. Li Y, Liles MR, Halanych KM. Endosymbiont genomes yield clues of tubeworm success. *ISME J* 2018; **12**: 2785–2795.

38. Bowles M, Joye S. High rates of denitrification and nitrate removal in cold seep sediments. *ISME J* 2011; **5**: 565–567.

39. Goffredi SK, Yi H, Zhang Q, Klann JE, Struve IA, Vrijenhoek RC *et al.* Genomic versatility and functional variation between two dominant heterotrophic symbionts of deep-sea *Osedax* worms. *ISME J* 2014; **8**: 908–924.

40. Wadhams GH, Armitage JP. Making sense of it all: Bacterial chemotaxis. *Nat Rev Mol Cell Bio* 2004; **5**: 1024–1037.

41. Reveillaud J, Anderson R, Reves-Sohn S, Cavanaugh C, Huber JA. Metagenomic investigation of vestimentiferan tubeworm endosymbionts from Mid-Cayman Rise reveals new insights into metabolism and diversity. *Microbiome* 2018; **6**: 19.

42. Gardebrecht A, Markert S, Sievert SM, Felbeck H, Thurmer A, Albrecht D *et al.* Physiological homogeneity among the endosymbionts of *Riftia pachyptila* and *Tevnia jerichonana* revealed by proteogenomics. *ISME J* 2012; **6**: 766–776.

43. Perez M, Juniper K. Insights into symbiont population structure among three vestimentiferan tubeworm host species at eastern Pacific spreading centers. *Appl Environ Microbiol* 2016; **82**: 5197–5205.

44. Dahl C & Prange A. Bacterial sulfur globules: occurrence, structure and metabolism. In: Shively JM (eds). *Inclusions in prokaryotes*. Springer, Berlin, Heidelberg, 2006, pp 21–51.

45. Jonson AB, Normark S, Rhen M. Fimbriae, pili, flagella and bacterial virulence. *Contrib Microbiol* 2005; **12**: 67–89.

46. Nussbaumer AD, Fisher CR, Bright M. Horizontal endosymbiont transmission in hydrothermal vent tubeworms. *Nature* 2006; **441**: 345–348.

47. Lalioui L, Pellegrini E, Dramsi S, Baptista M, Bourgeois N, Doucet-Populaire F *et al*. The SrtA Sortase of *Streptococcus agalactiae* is required for cell wall anchoring of proteins containing the LPXTG motif, for adhesion to epithelial cells, and for colonization of the mouse intestine. *Infect Immun* 2005; **73**: 3342-3350.

48. Li Y, Liles MR, Halanych KM. Endosymbiont genomes yield clues of tubeworm success. *ISME J* 2018; **12**: 2785–2795.

49. Dziarski R, Gupta D. The peptidoglycan recognition proteins (PGRPs). *Genome Biology* 2006; **7**: 232.

50. Wippler J, Kleiner M, Lott C, Gruhl A, Abraham PE, Giannone RJ *et al*. Transcriptomic and proteomic insights into innate immunity and adaptations to a symbiotic lifestyle in the gutless marine worm *Olavius algarvensis*. BMC Genomics 2016; **17**: 942.

51. Vasta GR, Amzel LM, Bianchet MA, Cammarata M, Feng C, Saito K. F-type lectins: a highly diversified family of fucose-binding proteins with a unique sequence motif and structural fold, involved in self/non-self-recognition. *Front Immunol* 2017; **8**:1648.

52. Nyholm SV, Song PF, Dang JN, Bunce C, Girguis PR. Expression and putative function of innate immunity genes under in situ conditions in the symbiotic hydrothermal vent tubeworm *Ridgeia piscesae*. *Plos One* 2012; **7**: e38267.

53. Steindler L, Schuster S, Ilan M, Avni A, Cerrano C, Beer S. Differential gene expression in a marine sponge in relation to its symbiotic state. *Mar Biotechnol* 2007; **9**: 543–549.

Figure legends

**Supplementary Figure S1**: (**a**) A photograph of tubeworm *Paraescarpia echinospica* which stored in absolute ethanol. Scale bar = 5 cm. (**b**) A photograph showing where the tubeworms live *in situ*.

**Supplementary Figure S2**: (**a**) The differential coverage binning of the *Paraescarpia echinospica* and its symbiont (single species, SOB). Each circle represented a contig and its area indicated contig length, the color represented the taxonomy of the contig. The contigs of *P. echinospica* and its symbiont were grouped based on their different GC content and sequencing coverage. (**b**) The symbiont group is singled out and refined with principal component analysis (PCA) of tetranucleotide frequencies (TNFs).

**Supplementary Figure S3**: (**a**) Genome-similarity comparisons of symbionts from vent- and seep-living siboglinids constructed using BRIG. The innermost ring represents the reference sequence, the symbiont of *Paraescarpia*. The second and third rings show GC skew and GC content. The remaining rings represent the symbiont genomes from seep-living *Escarpia*, *Seepiophila*, *Lamellibrachia* and vent-dwelling *Riftia*, *Ridgeia*, *Tevnia*. The identifiers of inside-to-outside rings are listed on the right. (**b**) Synteny map of the symbionts of *Paraescarpia* and *Lamellibrachia*, sequences were placed around a circle, read clockwise, syntenic regions of each species are linked with ribbons. 2,283 local alignments were produced with 1 × 10^-10^ BLAST threshold, ribbons only created from 1,000 local alignments because Circoletto allowed up to 1,000 for clarity. The ribbons width the alignment length, the colors the alignment bitscore, our alignments produced min and max bitscore were 555.0 and 37670.0 respectively, and 'score/max' ratio was used coloring with blue <= 0.25, green <= 0.50, orange <= 0.75, red > 0.75.

**Supplementary Figure S4**: The maximum likelihood (ML) phylogenetic tree of vestimentiferan symbionts and *γ-Proteobacteria* symbionts of other marine invertebrates based on 16S rRNA gene. Numbers above branches represent ML bootstrap values based on 1,000 iterations, with 100 as the highest value. Nodes with bootstrap support > 70% were indicated. *δ-Proteobacteria* symbionts of genus *Olavius* were taken as outgroup, the symbiont of *Paraescarpia echinospica* was in blue color.

**Supplementary Figure S5**: (**a**) Gene Ontology classification of the *Paraescarpia* symbiont genome. The number and percent of genes in cellular components, molecular function and biological process ontology classiﬁcation group were generated by online tool WEGO, X-axis shows the default selected GO terms. (**b**) COG annotation of *Paraescarpia* symbiont genome, orange bars show the genes number and blue bars show the genes percentage.

**Supplementary Figure S6**: Phylogenomic analysis of bacterial symbionts (right side) and their associated siboglinid hosts (left side). Vent- and seep-living vestimentiferans are colored in red and blue respectively.

**Supplementary Figure S7**: (**a**) The correlation analysis of real-time PCR and proteome data based on 10 genes. X-axis shows the expression levels of protein sequences from proteome data using Log_10_ emPAI%, Y-axis shows the expression levels of genes from real-time PCR data using Log_10_ Fold-change. (**b**) The percentages of functional categories of the proteome and transcriptome of the *Paraescarpia* symbiont, total 425 proteins and 831 transcripts were annotated respectively with COG numbers against the COG database using BLASTp. (**c**) The 475 most highly expressed transcripts and proteins from the proteome and transcriptome analyses of the *Paraescarpia* symbiont were selected respectively to show the number of shared annotated proteins using Venn diagram. (**d**) The correlation analysis of transcriptome and proteome data based on 20 shared transcripts/proteins, which were randomly selected from the Venn diagram. X-axis shows the expression levels of protein sequences from proteome data using Log_10_ emPAI%, Y-axis shows the expression levels of transcripts from transcriptome data using Log_10_ TPM.

**Supplementary Figure S8**: Gene Ontology (GO) functional annotations of highly expressed transcripts in the plume, vestimentum and trophosome. X-axis represents the default GO terms selected automatically by WEGO online tool, the color of bars represents different regions of *Paraescarpia echinospica* (red - plume, purple - vestimentum, yellow - trophosome), the length of bars represents percentage and number of transcripts in different GO functional classes. GO annotation provides three main functional categories (cellular component, molecular function, and biological process), which include 13, 12, and 23 functional classes, respectively.

**Supplementary Figure S9**: Functional classification, class frequency and distribution of the highly expressed transcripts in the trophosome after annotation against the KOG database. KOG classifications were identified by letters of the alphabet and represented in different color bars, which categorized into four main categories (I. Information storage and Processing, II. Cellular processing and signaling, III. Metabolism and IV. Poorly characterized) according to their different functional classification.

**Supplementary Figure S10**: The correlations among significantly enriched GO terms of highly expressed genes in the trophosome (TPM ratio > 0.75) were visualized through GOEAST, enriched GO terms were classified into three GO categories: (a) biological process (b) cellular component and (c) molecular function. Bubbles represent GO terms, labeled by its term definition; bubble color indicates the p-value; bubble size indicates the frequency of the GO term in the underlying GOA database (a larger size indicated a more general term). Highly similar GO terms were linked by edges in the graph, where the line width indicates the degree of similarity. The initial placement of the nodes was determined by a ‘force-directed’ layout algorithm that aimed to keep the more similar nodes closer together.

**Supplementary Figure S11**: The maximum likelihood (ML) phylogenetic tree of potential Hb subunits sequences from Polychaeta marine worms. Hb subunit A1 of *Siboglinum fiordicum* served as outgroup, Hb sequences from *Paraescarpia echinospica* were highlighted in red color. Numbers above branches represent ML bootstrap values based on 1,000 iterations, with 100 as the highest value. Nodes with bootstrap support > 70% were indicated.

**Supplementary Figure S12**: (**a**) KEGG pathway of methionine biosynthesis in the endosymbionts of Siboglinidae. (**b**) KEGG pathways of methionine biosynthesis, blue and red color represented the genes which mapped to the pathway in the *Paraescarpia echinospica* host and its symbionts respectively.


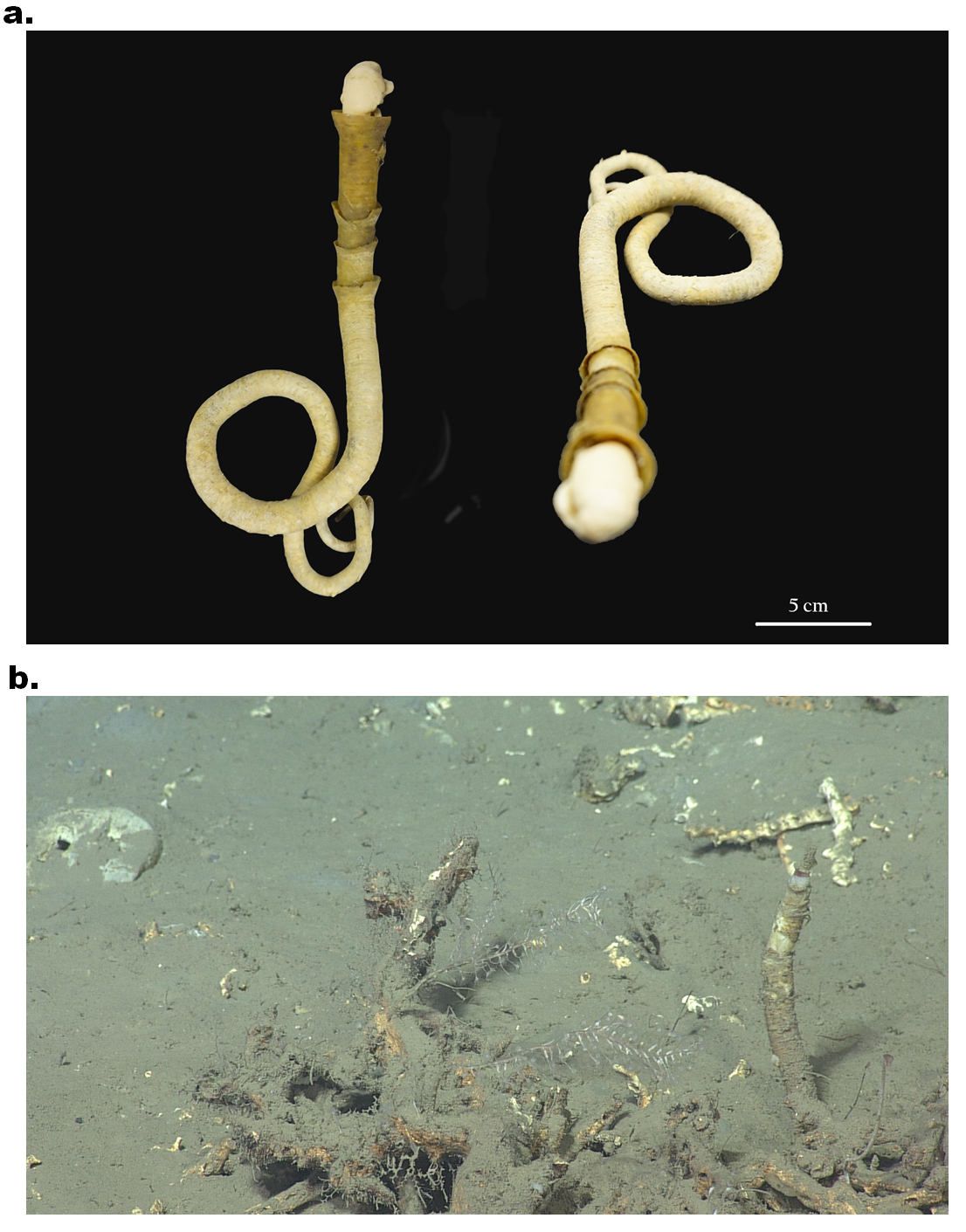


**Supplementary Figure S1**


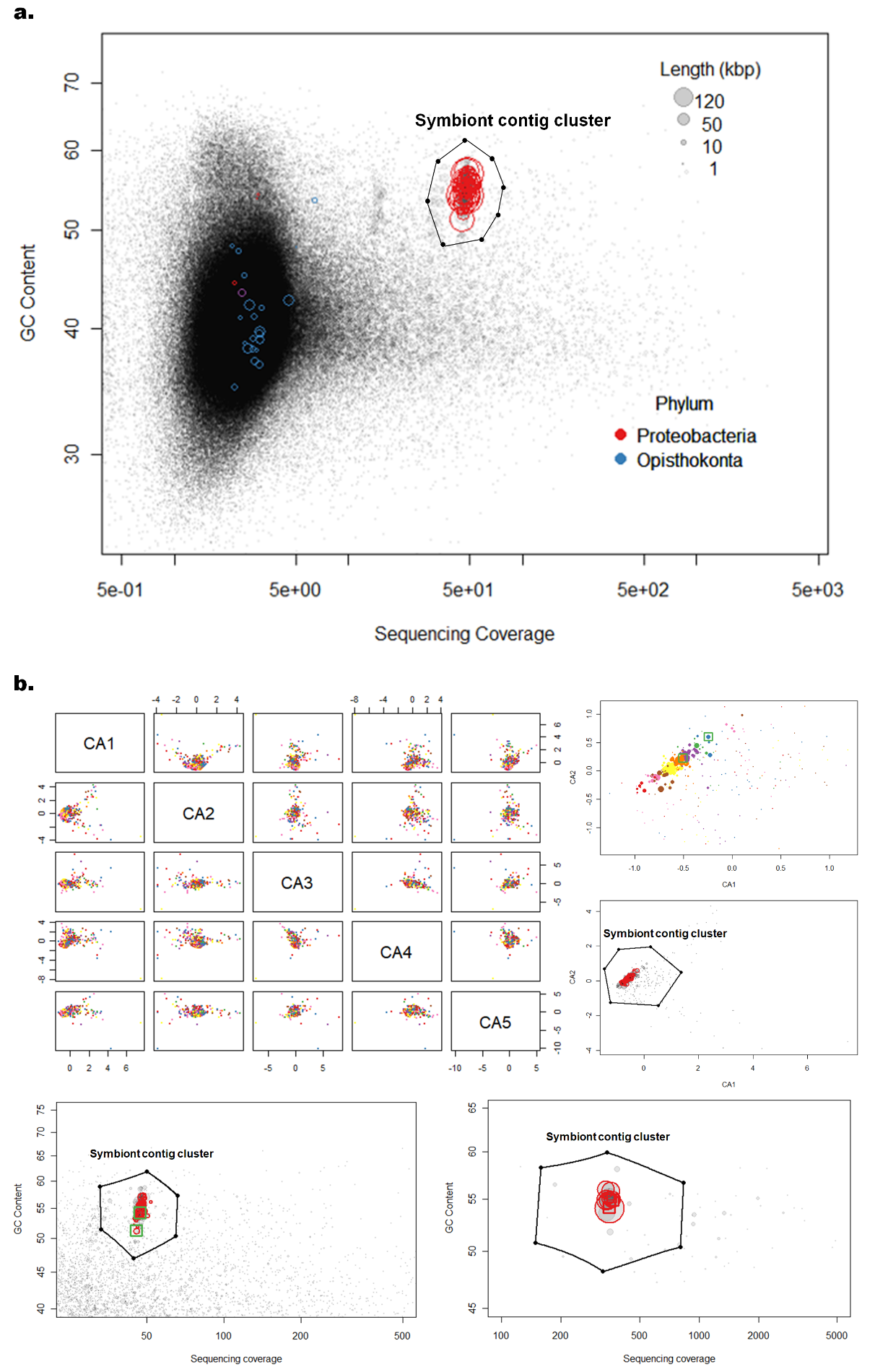


**Supplementary Figure S2**


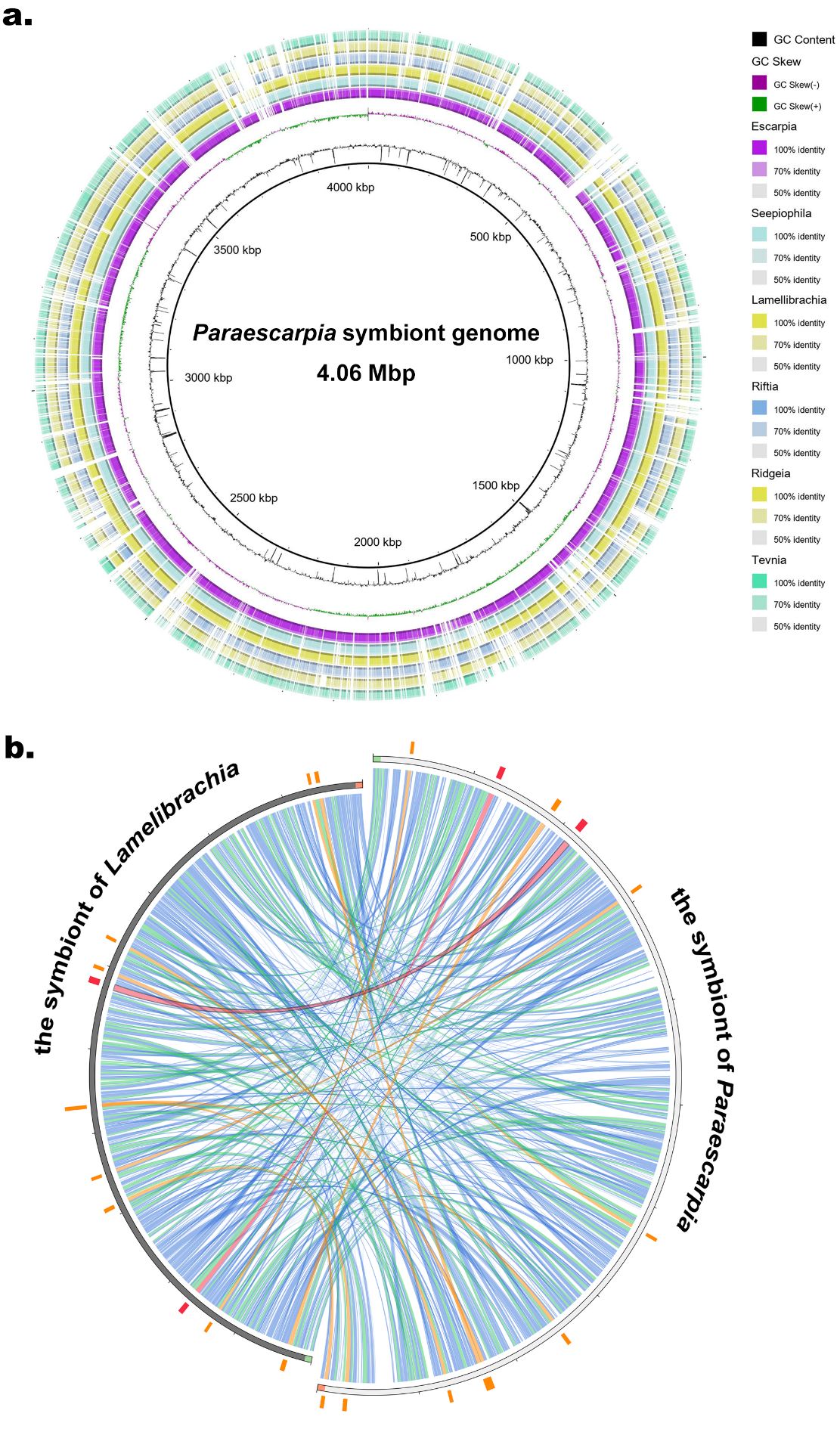


**Supplementary Figure S3**


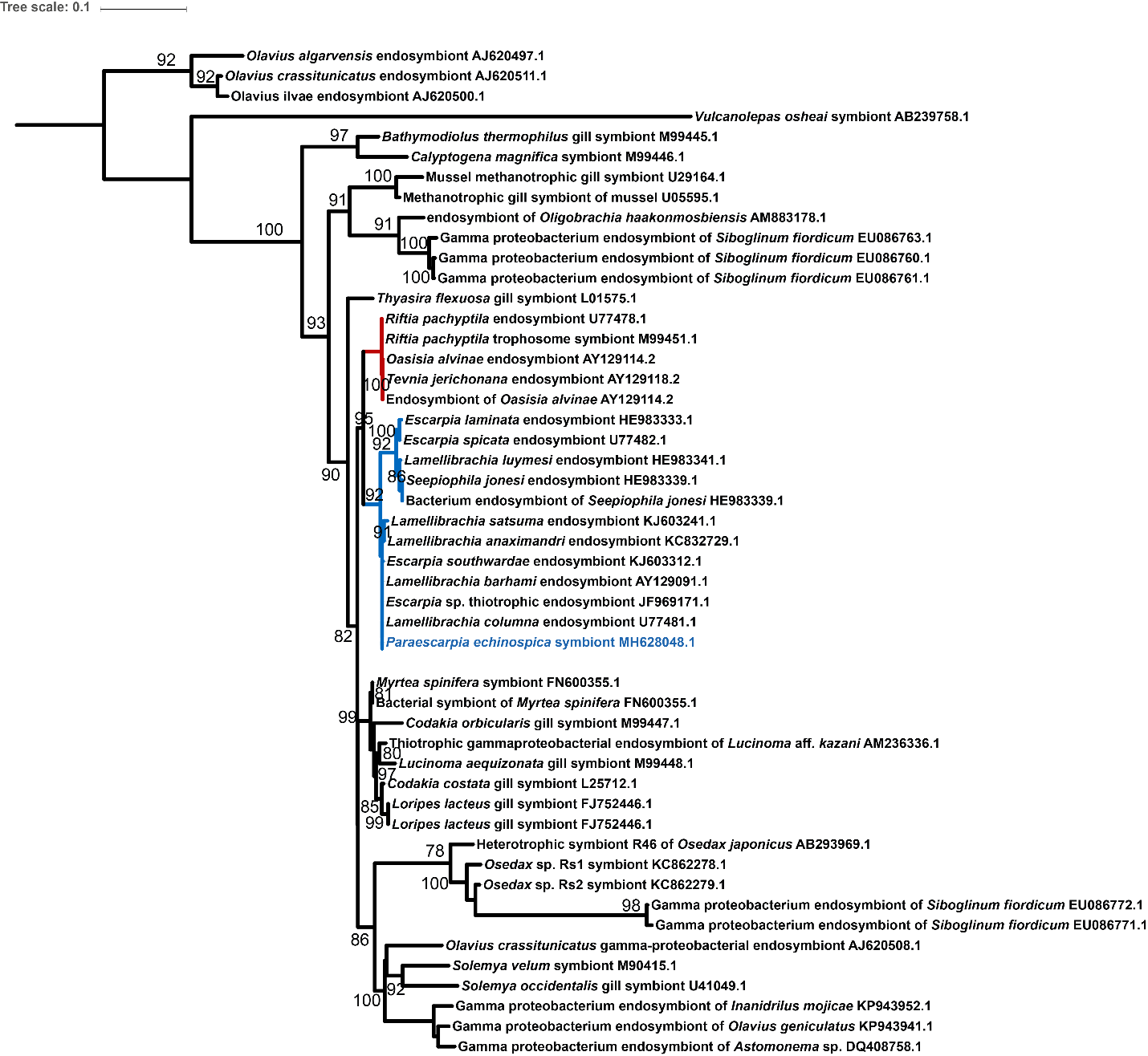


**Supplementary Figure S4**


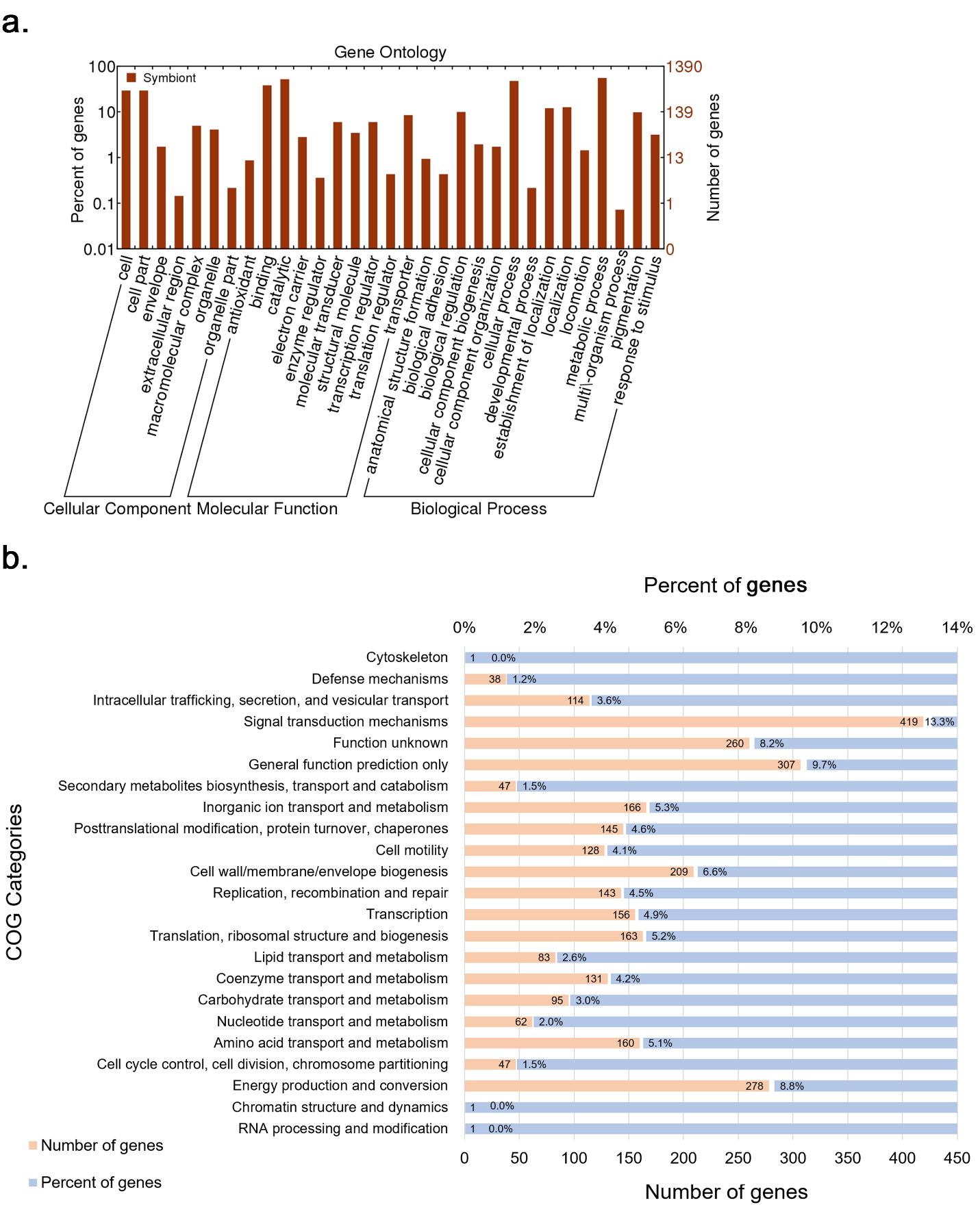


**Supplementary Figure S5**


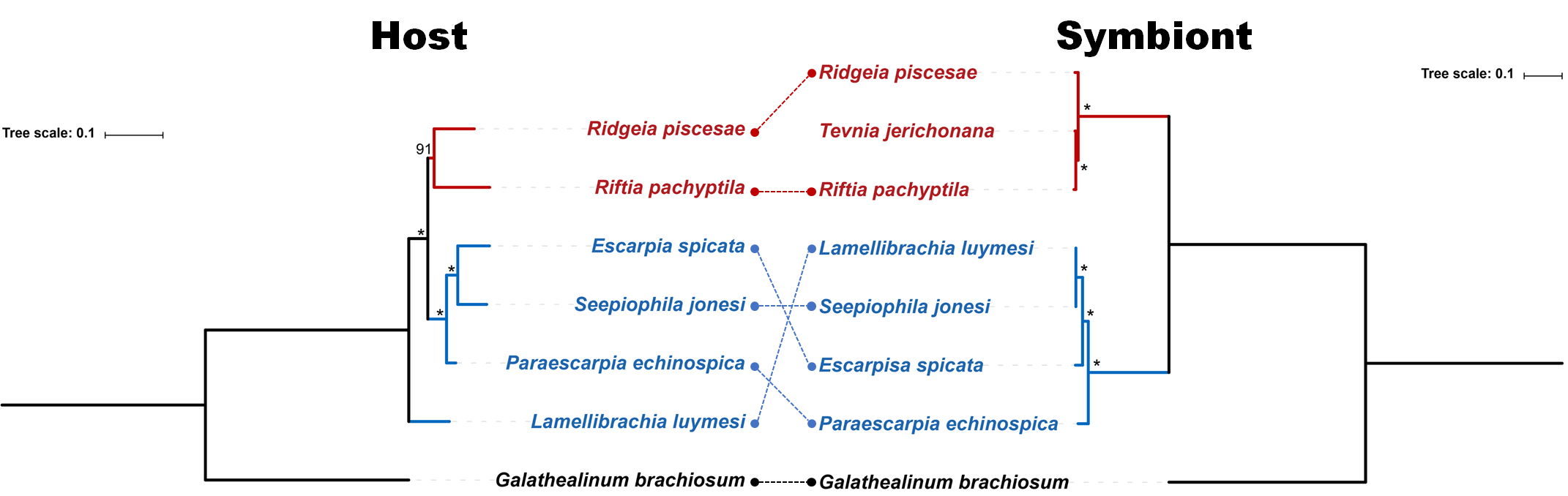


**Supplementary Figure S6**


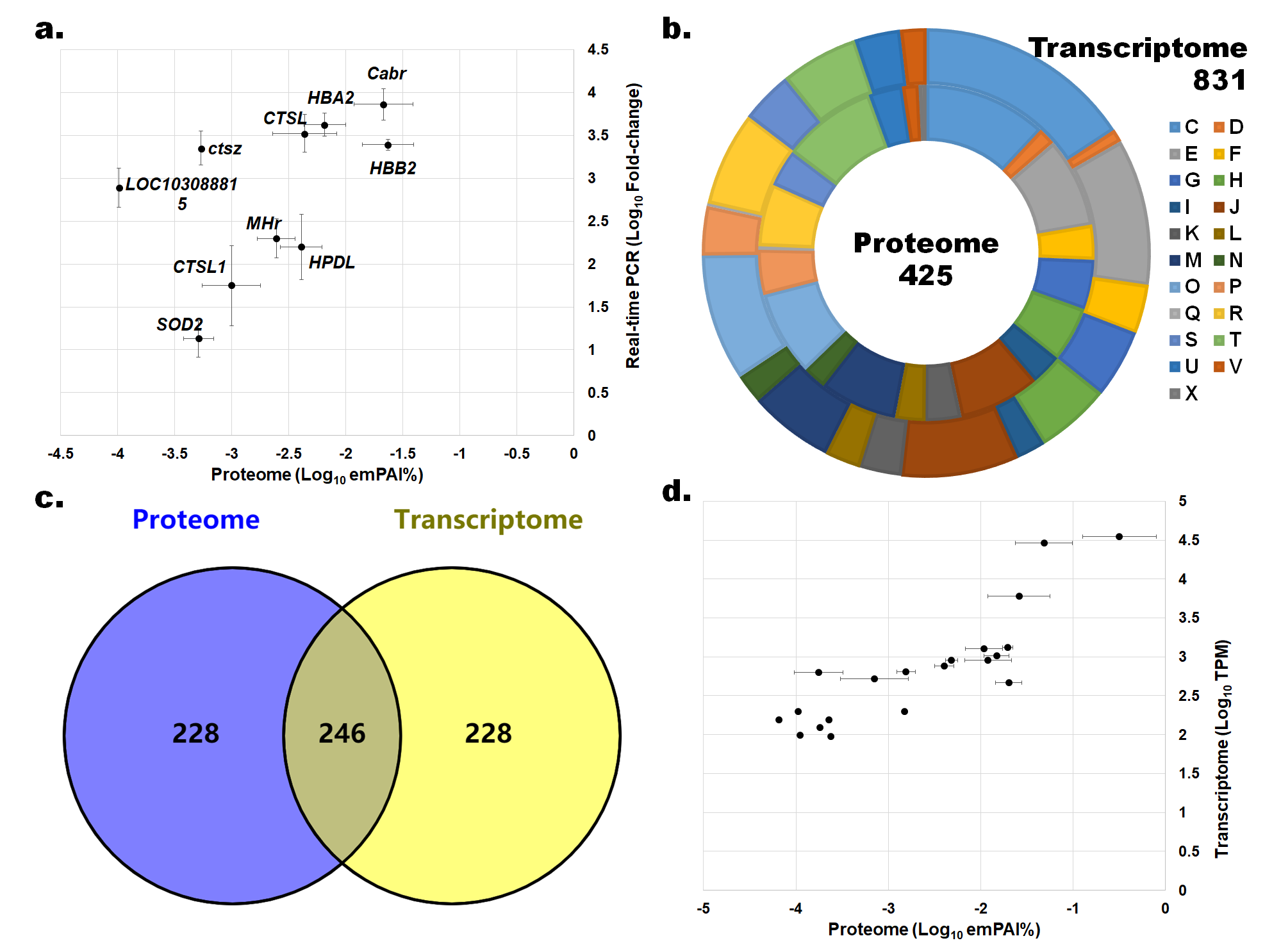


**Supplementary Figure S7**


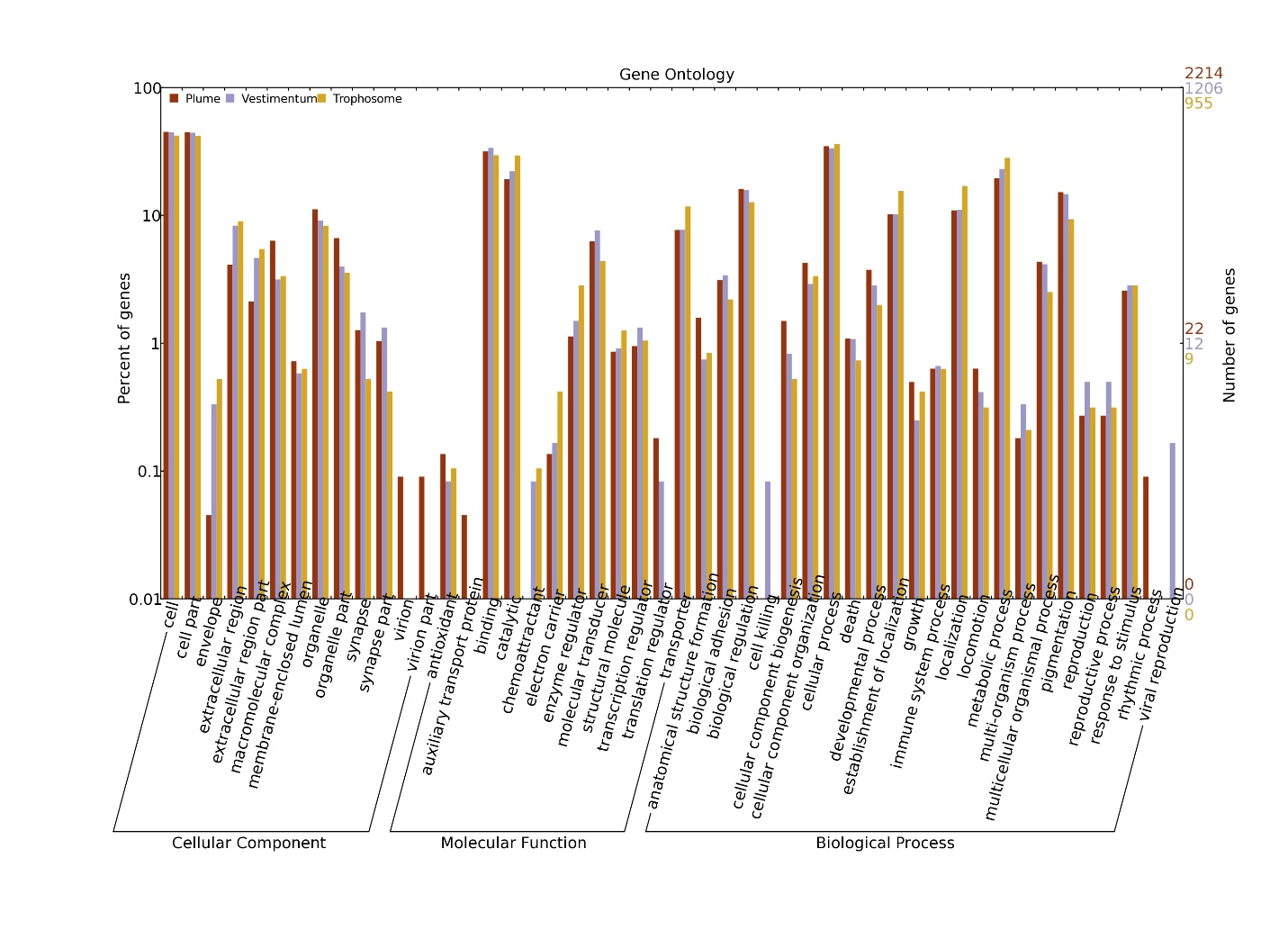


**Supplementary Figure S8**


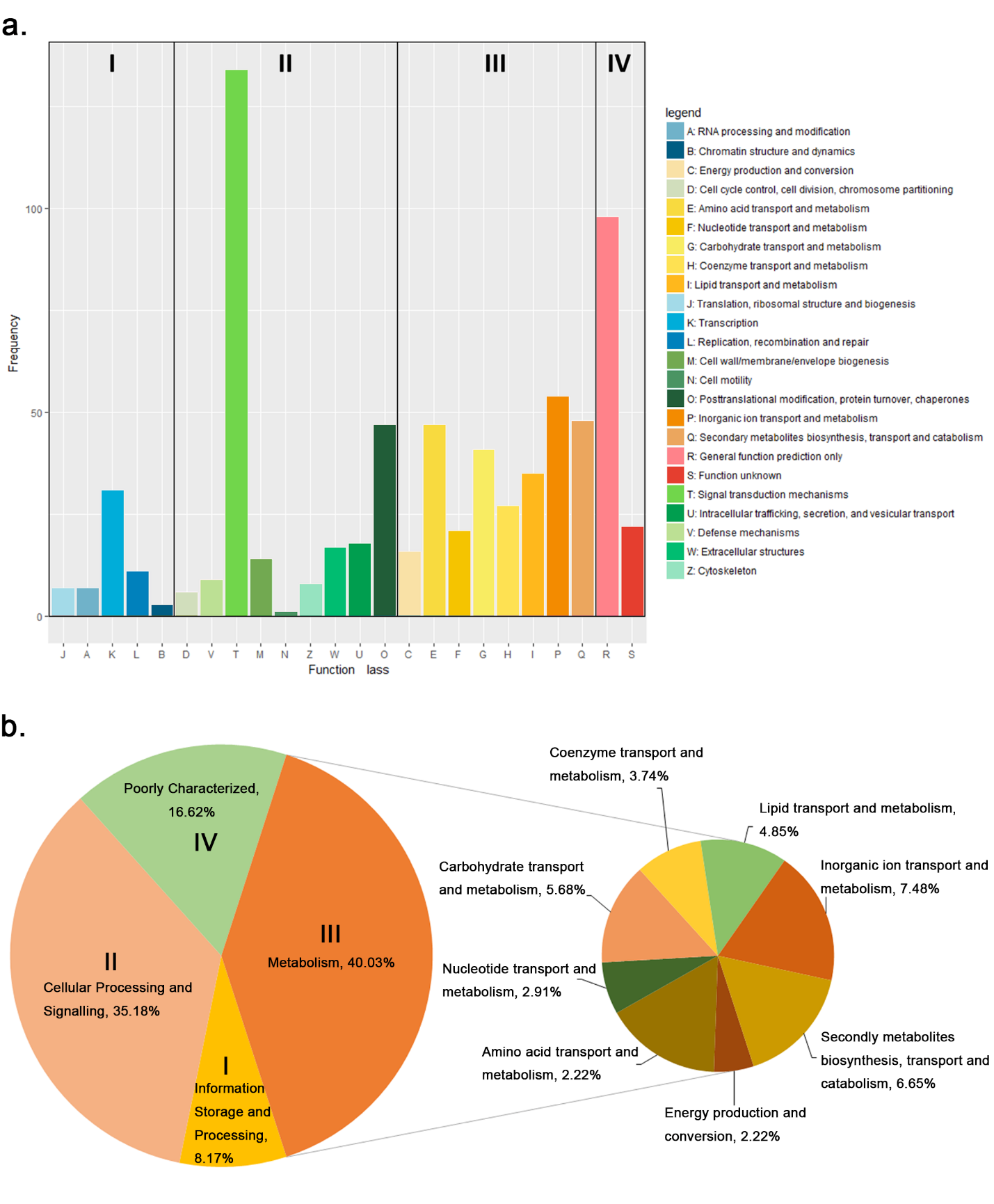


**Supplementary Figure S9**

**S10a.**


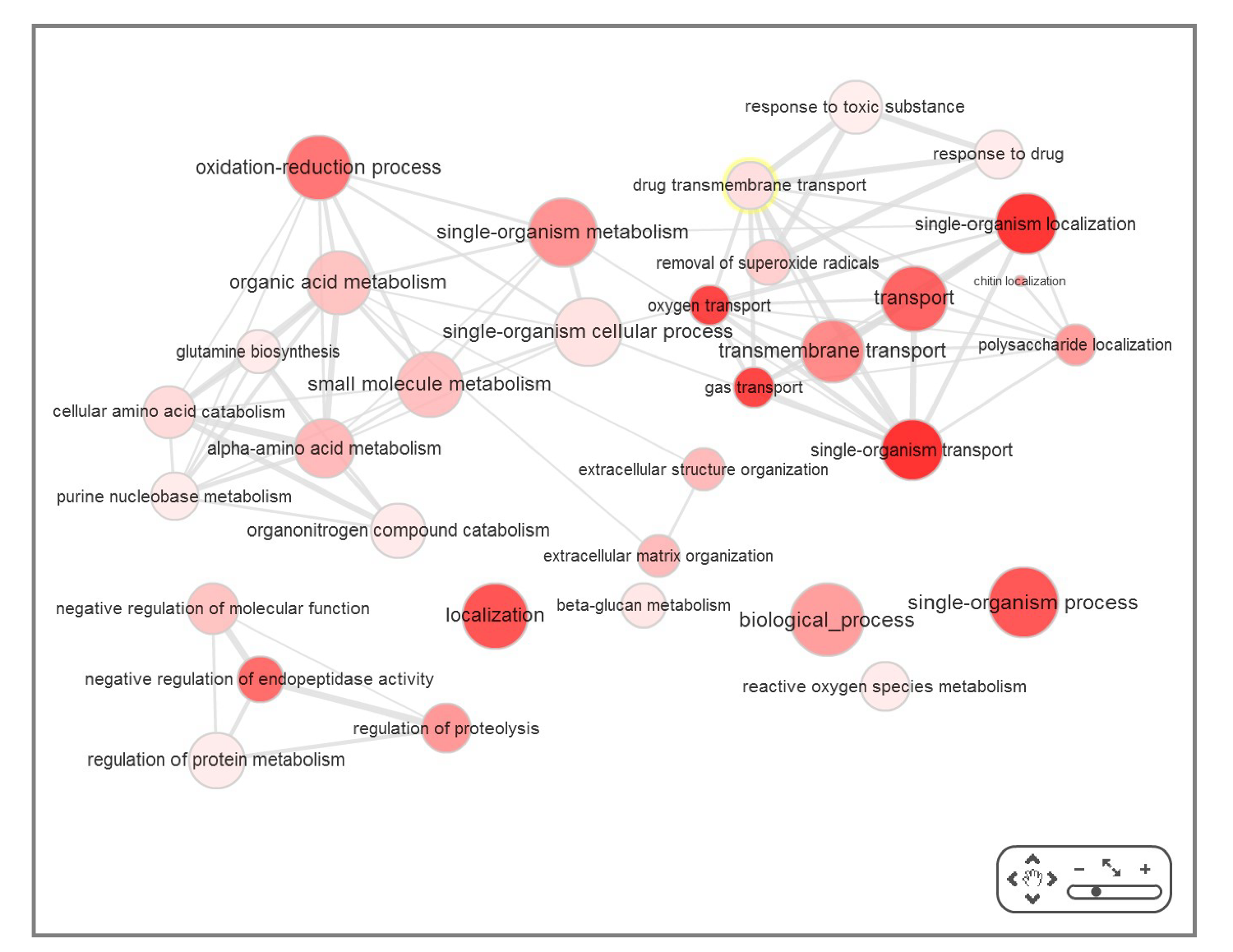


**S10b.**


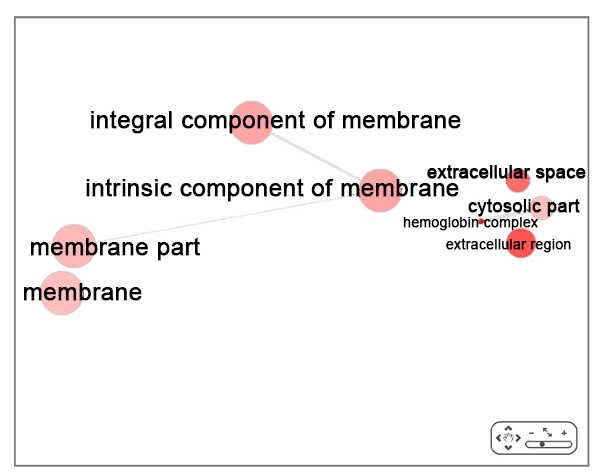


**S10c.**


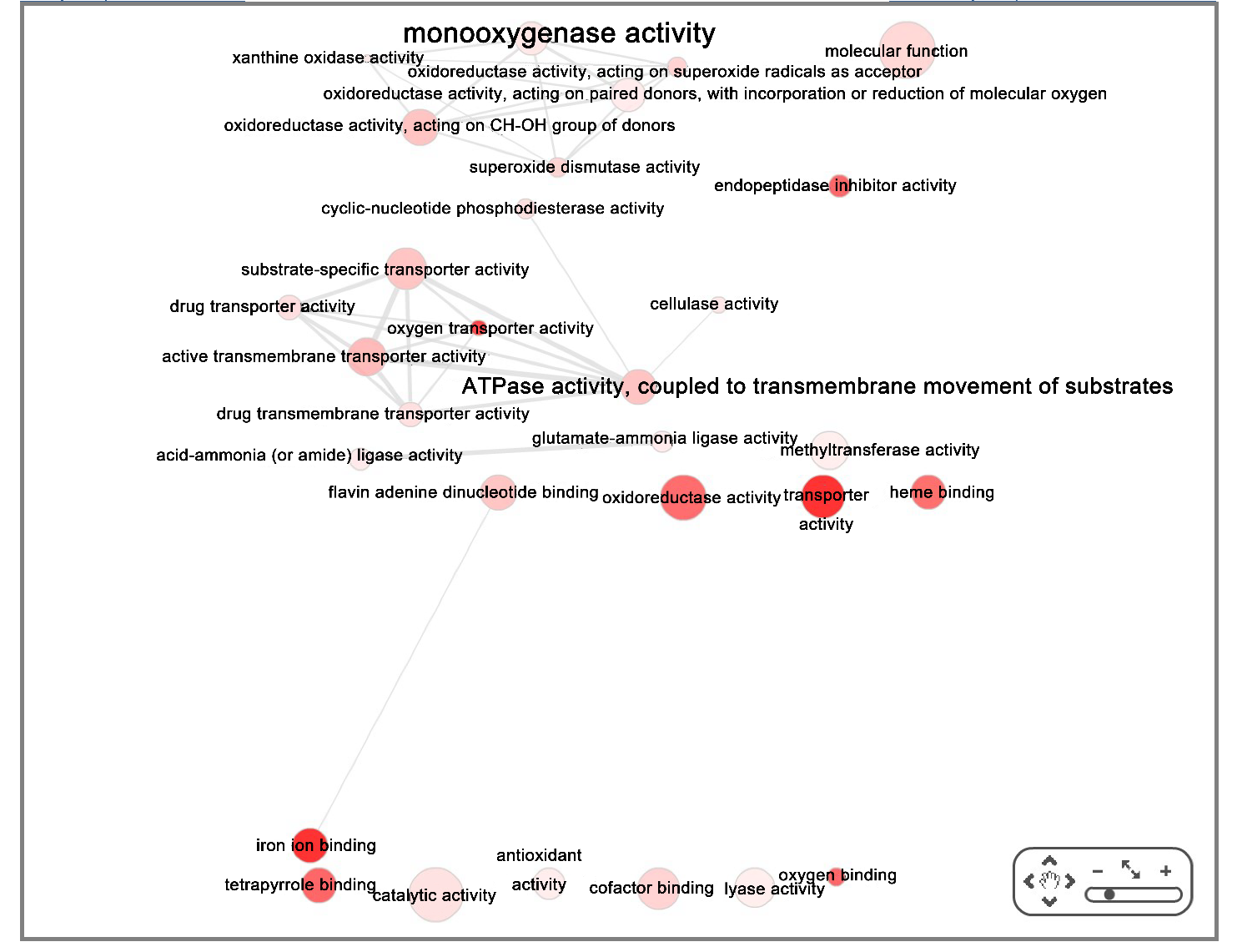


**Supplementary Figure S10**


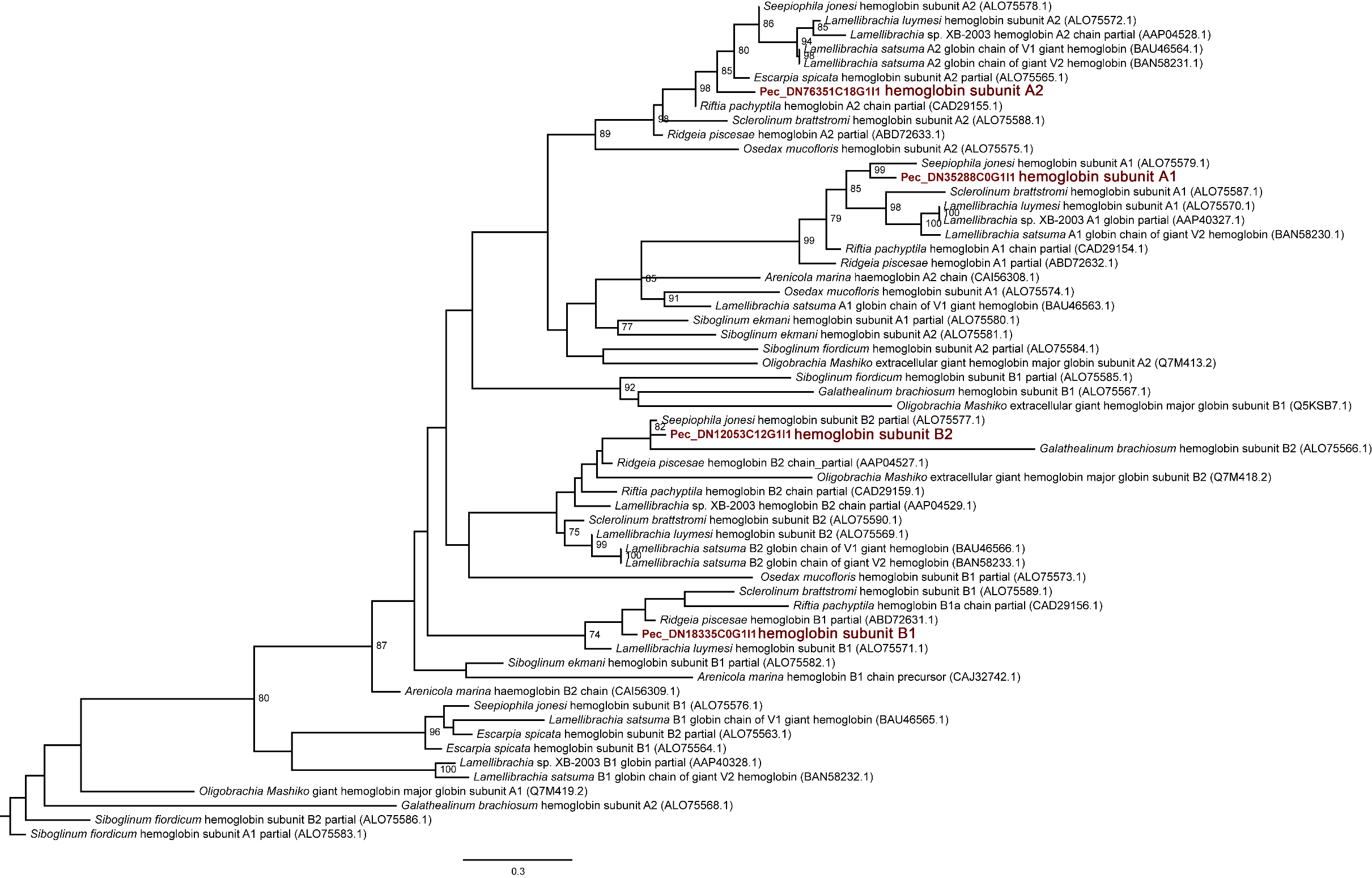


**Supplementary Figure S11**

**a**


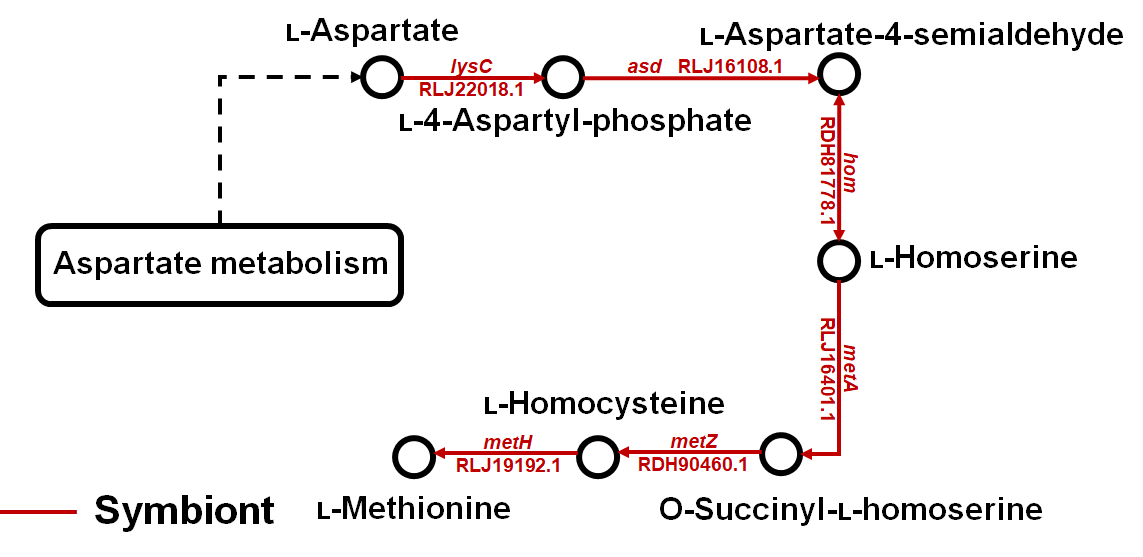


**b**


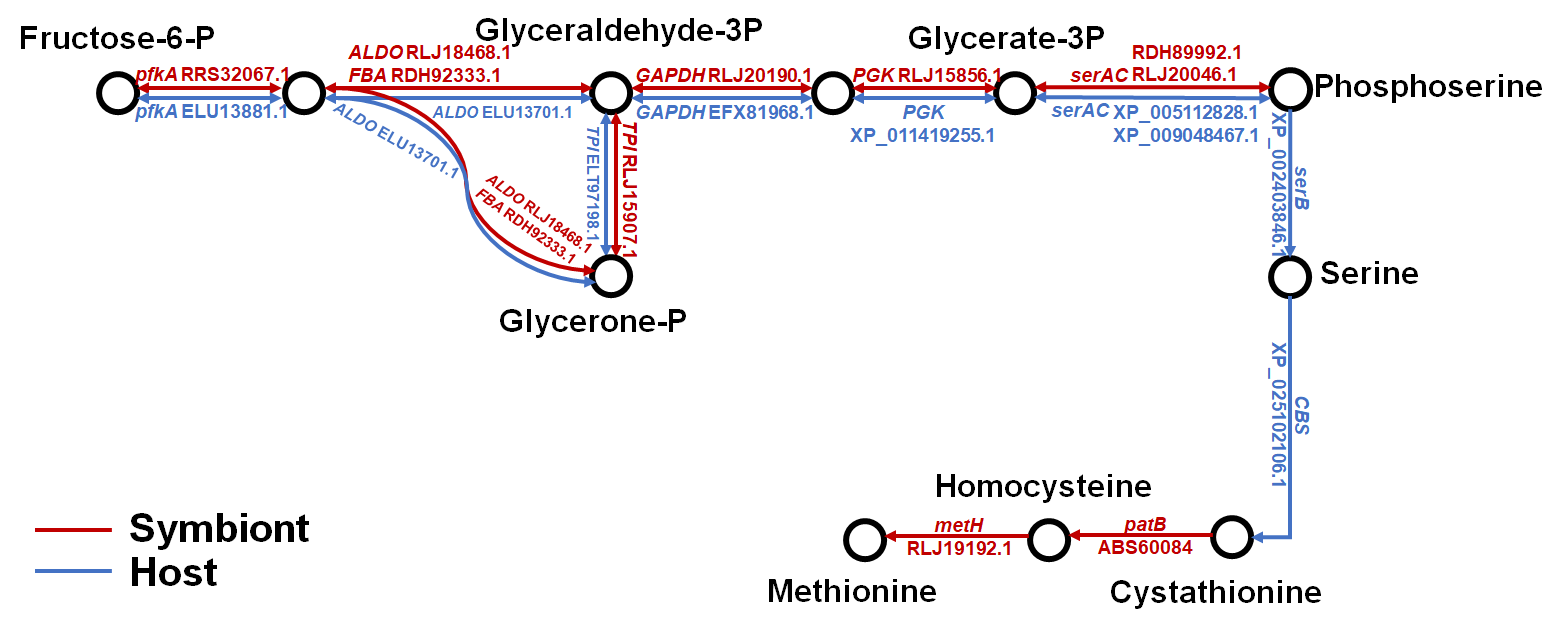


**Supplementary Figure S12**

**Table S1.** Source of transcriptome data used in phylogenomic analyses of siboglinid tubeworms.

| **Species** | **Data** | **Source** | **Accession number** | **Assembled Transcripts** | **Duplicates (%)** |
| --- | --- | --- | --- | --- | --- |
| *Riftia pachyptila* | 454 | NCBI SRA | SRR346550 | 6 042 | 0.7 |
| *Ridgeia piscesae* | 454 | NCBI SRA | SRR346554 | 10 973 | 1.0 |
| *Escarpia spicata* | 454 | NCBI SRA | SRR3554587 | 3 103 | 1.1 |
| *Lamellibrachia luymesi* | Illumina | NCBI SRA | SRR3556248 | 13 427 | 2.7 |
| *Seepiophila jonesi* | 454 | NCBI SRA | SRR3554599 | 4 064 | 1.0 |
| *Paraescarpia echinospica* | Illumina | This study | GHDM00000000 | 22 284 | 5.2 |
| *Galathealinum brachiosum* | 454 | NCBI SRA | SRR3664263.1 | 3 018 | 1.5 |

**Table S2.** Primers that were used for the real-time PCR analysis.

| **Gene ID** | **Annotation** | **Forward primer** | **Reverse primer** |
| --- | --- | --- | --- |
| Pec_DN842C0G1I1 | Eif3f | CAACACGTAACAACGGCCTG | GGTATCTGCCGGTCGTACTG |
| Pec_DN10730C0G1I1 | Cabr | CGAGTCTGTCACCTGGATCG | GACCCAGTGTTGGACGGTAG |
| Pec_DN12053C12G1I1 | HBB2 | GCGTGGCGGAGATGATTTTC | CCGATGGTCTTCCCGCATAA |
| Pec_DN76351C18G1I1 | HBA2 | TGGGGCACGACTTGTATTCC | ACGATGGGTATCGCCCTTTG |
| Pec_DN80961C0G1I1 | MHr | TTCGCCAAACAGTGGCTAGT | TCTCAGCGGAAGTGACGAAC |
| Pec_DN59547C0G1I1 | RHBG | TAAGGTGCTGTATCTGCCGC | TGTGATTTGTGGAGACCCCG |
| Pec_DN64251C0G1I1 | NKX | TCCAAAGTCACCGTCTTCCG | ATTACGACCGCACGTCACAT |
| Pec_DN69936C0G1I1 | SOD2 | AGTGATGTAGGGCTCGAGGT | ACTGGATCAACAGAGCCACG |
| Pec_DN67578C0G2I4 | CTSL | TCTGTCGGCATCTACGCATC | CAGTAGTCACGGCCCCTTTT |
| Pec_DN70089C0G4I1 | CTSL1 | TGGGGTAAGTCGTGGGGTAT | TCGTTCACACCAACGGGTAG |
| Pec_DN36980C0G1I1 | Neprilysin | TACTGGTTCCCTTCACGCAC | TTTTCAGGCGTATCAGGCGT |
| Pec_DN16800C0G1I1 | ctsz | CTCGTTGGGCTGACGTAGTT | TGACGAGAATGTGACCACGG |
| Pec_DN71728C4G1I3 | CBSL | CGTCATCTCAACGCCACTTG | GACCCCGTCAGTTCAGTTGT |
| Pec_DN41724C0G1I2 | ADI1 | TCGTCTACGCCATGCTTTGT | CTAACCGATTGACGGACCCC |
| Pec_DN71418C1G2I1 | HPDL | TGGTATTCTTCGGGTGGCAG | AAGCGTGTGAATTGTGTCGC |
| Pec_DN73628C1G3I1 | TLR6 | CACAAGGTGGCAAACCGATG | CGAGCTACCACCCTTCAGTC |
| Pec_DN66669C0G1I1 | TLR4 | TGTGATTCCTGCTCGTCGTT | GCACACGACTCCTCCAGATT |
| Pec_DN73715C2G2I1 | TLR2 | AGAATACTGCCTTTCCGGGC | AGACCACCAGTGCTTGCTTT |
| Pec_DN69820C0G1I1 | BPI | TTCCAGAGTTGTCTCGGTGC | CGTAAAAGCTCGCCACGATG |

**Table S3** Summary of assembly and functional annotation of the *Paraescarpia echinospica* symbiont genome

| Data produced from the two platforms | Illumina | Nanopore |
| --- | --- | --- |
| Total base (bp) | 35 289 062 700 | 2 413 233 679 |
| Total number of reads | 235 260 418 | 1 158 101 |
| Read length (bp) | 150 | 499 – 119 416 |
| Mean length of reads (bp) | - | 2 084 |
| N50 (bp) | - | 3 272 |
| Contigs of the host and its symbiont | **De novo assembly by SPAdes** | |
| Total base (bp) | 862 790 044 | |
| Total number of contigs | 627 097 | |
| Mean length of assembled contigs (bp) | 1 376 | |
| N50 (bp) | 3 136 | |
| Assembled contigs size range (bp) | 128 – 111 956 | |
| Scaffolds of the symbiont | **Scaffolding by SSPACE** | |
| Total base (bp) | 4 064 761 | |
| Total number of scaffolds | 14 | |
| Mean length of assembled scaffolds (bp) | 290 340 | |
| N50 (bp) | 381 676 | |
| Assembled scaffolds size range (bp) | 16 442 – 942 635 | |
| Functional annotation |  | |
| Coding sequence prediction |  | |
| No. of CDS predicted from Prodigal | 3 525 | |
| Mean length of CDS (aa) | 337 | |
| Total number of CDS annotated by public databases  (e-value < 1 e^-05^) | 2 906 | |
| COG | 2 577 | |
| KEGG | 2 523 | |
| Pfam | 2 808 | |
| Gene Ontology | 1 390 | |

**Table S6** Summary of assembly and functional annotation of the holobiont meta-transcriptome and the host transcriptome

| De novo assembly by Trinity | Holobiont meta-transcriptome | | Host transcriptome |
| --- | --- | --- | --- |
| Total base (bp) | 111 736 578 | | 95 587 053 |
| Total number of transcripts | 142 750 | | 118 820 |
| Mean length of transcripts (bp) | 783 | | 804 |
| N50 (bp) | 953 | | 1 015 |
| Assembled scaffolds size range (bp) | 301 – 33 918 | | 301 – 33 899 |
| Completeness | 97.6% | | 97.7% |
| Functional annotation |  | |  |
| Coding regions predicted from TransDecoder | 23 810 | | 22 284 |
| Mean length of CDS (aa) | 362 | | 366 |
| Total No. of CDS annotated by public databases (e-value < 1 e^-05^) | 19 655 | | 20 733 |
| Databases | **Host** | **Symbiont** | **Host** |
| NCBI non-redundant (nr) database | 17 879 | 1 087 | 18 865 |
| KOG | - | - | 12 971 |
| KEGG | 5 234 | 798 | 6 729 |
| Gene Ontology | 10 076 | 1 005 | 11 596 |
| COG | - | 880 | - |

**Table S7-1** The 50 most highly expressed genes of the *Paraescarpia echinospica* as identified in the meta-transcriptome analysis. The transcript sequence names and their annotated accession numbers and protein abbreviations were listed on the left, these proteins were classified into different functional categories (represented by different capital letters) based on KOG database, their expression levels in the three different regions of *Paraescarpia echinospica* were represented by TPM values.

| **Sequence Name** | **Accession number** | **Abbreviations** | **Description** | **Class** | **TPM value** | | |
| --- | --- | --- | --- | --- | --- | --- | --- |
|  |  |  |  |  | **Trophosome** | **Plume** | **Vestimentum** |
| Pec_DN57842C0G1I1 | XP_009014896.1 | RPL4 | typical for genes encoding ribosomal proteins | A | 26346 | 35536.2 | 10209.3 |
| Pec_DN82282C0G1I1 | ELT89952.1 | CAPTEDRAFT_198366 | hypothetical protein | S | 25804.5 | 5.72096 | 17.9235 |
| Pec_DN122777C0G1I1 | CAP08293.1 | MHr | responsible for oxygen transport | C | 22483.1 | 12548.8 | 22017.3 |
| Pec_DN85360C1G2I1 | XP_023931316.1 | ACTP | involved in regulation of actin polymerization | Z | 19601 | 14677.1 | 11720.5 |
| Pec_DN85360C1G1I3 | XP_013309372.1 | ACTG1 | involved in various types of cell motility | Z | 17693 | 14492.9 | 16338.5 |
| Pec_DN88597C1G1I5 | ELT96969.1 | PLC | cleave phospholipids | I | 16602.3 | 75.3679 | 45.6922 |
| Pec_DN76666C0G2I1 | XP_013401274.1 | SHLC | essential for ATPase activity, and the regulatory light chain | Z | 16029.7 | 474.203 | 37018 |
| Pec_DN80054C0G1I1 | ELT96141.1 | AMPH | the recruitment of dynamin to sites of clathrin-mediated endocytosis | U | 13422.2 | 1514.13 | 4128.74 |
| Pec_DN74409C0G2I1 | AHX26698.1 | MhmA | actin filament binding | Z | 11915.7 | 1158.74 | 15578.3 |
| Pec_DN28928C0G1I1 | AAG16892.1 | MrlC | regulation of both smooth muscle and nonmuscle cell contractile activity | Z | 10096.9 | 1763.05 | 21483.2 |
| Pec_DN19286C0G1I1 | ELT90173.1 | AFP | actin lateral binding | Z | 7354.02 | 1488.98 | 8637.57 |
| Pec_DN88252C4G1I1 | CBM42049.1 | HSP70 | protein folding and the response to stress | O | 6605.12 | 2855.95 | 4878.01 |
| Pec_DN76686C0G1I1 | P04572.1 | SCP | participate in calcium cell signalling pathways | C | 6280.35 | 6.80394 | 12120.5 |
| Pec_DN75747C0G1I1 | BBB04239.1 | PMLI | a major structural component of thick filaments | Z | 5685.99 | 1961.57 | 5863.98 |
| Pec_DN90435C0G1I1 | ELU15936.1 | CAPTEDRAFT_199465 | hypothetical protein | S | 4901.59 | 2.39328 | 18.7265 |
| Pec_DN62609C3G1I1 | ELU06642.1 | Tnni | a cardiac and skeletal muscle protein | Z | 4639.03 | 703.227 | 8305.59 |
| Pec_DN83056C1G1I4 | XP_024351870.1 | VprBP | immunodeficiency virus gene and protein product | D | 4511.25 | 48.7631 | 2763.8 |
| Pec_DN78044C0G1I2 | ELU00393.1 | SM20 | calcium ion binding | T | 4492.96 | 707.995 | 8229.63 |
| Pec_DN41355C0G1I1 | AOR07117.1 | TEF1 | responsible for the GTP-dependent binding of aminoacyl-tRNAs to ribosomes | J | 4339.82 | 6652.46 | 5877.03 |
| Pec_DN66262C0G1I1 | XP_009011247.1 | TnT | a part of the troponin complex | Z | 4286.92 | 1445.03 | 17559.4 |
| Pec_DN90207C0G2I6 | XP_025104157.1 | NCAM-L1 | cell adhesion and transmembrane signals | T | 4233.98 | 18.1898 | 300.679 |
| Pec_DN102080C0G1I1 | XP_014237205.1 | RPS8 | catalyze protein synthesis | J | 3758.83 | 4565.05 | 4026.57 |
| Pec_DN81629C0G1I3 | XP_007668047.1 | SPINT | inhibit the function of protein degrading enzymes | O | 3466.49 | 1583.16 | 3852.57 |
| Pec_DN88252C2G1I2 | CAB38180.1 | LaminIF | structural molecule activity | DY | 3375.32 | 5774.25 | 2752.3 |
| Pec_DN67653C0G1I1 | XP_013406659.1 | TF_bZIP | cell proliferation, differentiation and survival | K | 3125.95 | 600.132 | 2594.78 |
| Pec_DN90784C2G1I1 | ABX44845.1 | CEP52 | Activating enzyme | J | 3065.77 | 6309.44 | 4742.28 |
| Pec_DN81937C1G1I1 | ABD94500.1 | TUBA | the major constituent of microtubules | Z | 2874.86 | 10657.2 | 2873.71 |
| Pec_DN70102C0G1I1 | XP_012942981.1 | MyoL | Immunoreactivity | Z | 2754.55 | 880.773 | 6589.76 |
| Pec_DN37219C0G1I1 | ABW23155.1 | RpS27A | Activating enzyme | J | 2661.28 | 5384.11 | 4261.12 |
| Pec_DN77031C0G1I1 | ALO75576.1 | HBB1 | Oxygen transport | C | 2583.18 | 4.47726 | 10.5249 |
| Pec_DN90864C0G1I1 | BAU46563.1 | HBA1 | Oxygen transport | C | 2432.03 | 4.60245 | 8.08868 |
| Pec_DN34473C0G1I1 | BAE93065.1 | GNB2L1 | the recruitment, assembly and regulation of signaling molecules | T | 2122.09 | 4539.94 | 2931.29 |
| Pec_DN80452C0G1I1 | XP_023933375.1 | Staufen | RNA-binding protein | UK | 2000.95 | 436.035 | 1562.34 |
| Pec_DN79291C0G1I1 | ELU06754.1 | Transgelin | Involved in calcium interactions and contractile properties of the cell | Z | 1995.6 | 200.175 | 3655.64 |
| Pec_DN85619C1G1I1 | ELU15842.1 | PEPCK | conversion of oxaloacetate to phosphoenolpyruvate | C | 1944.44 | 362.663 | 4760.05 |
| Pec_DN19336C0G1I1 | ELU06544.1 | CNN | a basic smooth muscle protein | Z | 1933.15 | 638.78 | 4893.54 |
| Pec_DN43889C0G2I1 | ADW95789.1 | RpS30 | Activating enzyme | JO | 1684.93 | 3436.5 | 2272.26 |
| Pec_DN40178C0G1I1 | ALO75578.1 | HBA2 | Oxygen transport | C | 1682.15 | 25.2945 | 5.46307 |
| Pec_DN72307C1G1I1 | ABO26619.1 | 67LR | extracellular matrix glycoproteins | J | 1669.07 | 4311.01 | 2807.58 |
| Pec_DN58083C0G1I1 | NP_001268726.1 | APJUN | cell proliferation, differentiation and survival | K | 1548.75 | 699.842 | 1458.13 |
| Pec_DN96948C0G1I1 | WP_087554851.1 | MCHLO_11918 | actin binding | TZ | 1490.57 | 1.01109 | 2.23753 |
| Pec_DN85396C3G3I1 | XP_792246.3 | DHX9 | involving alteration of RNA secondary structure | A | 1455.97 | 873.116 | 1142.65 |
| Pec_DN88760C0G2I2 | XP_021378624.1 | JmjC | transcriptional repression and chromatin regulation | R | 1446.99 | 1311.81 | 1716.73 |
| Pec_DN84729C0G2I1 | ELU15564.1 | CTF1 | transferase activity, transferring glycosyl groups | GO | 1440.29 | 0.242511 | 52.2619 |
| Pec_DN38213C0G1I1 | O18477.1 | TCTP | calcium binding and microtubule stabilization | DZ | 1395.18 | 2344.71 | 2875.58 |
| Pec_DN24444C0G1I1 | XP_009022669.1 | EF-2 | an essential factor for protein synthesis. | J | 1391.98 | 1989.17 | 1274.07 |
| Pec_DN12043C0G1I1 | ABW24383.1 | Cox6c2 | mitochondrial electron transport | C | 1379.33 | 2204.26 | 2427.78 |
| Pec_DN88072C5G2I2 | XP_008522290.1 | MS4A4A | involved in signal transduction | T | 1355.7 | 2463.88 | 1086.49 |
| Pec_DN12426C0G1I1 | XP_011450032.1 | NACTSN | calcium-transporting ATPase activity | K | 1339.51 | 1659.06 | 1273.37 |
| Pec_DN85904C3G1I3 | XP_013399449.1 | MRM | translation of mitochondrial-derived transcripts | A | 1285.2 | 358.282 | 605.832 |

**Table S7-2** The 50 most highly expressed genes of the symbiont of *Paraescarpia echinospica* as identified in the meta-transcriptome analysis. The transcript sequence names and their annotated accession numbers and protein abbreviations were listed on the left, these proteins were classified into different functional categories (represented by different capital letters) based on COG database, their expression levels in the symbiont were represented by TPM values.

| **Sequence Name** | **Accession number** | **Abbreviations** | **Description** | **Class** | **TPM** |
| --- | --- | --- | --- | --- | --- |
| Pec_DN78589C0G1I1 | PIK61579.1 | PSE | hydrolase activity | C | 48259.8 |
| Pec_DN83696C0G1I2 | RDH89092.1 | N2OR | bacterial denitrification | C | 39765.2 |
| Pec_DN96205C0G1I1 | RLJ19271.1 | RuBisCO | carbon fixation | G | 34956.7 |
| Pec_DN82760C0G1I2 | RDH86736.1 | TC.GBP | bacterial pathogenesis | M | 28997.2 |
| Pec_DN59899C0G1I1 | WP_069127019.1 | Sgp | sulfurstorage | C | 9328.84 |
| Pec_DN124220C0G1I1 | RLJ16414.1 | Tscrpt_reg_Rrf2 | electron transport from hydrogen to sulphate | K | 8580.25 |
| Pec_DN76711C0G1I1 | RLJ17986.1 | APSR | an enzyme that catalyzes sulfite and glutathione disulfide | C | 6074.35 |
| Pec_DN65699C0G1I1 | RDH91293.1 | CYTC | a formate dehydrogenase | C | 4317.64 |
| Pec_DN19511C0G1I1 | WP_027336974.1 | Group_II_RT_mat | a reverse transcriptase and splicing factor | L | 2514.53 |
| Pec_DN87004C0G1I1 | RLJ15739.1 | DsrAB | dissimilatory sulphate reduction | C | 2444.63 |
| Pec_DN86791C0G1I1 | WP_005966429.1 | H+, Na+-PPase | generating a proton motive force | C | 2427.17 |
| Pec_DN90200C0G1I2 | RLJ16596.1 | IDH | catalyzes the oxidative decarboxylation reaction | C | 1656.22 |
| Pec_DN83799C0G1I3 | RDH90932.1 | IS1595 | transposase | C | 1525.15 |
| Pec_DN81085C0G1I1 | RLJ17628.1 | HSP60 | protein folding | O | 1324.88 |
| Pec_DN90200C0G3I1 | RDH88904.1 | Succ_Dhase_FrdA_Gneg | anaerobic respiration | C | 1263.77 |
| Pec_DN84336C0G1I1 | RLJ21283.1 | Cbb3-Cox subunit I | aerobic respiration | O | 1253.97 |
| Pec_DN86791C0G2I1 | RLJ20164.1 | ATP-PFK | glycolysis | G | 1040.48 |
| Pec_DN88182C3G2I1 | BBG29096.1 | Orf91 | DNA integration | L | 996.175 |
| Pec_DN33952C0G1I1 | RDH90312.1 | FP2_01370 | peptidase activity | O | 908.838 |
| Pec_DN88182C3G3I1 | RLJ18725.1 | RplB | 50S peptidyl transferase activity | J | 908.257 |
| Pec_DN824C0G1I1 | RDH82198.1 | GGT | catalyzes the transfer of gamma-glutamyl functional groups from molecules | E | 769.21 |
| Pec_DN83546C1G2I1 | RDH82011.1 | CYCS | heme protein | R | 746.894 |
| Pec_DN90200C0G2I1 | RLJ16597.1 | SCS | citric acid cycle | C | 739.541 |
| Pec_DN19320C0G1I1 | RDH90713.1 | RplS | ribosomal large subunit assembly | J | 696.141 |
| Pec_DN53753C0G1I1 | RDH89003.1 | HSP20 | protect other proteins against heat-induced denaturation and aggregation | O | 684.954 |
| Pec_DN116837C0G1I1 | RLJ16156.1 | ASK | catalyzes the phosphorylation of the amino acid aspartate | E | 667.412 |
| Pec_DN102842C0G1I1 | RLJ20034.1 | FstH | integral component of membrane | R | 657.79 |
| Pec_DN87004C0G4I1 | RDH88731.1 | RTO_06290 | glutamate synthase (NADH) activity | ER | 648.058 |
| Pec_DN66909C0G1I1 | RLJ15866.1 | Sox | sulfur oxidation | S | 646.106 |
| Pec_DN110256C0G1I1 | RLJ15835.1 | DTD | deacylate D-aminoacyl-tRNAs into free D-amino acids | J | 645.783 |
| Pec_DN88182C3G1I1 | RDH85545.1 | CpSecY | the protein translocation channel | U | 632.02 |
| Pec_DN65198C0G1I1 | RLJ19091.1 | ATP-synt | catalyzes the synthesis of ATP from ADP | C | 627.286 |
| Pec_DN76934C0G1I1 | RLJ18187.1 | NR | catalyze the reduction of nitrite | C | 625.539 |
| Pec_DN53418C0G1I1 | RLJ20930.1 | CCP | eliminate toxic radical molecules | P | 615.783 |
| Pec_DN46352C0G1I1 | RDH85354.1 | HyuB | Arginine and proline metabolism | EQ | 581.138 |
| Pec_DN1813C0G1I1 | RDH82213.1 | RpoD | promote the attachment of RNA polymerase to specific initiation sites | K | 555.616 |
| Pec_DN79992C0G1I1 | RLJ17262.1 | oadA | involved in the gluconeogenesis | C | 552.845 |
| Pec_DN7587C1G1I1 | RLJ18587.1 | BarA | phosphorelay sensor kinase activity | T | 552.228 |
| Pec_DN43291C0G1I1 | RDH88699.1 | RplY | stress response | J | 551.851 |
| Pec_DN81333C0G1I1 | RLJ20055.1 | HSP | protein folding and proteostasis | O | 542.206 |
| Pec_DN64152C0G1I1 | RDH85829.1 | AcnA | catalyses the stereo-specific isomerization of citrate to isocitrate | C | 530.666 |
| Pec_DN68028C0G1I1 | RLJ17254.1 | FabB | fatty acid biosynthesis | IQ | 523.809 |
| Pec_DN79969C0G1I1 | RLJ16287.1 | PKD | a large cell surface glycoprotein | T | 520.281 |
| Pec_DN2113C0G1I1 | RLJ15901.1 | TEF | preinitiation complex assembly | K | 516.079 |
| Pec_DN56985C0G2I1 | RDH87780.1 | DsrC | reducing sulfite to sulfide | P | 511.909 |
| Pec_DN33458C0G2I1 | AFY92545.1 | rrtT | RNA-directed DNA polymerase activity | L | 501.887 |
| Pec_DN122992C0G1I1 | RDH84341.1 | HTPA reductase | the conversion of 4-hydroxy-tetrahydrodipicolinate to tetrahydrodipicolinate | E | 463.766 |
| Pec_DN116840C0G1I1 | RDH85367.1 | GlnB | the control of glutamine synthetase | E | 462.044 |
| Pec_DN17668C0G1I1 | RLJ15729.1 | CbiA | adenosylcobalamin | H | 455.689 |
| Pec_DN34952C0G1I1 | RDH83236.1 | RISP | generates an electrochemical potential coupled to ATP synthesis | C | 454.892 |

**Table S7-3** The 70 most abundant proteins of the *Paraescarpia echinospica* as identified in the meta-proteome analysis. The protein names and their annotated accession numbers were listed on the left, these proteins were classified into different functional categories (represented by different capital letters) based on KOG database, their abundances in the trophosome of *Paraescarpia echinospica* were represented by emPAI values.

| **Sequence Name** | **Accession number** | **Abbreviations** | **Description** | **Class** | **emPAI value** |
| --- | --- | --- | --- | --- | --- |
|  |  |  |  |  | **Trophosome** |
| Pec_DN42869C0G2I1 | ALO75576.1 | HBB1 | hemoglobin subunit B1 | C | 47.59 |
| Pec_DN10730C0G1I1 | CAD29128.1 | CA | carbonic anhydrase | R | 33.86 |
| Pec_DN99862C21G1I1 | BAU46563.1 | HBV1A1 | A1 globin chain of V1 giant hemoglobin | C | 23.29 |
| Pec_DN68664C0G1I2 | XP_021326436.1 | H4L | histone H4-like | B | 14.08 |
| Pec_DN58650C10G1I1 | BAU46568.1 | HBV1L4 | L4 linker of V1 giant hemoglobin | C | 13 |
| Pec_DN12053C12G1I1 | ALO75590.1 | HBB2 | hemoglobin subunit B2 | C | 11.38 |
| Pec_DN60095C0G1I1 | BBB04239.1 | PMLI | paramyosin-like isoform X3 | Z | 7.83 |
| Pec_DN37984C0G1I1 | XP_022317112.1 | PHD | procollagen-proline dioxygenase beta subunit | O | 7.77 |
| Pec_DN51773C5G1I1 | BAU46569.1 | HBV1L1 | L1 linker of V1 giant hemoglobin | C | 7.75 |
| Pec_DN61771C0G5I1 | ELT90173.1 | Tpm | TPM_HALRU ame: Full=Tropomyosin | Z | 7.54 |
| Pec_DN67578C0G2I4 | XP_015379675.1 | CTSL1 | cathepsin L1 | O | 7.12 |
| Pec_DN35741C0G1I1 | ACL80558.1 | NCX | sodium-calcium exchanger | I | 7.04 |
| Pec_DN67186C0G1I3 | ELU08483.1 | USP | universal stress A isoform X2 | O | 7.02 |
| Pec_DN42652C0G1I1 | XP_022325998.1 | ACTP | actin 00526 | Z | 6.12 |
| Pec_DN71339C4G4I1 | NP_988915.1 | HIF1AN | hypoxia-inducible factor 1-alpha inhibitor | O | 5.79 |
| Pec_DN54947C0G1I1 | ELU13701.1 | ALDO | Fructose-biphosphate aldolase | G | 5.76 |
| Pec_DN71418C1G2I1 | XP_022339521.1 | HPDL | 4-hydroxyphenylpyruvate dioxygenase-like | E | 5.58 |
| Pec_DN42994C0G1I1 | XP_022313309.1 | TpxA | thioredoxin peroxidase | O | 5.51 |
| Pec_DN35288C0G1I1 | ALO75579.1 | HBA1 | hemoglobin subunit A1 | C | 5.46 |
| Pec_DN71465C0G1I5 | ELT94404.1 | GBL | 29-kDa galactose-binding lectin | O | 4.99 |
| Pec_DN99854C0G1I1 | ABW23229.1 | RPL30 | 60S ribosomal L30 | J | 4.87 |
| Pec_DN35305C0G1I1 | BAN58232.1 | HBV2B1 | B1 globin chain of giant V2 hemoglobin | C | 4.77 |
| Pec_DN71026C0G1I1 | XP_009025403.1 | FLNA | filamin-A-like isoform X8 | Z | 4.58 |
| Pec_DN60640C0G2I1 | AHX26698.1 | MhmA | myosin heavy striated muscle-like isoform X2 | Z | 4.56 |
| Pec_DN60695C0G1I1 | XP_003388555.1 | PPIL | peptidyl-prolyl cis-trans isomerase-like | O | 4.53 |
| Pec_DN43818C0G1I1 | XP_009015226.1 | ENO | Enolase | G | 4.03 |
| Pec_DN73629C0G1I2 | OPJ77131.1 | PRDX6 | peroxiredoxin 6 | O | 3.52 |
| Pec_DN40671C0G1I1 | ELU15616.1 | VDAC2 | voltage-dependent anion channel protein 2 | P | 3.42 |
| Pec_DN93776C0G1I1 | CAP08293.1 | MHr | HEMTM_RIFPA ame: Full=Myohemerythrin Short=MHr | C | 3.41 |
| Pec_DN63840C0G1I1 | ELU06754.1 | Transgelin | transgelin-3-like isoform X3 | Z | 3.25 |
| Pec_DN70920C5G1I4 | ELT97198.1 | TPI | triosephosphate isomerase B-like | G | 3.11 |
| Pec_DN67126C0G1I1 | XP_009019619.1 | CAP1 | Adenylyl cyclase-associated 1 | ZT | 2.75 |
| Pec_DN66145C1G1I3 | ELT91970.1 | PHYHD1 | phytanoyl- dioxygenase domain-containing 1-like | I | 2.7 |
| Pec_DN76351C18G1I1 | ALO75578.1 | HBA2 | hemoglobin subunit A2 | C | 2.66 |
| Pec_DN65293C0G1I1 | ELU01357.1 | LMNB1 | lamin-B1-like isoform X2 | DY | 2.62 |
| Pec_DN45687C0G1I1 | ACL13150.1 | CTSD | cathepsin D | O | 2.57 |
| Pec_DN65413C0G1I2 | ELT92677.1 | I-FABP | fatty acid-binding intestinal-like | I | 2.51 |
| Pec_DN23164C0G1I1 | KFP02525.1 | HSP10 | 10 kDa heat shock mitochondrial-like | O | 2.45 |
| Pec_DN70039C0G3I2 | ELT96313.1 | MPST | 3-mercaptopyruvate sulfurtransferase | V | 2.41 |
| Pec_DN64725C0G3I1 | XP_009011710.1 | L1CAM | Neural cell adhesion molecule L1 | T | 2.26 |
| Pec_DN19172C0G1I1 | ABY50192.1 | SOD1 | Cu2+/Zn2+ superoxide dismutase | P | 2.24 |
| Pec_DN72834C5G1I1 | XP_002601844.1 | ECHS1 | enoyl-CoA hydratase | I | 2.19 |
| Pec_DN72081C0G1I6 | XP_018592060.1 | TUBB | tubulin beta chain isoform X1 | Z | 2.14 |
| Pec_DN68000C0G1I1 | XP_780268.2 | PPIB | Peptidyl-prolyl cis-trans isomerase | O | 2.11 |
| Pec_DN5722C0G1I1 | ELU02655.1 | AdoHcyase | S-adenosylhomocysteine hydrolase | H | 2.07 |
| Pec_DN18261C0G1I1 | BAE93065.1 | GNB2L1 | guanine nucleotide-binding subunit beta-2-like 1 | T | 2.01 |
| Pec_DN44260C0G1I1 | XP_013384301.2 | HNRNPA3 | heterogeneous nuclear ribonucleo A3-like | A | 1.96 |
| Pec_DN100177C0G1I1 | XP_002610620.1 | PSMA6 | proteasome subunit alpha type-6-like | O | 1.96 |
| Pec_DN46259C0G1I1 | ELU01307.1 | PDIA3 | disulfide-isomerase A3-like | O | 1.93 |
| Pec_DN65284C1G1I1 | AOR07074.1 | ATP7 | ATP synthase subunit mitochondrial-like | C | 1.92 |
| Pec_DN72863C2G1I1 | XP_009017290.1 | MyoL | myophilin-like isoform X1 | Z | 1.82 |
| Pec_DN16155C0G2I1 | XP_019646151.1 | NIT2 | Nitrilase member 2 | E | 1.8 |
| Pec_DN73579C0G5I1 | ELU06258.1 | DDO | D-aspartate oxidase-like | E | 1.77 |
| Pec_DN36988C0G1I1 | XP_014474288.1 | HINT1 | histidine triad nucleotide-binding 1 | T | 1.73 |
| Pec_DN74240C0G1I1 | XP_014787765.1 | COL4A4 | collagen alpha-4(VI) chain-like | W | 1.73 |
| Pec_DN60822C0G1I1 | XP_013401274.1 | Melm | myosin essential light striated adductor muscle-like | Z | 1.71 |
| Pec_DN15725C0G2I1 | ALI58372.1 | COX5A | cytochrome c oxidase subunit Va | C | 1.71 |
| Pec_DN69787C1G1I2 | XP_022117294.1 | YWHAZ | 14-3-3 zeta | O | 1.69 |
| Pec_DN72631C1G1I1 | XP_015198879.1 | PSMA4 | proteasome subunit alpha type-4 | O | 1.66 |
| Pec_DN71560C4G2I6 | ELU00236.1 | Gld | glucose dehydrogenase [FAD, quinone]-like isoform X6 | R | 1.63 |
| Pec_DN65427C0G1I1 | ELU04699.1 | PdxS | phosphate synthase pdxS subunit | H | 1.58 |
| Pec_DN70089C0G4I1 | XP_021347171.1 | CTSL | cathepsin L1-like | O | 1.56 |
| Pec_DN63950C0G1I1 | ELU10444.1 | HSP60 | 60 kDa heat shock mitochondrial-like | O | 1.52 |
| Pec_DN67005C0G1I1 | ELU05291.1 | PDLIM7 | PDZ and LIM domain 7-like | R | 1.51 |
| Pec_DN57270C0G1I2 | XP_009011247.1 | TnT | troponin T | Z | 1.5 |
| Pec_DN73477C1G7I3 | ELT95435.1 | SELENBP1 | selenium-binding 1 | P | 1.49 |
| Pec_DN66395C0G1I1 | ELU06025.1 | SORD | Sorbitol dehydrogenase | Q | 1.49 |
| Pec_DN72437C4G1I1 | CBM42049.1 | HSP70 | heat shock -70kDa | O | 1.47 |
| Pec_DN70505C0G1I1 | ELT91957.1 | DDAH1 | N(G),N(G)-dimethylarginine dimethylaminohydrolase 1-like isoform X1 | E | 1.41 |

**Table S7-4** The 70 most abundant proteins of the symbiont of *Paraescarpia echinospica* as identified in the meta-proteome analysis. The protein names and their annotated accession numbers were listed on the left, these proteins were classified into different functional categories (represented by different capital letters) based on COG database, the proteins abundances were represented by emPAI values.

| **Sequence Name** | **Accession number** | **Abbreviations** | **Description** | **Class** | **emPAI value** |
| --- | --- | --- | --- | --- | --- |
|  |  |  |  |  | **Symbiont** |
| Pec_DN66397C0G1I1 | RLJ19266.1 | PRK | phosphoribulokinase | G | 28.38 |
| Pec_DN82760C0G1I2 | RDH86736.1 | TC.GBP | Outer membrane (porin) | M | 19.9 |
| Pec_DN96205C0G1I1 | RLJ19271.1 | RuBisCO | ribulose 1,5-bisphosphate carboxylase | G | 12.75 |
| Pec_DN116285C0G1I1 | RDH83646.1 | ADK | adenylate kinase | F | 11.58 |
| Pec_DN20410C0G1I1 | RLJ18872.1 | PSP | Predicted periplasmic/secreted protein | S | 10.85 |
| Pec_DN90200C0G1I2 | RLJ16596.1 | IDH | isocitrate dehydrogenase | C | 8.81 |
| Pec_DN122992C0G1I1 | RDH84341.1 | DapB | 4-hydroxy-tetrahydrodipicolinate reductase | E | 8.28 |
| Pec_DN40914C0G1I1 | RLJ17844.1 | PPIs | Parvulin-like peptidyl-prolyl isomerase | O | 7.26 |
| Pec_DN43464C0G1I1 | RDH88029.1 | RPS7 | 30S ribosomal S7 | J | 6.71 |
| Pec_DN86791C0G2I1 | RLJ20164.1 | PFK | pyrophosphate-dependent phosphofructokinase | G | 6.1 |
| Pec_DN42623C0G1I1 | RDH90364.1 | ILVC | ketol-acid reductoisomerase | EH | 5.98 |
| Pec_DN112300C0G1I1 | RLJ19273.1 | AAA | MoxR AAA+ ATPases | R | 5.84 |
| Pec_DN81085C0G1I1 | RLJ17628.1 | HSP60 | Chaperonin GroEL (HSP60 family) | O | 5.13 |
| Pec_DN34454C0G1I1 | RLJ16587.1 | MDH | malate dehydrogenase | C | 5.09 |
| Pec_DN33952C0G1I1 | RDH90312.1 | HflK | membrane protease subunit HflK | O | 4.95 |
| Pec_DN114044C0G1I1 | RLJ18810.1 | PPK | Polyphosphate kinase | C | 4.24 |
| Pec_DN52374C0G1I1 | RDH85138.1 | EmrA | RND family efflux MFP subunit | MV | 4.15 |
| Pec_DN65198C0G1I1 | RLJ19091.1 | ATPF1A | ATP synthase subunit alpha | C | 4.04 |
| Pec_DN34683C0G1I1 | RDH92333.1 | FBA | fructose-bisphosphate aldolase | G | 3.86 |
| Pec_DN108098C0G1I1 | RDH89035.1 | TRX | Thioredoxin 1 | O | 3.71 |
| Pec_DN76711C0G1I1 | RLJ17986.1 | AprA | adenylyl-sulfate reductase | C | 3.69 |
| Pec_DN87004C0G1I1 | RLJ15739.1 | Dsr | Dissimilatory sulfite reductase (desulfoviridin), alpha and beta subunits | P | 3.53 |
| Pec_DN90200C0G3I1 | RDH88904.1 | NadB | L-aspartate oxidase | C | 3.36 |
| Pec_DN2113C0G1I1 | RLJ15901.1 | TEF | transcription elongation factor | K | 3.28 |
| Pec_DN129392C0G1I1 | RDH83452.1 | PilQ | pilus assembly | U | 3.28 |
| Pec_DN30202C0G1I1 | RLJ20935.1 | Lyase_II | Polysaccharide lyase family domain II | G | 3.24 |
| Pec_DN32281C0G2I1 | RLJ15856.1 | PGK | phosphoglycerate kinase | G | 3.24 |
| Pec_DN78589C0G1I1 | PIK61579.1 | PSE | secreted esterase | C | 3.17 |
| Pec_DN72258C0G1I1 | RDH86786.1 | PurH | Phosphoribosylaminoimidazolecarboxamide formyltransferase/IMP cyclohydrolase | F | 3.09 |
| Pec_DN94981C0G1I1 | RLJ17985.1 | FeS | Fe-S-cluster oxidoreductase | C | 3.04 |
| Pec_DN75635C0G1I1 | RLJ19180.1 | HdrA | Heterodisulfide reductase, subunit A and related polyferredoxins | C | 2.79 |
| Pec_DN75635C0G1I1 | RLJ19180.1 | HdrB | Heterodisulfide reductase, subunit B | C | 2.79 |
| Pec_DN106870C0G1I1 | RLJ22366.1 | Mrp | Chromosome partitioning Mrp family | D | 2.73 |
| Pec_DN97519C0G1I1 | RDH89491.1 | DsbA | thiol:disulfide interchange protein DsbA | O | 2.72 |
| Pec_DN28039C0G1I1 | RDH92633.1 | Spase | signal peptidase I | U | 2.61 |
| Pec_DN56985C0G2I1 | RDH87780.1 | DsrC | Dissimilatory sulfite reductase (Desulfoviridin), gamma subunit | P | 2.58 |
| Pec_DN87004C0G4I1 | RDH88731.1 | NADPH | glutamate synthase | ER | 2.46 |
| Pec_DN65699C0G1I1 | RDH91293.1 | FDH | Formate dehydrogenase (cytochrome-c-553) | C | 2.38 |
| Pec_DN28422C0G1I1 | RLJ15899.1 | CPSase | carbamoyl-phosphate synthase small subunit | EF | 2.38 |
| Pec_DN26505C0G1I1 | RDH90552.1 | ANKR | Ankyrin repeat | T | 2.37 |
| Pec_DN96894C0G1I1 | RLJ17215.1 | AlgG | capsular biosynthesis | M | 2.32 |
| Pec_DN90200C0G2I1 | RLJ16597.1 | SucD | Succinyl-CoA synthetase, alpha subunit | C | 2.26 |
| Pec_DN66768C0G1I2 | RLJ21590.1 | SDHA | Succinate dehydrogenase or fumarate flavo subunit | C | 2.2 |
| Pec_DN51096C2G1I1 | RLJ20046.1 | PHGDH | D-3-phosphoglycerate dehydrogenase | HR | 2.16 |
| Pec_DN49349C0G1I1 | RDH89034.1 | Rho | transcription termination factor Rho | K | 2.15 |
| Pec_DN102403C0G1I1 | RDH90111.1 | SseA | Rhodanese-related sulfurtransferase | P | 2.14 |
| Pec_DN67579C1G1I1 | RLJ20677.1 | SucC | Succinyl-CoA synthetase, beta subunit | C | 2.12 |
| Pec_DN35045C0G2I1 | RDH86588.1 | Mla | ABC-type transporter Mla maintaining outer membrane lipid periplasmic component | I | 2.11 |
| Pec_DN70097C0G1I1 | RLJ21875.1 | TrpB | tryptophan synthase subunit beta | E | 1.98 |
| Pec_DN117820C0G1I1 | RDH89289.1 | WbpV | NAD-dependent epimerase/dehydratase | M | 1.97 |
| Pec_DN111935C0G1I1 | RDH85575.1 | PstS | ABC-type phosphate transport system, periplasmic component | P | 1.93 |
| Pec_DN73810C0G1I1 | RLJ15929.1 | PNP | polyribonucleotide nucleotidyltransferase | J | 1.88 |
| Pec_DN90865C0G1I1 | RLJ19646.1 | TC.OOP | OmpA-OmpF porin, OOP family | M | 1.87 |
| Pec_DN39694C1G1I1 | RDH92037.1 | DsbC | thiol:disulfide interchange protein DsbC | O | 1.86 |
| Pec_DN68595C0G1I1 | RDH80980.1 | GLUL | glutamine synthetase | E | 1.85 |
| Pec_DN58694C0G1I1 | RLJ19053.1 | KorB | 2-oxoglutarate oxidoreductase | C | 1.69 |
| Pec_DN104157C0G1I1 | RLJ19348.1 | BDS1 | Alkyl sulfatase BDS1 and related hydrolases, metallo-beta-lactamase superfamily | Q | 1.68 |
| Pec_DN824C0G1I1 | RDH82198.1 | GGT | gamma-glutamyltranspeptidase | E | 1.65 |
| Pec_DN101502C0G1I1 | RLJ22311.1 | MRI1 | methylthioribose-1-phosphate isomerase | E | 1.6 |
| Pec_DN71222C0G1I1 | RLJ17351.1 | PFOR | pyruvate ferredoxin oxidoreductase | C | 1.48 |
| Pec_DN622C0G1I1 | RDH87115.1 | MDR | Multidrug resistance efflux pump | V | 1.43 |
| Pec_DN34952C0G1I1 | RDH83236.1 | UQCRFS1 | ubiquinol-cytochrome c reductase iron-sulfur subunit | C | 1.39 |
| Pec_DN116840C0G1I1 | RDH85367.1 | GlnB | nitrogen regulatory P-II | TE | 1.34 |
| Pec_DN83696C0G1I2 | RDH89092.1 | N2OR | Nitrous-oxide reductase | P | 1.23 |
| Pec_DN73529C0G1I1 | RLJ15858.1 | TKT | transketolase | G | 1.21 |
| Pec_DN81681C2G1I1 | RLJ16667.1 | NuoG | NADH-quinone oxidoreductase subunit G | C | 1.16 |
| Pec_DN8044C0G1I1 | RDH90913.1 | TrxB | thioredoxin reductase | O | 1.12 |
| Pec_DN25009C0G1I1 | RDH82204.1 | PMPCA | mitochondrial-processing peptidase subunit alpha | R | 1.11 |
| Pec_DN62148C1G1I1 | RDH88871.1 | AS | Anthranilate/para-aminobenzoate synthases component I | EH | 1.09 |

Functional categories description

[CELLULAR PROCESSES AND SIGNALING] D - Cell cycle control, cell division, chromosome partitioning; M - Cell wall/membrane/envelope biogenesis; N - Cell motility; O - Post-translational modification, protein turnover, and chaperones; T - Signal transduction mechanisms; U - Intracellular trafficking, secretion, and vesicular transport; V - Defense mechanisms; W - Extracellular structures; Y - Nuclear structure; Z - Cytoskeleton; [INFORMATION STORAGE AND PROCESSING] A - RNA processing and modification; B - Chromatin structure and dynamics; J - Translation, ribosomal structure and biogenesis; K - Transcription; L - Replication, recombination and repair; [METABOLISM] C - Energy production and conversion; E - Amino acid transport and metabolism; F - Nucleotide transport and metabolism; G - Carbohydrate transport and metabolism; H - Coenzyme transport and metabolism; I - Lipid transport and metabolism; P - Inorganic ion transport and metabolism; Q - Secondary metabolites biosynthesis, transport, and catabolism; [POORLY CHARACTERIZED] R - General function prediction only; S - Function unknown

*Note*: Functionally redundant gene annotations and most genes of unknown function are excluded in this table. The annotation of complete transcriptomic and proteomic data set is shown in Supplementary Excel Table S7.

**Table S9** Genes involved in energy production, transportation and conversion in the *P. echinospica* symbiont genome analysis

| **KEGG pathways** | | **Genes names** | | **Accession number** | **Description (Proteins)** |
| --- | --- | --- | --- | --- | --- |
| **ABC transporters** | | | | | |
| **Mineral and organic ion transporters** | Molybdate | *modABC* | | RLJ18164.1, RLJ22334.1, RDH92271.1 |  |
|  | Iron (III) | *afuABC* | | RLJ19920.1, RLJ19587.1, RDH88954.1 | Iron(III) transport system related proteins |
|  | Phospholipid | *mlaBCDEF* | | RLJ18783.1, RDH86852.1, RDH88882.1, RLJ18784.1, RDH84628.1 | Phospholipid transport system transporter/ substrate -binding proteins |
| **Phosphate transporters** | Phosphate | *pstABCS* | | RDH87243.1, RDH88276.1, RDH88279.1, RDH88281.1 | Phosphate transport system related proteins |
| **Metallic cation, iron-siderophore transporters** | Iron complex | *afuABC* | | RLJ19920.1, RLJ19587.1, RDH88954.1 | Iron complex transport system related proteins |
|  | Zinc | *znuABC* | | RDH81805.1, RLJ17988.1, RDH81804.1 | Zinc transport system related proteins |
| **ABC-2 and other transporters** | Heme | *ccmABCD* | | RDH90898.1, RDH90899.1, RDH90900.1, RDH86318.1 | Heme exporter proteins |
|  | Capsular polysaccharide | *kpsEMT* | | RLJ20337.1, RLJ20336.1, RLJ20555.1 | Capsular polysaccharide transport system proteins |
|  | Lipoprotein | *lolC_ED* | | RDH89221.1, RDH86303.1 | Lipoprotein-releasing system proteins |
|  | Lipopolysaccharide | *lptBFG* | | RDH86729.1, RDH80836.1, RLJ16493.1 | Lipopolysaccharide transport system proteins |
| **Two-component system** | |  | |  |  |
| **Phosphate assimilation** | | *phoABB1DPR* | | RDH90586.1, RLJ21157.1, RDH84219.1, RLJ18322.1, RDH90511.1, RDH88274.1 | Phosphate assimilation related proteins under phosphate limitation condition |
| **Nitrogen metabolism** | Nitrogen assimilation | *glnABDGL, nifA*, *ntrXY* | | RLJ21576.1, PCI83366.1, RDH87754.1, RLJ20304.1, RLJ18601.1; RLJ18689.1; RLJ19000.1, RDH89408.1 | Nitrogen assimilation related proteins under nitrogen availability low condition |
|  | Denitrification | *narI, napAB, nirK, norBC, nosZ* | | RLJ15733.1; RLJ16182.1, RLJ17711.1; RLJ20391.1; RLJ18034.1, RLJ20932.1; RDH89092.1 | Nitrate respiration |
|  | Ammonia assimilation | *glnA, gltBD, GLU, GLUD1_2* | | RLJ21576.1; RLJ21183.1, RDH88305.1; RDH84200.1; RLJ15825.1 | Ammonia was incorporated into L-glutamate to form L-glutamine |
|  | Dissimilatory nitrate reduction | *narI, napAB, nirBD* | | RLJ15733.1; RLJ16182.1, RLJ17711.1; RLJ16182.1 | From nitrate to nitrite and then to ammonia |
| **Porphyrin and chlorophyll metabolism** | | |  | | |
| **Protoheme (Heme) generation** | | *hemABCDEHLNQ* | | RDH86713.1, RDH87161.1, RDH89403.1, RLJ20124.1, RLJ19128.1, RLJ15692.1, RLJ18043.1, RLJ16417.1, RDH90957.1 | Proteins involved in heme generated from glutamate |
| **Ferritin-Fe synthesis** | | *hemABCDELN*, *CP* | | RDH86713.1, RDH87161.1, RDH89403.1, RLJ20124.1, RLJ19128.1, RLJ18043.1  RLJ16417.1; RLJ22247.1 | Participation in Ferritin-Fe synthesis from glutamate |
| **Carbon fixation** | |  | |  |  |
| **Reverse Krebs cycle (rTCA cycle)** | | *ACLY*, *mdh*, *IDH1*, *porA*, *por*, *korAB*, *sdhAC*, *frdB*, *ACO*, *sucCD*, *pps, fumAB* | | RDH88117.1; RLJ16587.1; RLJ18863.1; RDH89285.1; RLJ16606.1, RLJ21060.1; RLJ19530.1, RLJ16787.1; RDH89618.1; RDH85829.1; RLJ20677.1, RLJ19059.1; RLJ17344.1; RLJ21936.1, RLJ22524.1 | Producing carbon compounds from CO_2_ and water |
| **CBB cycle** | | *PGK*, *GAPDH*, *TPI*, *FBA*, *rpiA*, *rpe*, *PRK*, *rbcL* | | RLJ15856.1; RLJ20190.1; RLJ15907.1; RDH92333.1; RDH86737.1; RLJ18799.1; RLJ19271.1; RLJ19266.1 | Converting CO_2_ and other compounds into glucose |
| **Formate generation** | | *fdhAB* | | RDH87223.1, RDH88912.1 | Formate dehydrogenase, forming formate from CO_2_ |
| **Carbon metabolism** | |  | |  |  |
| **Citrate cycle (TCA cycle)** | | *mdh*, *IDH1*, *aceE*, *OGDH*, *porA*, *korAB*, *sdhAC*, *frdB*, *DLD*, *DLAT*, *DLST*, *CS*, *ACLY*, *fumAB*, *ACO*, *acnB*, *sucCD*, *aclB* | | RLJ16587.1; RLJ18863.1; RDH90426.1; RLJ16777.1; RDH89285.1; RLJ16606.1, RLJ21060.1; RLJ19530.1, RLJ16787.1; RDH89618.1; RLJ16231.1; RDH87757.1; RDH84227.1; RLJ20698.1; RDH88117.1; RLJ21936.1; RDH85829.1; WP_005964385.1; RLJ19059.1, RLJ20677.1; RLJ22524.1; RDH88117.1 | Released stored energy and producing carbon dioxide |
| **Glycolysis** | | *pgm*, *pmm-pgm*, *GPI*, *pfkA*, *ALDO*, *FBA*, *TPI*, *GADPH*, *gapN*, *PGK*, *gpmIB*, *apgM*, *PGAM*, *ENO*, *PK* | | RDH83644.1; RDH82074.1; RLJ16147.1; RLJ19068.1; RLJ18468.1; RDH92333.1; RLJ15907.1; RLJ20190.1; RLJ15982.1; RLJ15856.1; RLJ22657.1, RLJ16867.1; RLJ21295.1; RLJ16867.1; RDH90594.1; RLJ19627.1 | Converting glucose into pyruvate and generating small amounts of ATP (energy) and NADH |
| **Formate oxidation** | | *FDH*, *fdoGHI*, *fdhAB* | | RDH87223.1, RDH88912.1; PLY12719.1, RDH91810.1; | Formate oxidation |
| **Sulfur metabolism** | |  | |  |  |
| **Dissimilatory sulfate reduction and oxidation** | | *sat*, *aprAB*, *dsrAB* | | RDH86236.1; RLJ17986.1, RLJ19170.1; RDH87776.1, RLJ19443.1 | Dissimilatory sulfur reduction enzymes forming a sulfur oxidation pathway from sulfite to APS and then to sulfate |
| **Sulfide and thiosulfate oxidation** | | *sox*, *sat*, *ttrB*, *dsrAB*, *aprAB*, *phsC*, *glpE*, *TST* | | RDH86236.1; RLJ19704.1; RDH87776.1, RLJ19443.1; RLJ17986.1, RLJ19170.1; RDH88148.1; RDH87792.1; | Enzymes forming sulfide and thiosulfate oxidation pathways |
| **SOX system** | | *soxABYZ* | | RLJ19577.1, RLJ21246.1, RLJ15866.1, RLJ18322.1 | A sulfur oxidation pathway from thiosulfate to sulfate |

**Table S10** Significantly enriched GO terms of highly expressed transcripts (TPM ratio > 0.75) in the trophosome of *Paraescarpia echinospica*

| GOID | Ontology | Term | Level | Number in your list (q) | Number on your customized array (m) | [Log odds-ratio](http://omicslab.genetics.ac.cn/GOEAST/faq.php#results-3_anchor) | [p-value](http://omicslab.genetics.ac.cn/GOEAST/faq.php#results-4_anchor) |
| --- | --- | --- | --- | --- | --- | --- | --- |
| [GO:0003674](http://amigo.geneontology.org/cgi-bin/amigo/go.cgi?query=GO%3A0003674&search_constraint=terms&action=query&view=query) | molecular_function | molecular_function | 0 | 544 | 8990 | 0.187 | 1.5e-3 |
| [GO:0019825](http://amigo.geneontology.org/cgi-bin/amigo/go.cgi?query=GO%3A0019825&search_constraint=terms&action=query&view=query) | molecular_function | oxygen binding | 1 | 19 | 39 | 3.196 | 1.5e-11 |
| [GO:0005833](http://amigo.geneontology.org/cgi-bin/amigo/go.cgi?query=GO%3A0005833&search_constraint=terms&action=query&view=query) | cellular_component | hemoglobin complex | 5 | 18 | 26 | 3.703 | 3.8e-14 |
| [GO:0044445](http://amigo.geneontology.org/cgi-bin/amigo/go.cgi?query=GO%3A0044445&search_constraint=terms&action=query&view=query) | cellular_component | cytosolic part | 5 | 19 | 90 | 1.990 | 6.9e-5 |
| [GO:0005506](http://amigo.geneontology.org/cgi-bin/amigo/go.cgi?query=GO%3A0005506&search_constraint=terms&action=query&view=query) | molecular_function | iron ion binding | 1 | 37 | 119 | 2.548 | 2.8e-15 |
| [GO:0006810](http://amigo.geneontology.org/cgi-bin/amigo/go.cgi?query=GO%3A0006810&search_constraint=terms&action=query&view=query) | biological_process | transport | 1 | 148 | 1460 | 0.931 | 9.4e-12 |
| [GO:0008150](http://amigo.geneontology.org/cgi-bin/amigo/go.cgi?query=GO%3A0008150&search_constraint=terms&action=query&view=query) | biological_process | biological_process | 0 | 523 | 8077 | 0.285 | 1.3e-7 |
| [GO:0015669](http://amigo.geneontology.org/cgi-bin/amigo/go.cgi?query=GO%3A0015669&search_constraint=terms&action=query&view=query) | biological_process | gas transport | 3 | 22 | 43 | 3.267 | 6.0e-14 |
| [GO:0015671](http://amigo.geneontology.org/cgi-bin/amigo/go.cgi?query=GO%3A0015671&search_constraint=terms&action=query&view=query) | biological_process | oxygen transport | 3 | 22 | 43 | 3.267 | 6.0e-14 |
| [GO:0044699](http://amigo.geneontology.org/cgi-bin/amigo/go.cgi?query=GO%3A0044699&search_constraint=terms&action=query&view=query) | biological_process | single-organism process | 1 | 329 | 4184 | 0.565 | 1.1e-12 |
| [GO:0044765](http://amigo.geneontology.org/cgi-bin/amigo/go.cgi?query=GO%3A0044765&search_constraint=terms&action=query&view=query) | biological_process | single-organism transport | 3 | 130 | 1066 | 1.198 | 2. 7e-15 |
| [GO:0051179](http://amigo.geneontology.org/cgi-bin/amigo/go.cgi?query=GO%3A0051179&search_constraint=terms&action=query&view=query) | biological_process | localization | 1 | 160 | 1577 | 0.933 | 7.2e-13 |
| [GO:0051234](http://amigo.geneontology.org/cgi-bin/amigo/go.cgi?query=GO%3A0051234&search_constraint=terms&action=query&view=query) | biological_process | establishment of localization | 1 | 148 | 1485 | 0.907 | 2.7e-11 |
| [GO:1902578](http://amigo.geneontology.org/cgi-bin/amigo/go.cgi?query=GO%3A1902578&search_constraint=terms&action=query&view=query) | biological_process | single-organism localization | 2 | 130 | 1093 | 1.162 | 5.7e-15 |
| [GO:0020037](http://amigo.geneontology.org/cgi-bin/amigo/go.cgi?query=GO%3A0020037&search_constraint=terms&action=query&view=query) | molecular_function | heme binding | 2 | 29 | 104 | 2.391 | 6.1e-11 |
| [GO:0046906](http://amigo.geneontology.org/cgi-bin/amigo/go.cgi?query=GO%3A0046906&search_constraint=terms&action=query&view=query) | molecular_function | tetrapyrrole binding | 2 | 31 | 112 | 2.381 | 1.5e-11 |
| [GO:0005215](http://amigo.geneontology.org/cgi-bin/amigo/go.cgi?query=GO%3A0005215&search_constraint=terms&action=query&view=query) | molecular_function | transporter activity | 1 | 112 | 855 | 1.301 | 2.7e-15 |
| [GO:0005344](http://amigo.geneontology.org/cgi-bin/amigo/go.cgi?query=GO%3A0005344&search_constraint=terms&action=query&view=query) | molecular_function | oxygen transporter activity | 1 | 22 | 43 | 3.267 | 6.0e-14 |
| [GO:0022892](http://amigo.geneontology.org/cgi-bin/amigo/go.cgi?query=GO%3A0022892&search_constraint=terms&action=query&view=query) | molecular_function | substrate-specific transporter activity | 1 | 68 | 673 | 0.927 | 8.2e-5 |
| [GO:0005576](http://amigo.geneontology.org/cgi-bin/amigo/go.cgi?query=GO%3A0005576&search_constraint=terms&action=query&view=query) | cellular_component | extracellular region | 1 | 84 | 629 | 1.329 | 6.8e-12 |
| [GO:0003824](http://amigo.geneontology.org/cgi-bin/amigo/go.cgi?query=GO%3A0003824&search_constraint=terms&action=query&view=query) | molecular_function | catalytic activity | 1 | 281 | 4312 | 0.294 | 9.5e-3 |
| [GO:0016020](http://amigo.geneontology.org/cgi-bin/amigo/go.cgi?query=GO%3A0016020&search_constraint=terms&action=query&view=query) | cellular_component | membrane | 1 | 313 | 4586 | 0.361 | 5.9e-5 |
| [GO:0016021](http://amigo.geneontology.org/cgi-bin/amigo/go.cgi?query=GO%3A0016021&search_constraint=terms&action=query&view=query) | cellular_component | integral component of membrane | 2 | 294 | 4084 | 0.438 | 1.0e-6 |
| [GO:0031224](http://amigo.geneontology.org/cgi-bin/amigo/go.cgi?query=GO%3A0031224&search_constraint=terms&action=query&view=query) | cellular_component | intrinsic component of membrane | 2 | 294 | 4092 | 0.435 | 1.2e-6 |
| [GO:0044425](http://amigo.geneontology.org/cgi-bin/amigo/go.cgi?query=GO%3A0044425&search_constraint=terms&action=query&view=query) | cellular_component | membrane part | 2 | 295 | 4233 | 0.391 | 2.3e-5 |
| [GO:0022804](http://amigo.geneontology.org/cgi-bin/amigo/go.cgi?query=GO%3A0022804&search_constraint=terms&action=query&view=query) | molecular_function | active transmembrane transporter activity | 1 | 29 | 172 | 1.665 | 1.3e-5 |
| [GO:0022857](http://amigo.geneontology.org/cgi-bin/amigo/go.cgi?query=GO%3A0022857&search_constraint=terms&action=query&view=query) | molecular_function | transmembrane transporter activity | 1 | 58 | 628 | 0.797 | 6.3e-3 |
| [GO:0044763](http://amigo.geneontology.org/cgi-bin/amigo/go.cgi?query=GO%3A0044763&search_constraint=terms&action=query&view=query) | biological_process | single-organism cellular process | 2 | 232 | 3470 | 0.331 | 1.2e-2 |
| [GO:0055085](http://amigo.geneontology.org/cgi-bin/amigo/go.cgi?query=GO%3A0055085&search_constraint=terms&action=query&view=query) | biological_process | transmembrane transport | 4 | 88 | 771 | 1.103 | 4.6e-9 |
| [GO:0015297](http://amigo.geneontology.org/cgi-bin/amigo/go.cgi?query=GO%3A0015297&search_constraint=terms&action=query&view=query) | molecular_function | antiporter activity | 1 | 7 | 25 | 2.397 | 3.8e-2 |
| [GO:0006855](http://amigo.geneontology.org/cgi-bin/amigo/go.cgi?query=GO%3A0006855&search_constraint=terms&action=query&view=query) | biological_process | drug transmembrane transport | 5 | 6 | 13 | 3.118 | 6.0e-3 |
| [GO:0015893](http://amigo.geneontology.org/cgi-bin/amigo/go.cgi?query=GO%3A0015893&search_constraint=terms&action=query&view=query) | biological_process | drug transport | 4 | 6 | 13 | 3.118 | 6.0e-3 |
| [GO:0042493](http://amigo.geneontology.org/cgi-bin/amigo/go.cgi?query=GO%3A0042493&search_constraint=terms&action=query&view=query) | biological_process | response to drug | 1 | 7 | 26 | 2.341 | 4.7e-2 |
| [GO:0015238](http://amigo.geneontology.org/cgi-bin/amigo/go.cgi?query=GO%3A0015238&search_constraint=terms&action=query&view=query) | molecular_function | drug transmembrane transporter activity | 2 | 6 | 13 | 3.118 | 6.0e-3 |
| [GO:0090484](http://amigo.geneontology.org/cgi-bin/amigo/go.cgi?query=GO%3A0090484&search_constraint=terms&action=query&view=query) | molecular_function | drug transporter activity | 1 | 6 | 13 | 3.118 | 6.0e-3 |
| [GO:0005615](http://amigo.geneontology.org/cgi-bin/amigo/go.cgi?query=GO%3A0005615&search_constraint=terms&action=query&view=query) | cellular_component | extracellular space | 2 | 36 | 165 | 2.037 | 1.9e-10 |
| [GO:0044421](http://amigo.geneontology.org/cgi-bin/amigo/go.cgi?query=GO%3A0044421&search_constraint=terms&action=query&view=query) | cellular_component | extracellular region part | 2 | 50 | 399 | 1.237 | 6.5e-6 |
| [GO:0006033](http://amigo.geneontology.org/cgi-bin/amigo/go.cgi?query=GO%3A0006033&search_constraint=terms&action=query&view=query) | biological_process | chitin localization | 1 | 14 | 29 | 3.183 | 2.7e-8 |
| [GO:0033037](http://amigo.geneontology.org/cgi-bin/amigo/go.cgi?query=GO%3A0033037&search_constraint=terms&action=query&view=query) | biological_process | polysaccharide localization | 1 | 14 | 31 | 3.087 | 7.8e-8 |
| [GO:0030198](http://amigo.geneontology.org/cgi-bin/amigo/go.cgi?query=GO%3A0030198&search_constraint=terms&action=query&view=query) | biological_process | extracellular matrix organization | 3 | 14 | 44 | 2.582 | 1.3e-5 |
| [GO:0043062](http://amigo.geneontology.org/cgi-bin/amigo/go.cgi?query=GO%3A0043062&search_constraint=terms&action=query&view=query) | biological_process | extracellular structure organization | 3 | 14 | 44 | 2.582 | 1.3e-5 |
| [GO:0015399](http://amigo.geneontology.org/cgi-bin/amigo/go.cgi?query=GO%3A0015399&search_constraint=terms&action=query&view=query) | molecular_function | primary active transmembrane transporter activity | 1 | 17 | 75 | 2.092 | 9.5e-5 |
| [GO:0015405](http://amigo.geneontology.org/cgi-bin/amigo/go.cgi?query=GO%3A0015405&search_constraint=terms&action=query&view=query) | molecular_function | P-P-bond-hydrolysis-driven transmembrane transporter activity | 1 | 17 | 75 | 2.092 | 9.5e-5 |
| [GO:0016820](http://amigo.geneontology.org/cgi-bin/amigo/go.cgi?query=GO%3A0016820&search_constraint=terms&action=query&view=query) | molecular_function | hydrolase activity, acting on acid anhydrides, catalyzing transmembrane movement of substances | 2 | 17 | 76 | 2.073 | 1.1e-4 |
| [GO:0042626](http://amigo.geneontology.org/cgi-bin/amigo/go.cgi?query=GO%3A0042626&search_constraint=terms&action=query&view=query) | molecular_function | ATPase activity, coupled to transmembrane movement of substances | 3 | 17 | 73 | 2.131 | 6.9e-5 |
| [GO:0043492](http://amigo.geneontology.org/cgi-bin/amigo/go.cgi?query=GO%3A0043492&search_constraint=terms&action=query&view=query) | molecular_function | ATPase activity, coupled to movement of substances | 1 | 17 | 78 | 2.036 | 1.7e-4 |
| [GO:0016491](http://amigo.geneontology.org/cgi-bin/amigo/go.cgi?query=GO%3A0016491&search_constraint=terms&action=query&view=query) | molecular_function | oxidoreductase activity | 1 | 71 | 504 | 1.406 | 3.9e-11 |
| [GO:0044710](http://amigo.geneontology.org/cgi-bin/amigo/go.cgi?query=GO%3A0044710&search_constraint=terms&action=query&view=query) | biological_process | single-organism metabolic process | 2 | 122 | 1255 | 0.871 | 2.0e-8 |
| [GO:0055114](http://amigo.geneontology.org/cgi-bin/amigo/go.cgi?query=GO%3A0055114&search_constraint=terms&action=query&view=query) | biological_process | oxidation-reduction process | 2 | 74 | 554 | 1.329 | 1.5e-10 |
| [GO:0016705](http://amigo.geneontology.org/cgi-bin/amigo/go.cgi?query=GO%3A0016705&search_constraint=terms&action=query&view=query) | molecular_function | oxidoreductase activity, acting on paired donors, with incorporation or reduction of molecular oxygen | 1 | 14 | 89 | 1.565 | 3.8e-2 |
| [GO:0004857](http://amigo.geneontology.org/cgi-bin/amigo/go.cgi?query=GO%3A0004857&search_constraint=terms&action=query&view=query) | molecular_function | enzyme inhibitor activity | 1 | 26 | 106 | 2.206 | 2.0e-8 |
| [GO:0004866](http://amigo.geneontology.org/cgi-bin/amigo/go.cgi?query=GO%3A0004866&search_constraint=terms&action=query&view=query) | molecular_function | endopeptidase inhibitor activity | 3 | 26 | 78 | 2.649 | 1.5e-11 |
| [GO:0030234](http://amigo.geneontology.org/cgi-bin/amigo/go.cgi?query=GO%3A0030234&search_constraint=terms&action=query&view=query) | molecular_function | enzyme regulator activity | 1 | 26 | 184 | 1.411 | 1.2e-3 |
| [GO:0030414](http://amigo.geneontology.org/cgi-bin/amigo/go.cgi?query=GO%3A0030414&search_constraint=terms&action=query&view=query) | molecular_function | peptidase inhibitor activity | 2 | 26 | 81 | 2.594 | 3.5e-11 |
| [GO:0061134](http://amigo.geneontology.org/cgi-bin/amigo/go.cgi?query=GO%3A0061134&search_constraint=terms&action=query&view=query) | molecular_function | peptidase regulator activity | 1 | 26 | 83 | 2.559 | 6.0e-11 |
| [GO:0061135](http://amigo.geneontology.org/cgi-bin/amigo/go.cgi?query=GO%3A0061135&search_constraint=terms&action=query&view=query) | molecular_function | endopeptidase regulator activity | 1 | 26 | 78 | 2.649 | 1.5e-11 |
| [GO:0009892](http://amigo.geneontology.org/cgi-bin/amigo/go.cgi?query=GO%3A0009892&search_constraint=terms&action=query&view=query) | biological_process | negative regulation of metabolic process | 3 | 28 | 238 | 1.146 | 1.4e-2 |
| [GO:0010466](http://amigo.geneontology.org/cgi-bin/amigo/go.cgi?query=GO%3A0010466&search_constraint=terms&action=query&view=query) | biological_process | negative regulation of peptidase activity | 8 | 26 | 84 | 2.542 | 7.6e-11 |
| [GO:0010605](http://amigo.geneontology.org/cgi-bin/amigo/go.cgi?query=GO%3A0010605&search_constraint=terms&action=query&view=query) | biological_process | negative regulation of macromolecule metabolic process | 3 | 28 | 222 | 1.247 | 4.6e-3 |
| [GO:0010951](http://amigo.geneontology.org/cgi-bin/amigo/go.cgi?query=GO%3A0010951&search_constraint=terms&action=query&view=query) | biological_process | negative regulation of endopeptidase activity | 9 | 26 | 81 | 2.594 | 3.5e-11 |
| [GO:0030162](http://amigo.geneontology.org/cgi-bin/amigo/go.cgi?query=GO%3A0030162&search_constraint=terms&action=query&view=query) | biological_process | regulation of proteolysis | 3 | 27 | 118 | 2.106 | 4.5e-8 |
| [GO:0031324](http://amigo.geneontology.org/cgi-bin/amigo/go.cgi?query=GO%3A0031324&search_constraint=terms&action=query&view=query) | biological_process | negative regulation of cellular metabolic process | 5 | 28 | 230 | 1.196 | 8.0e-3 |
| [GO:0032268](http://amigo.geneontology.org/cgi-bin/amigo/go.cgi?query=GO%3A0032268&search_constraint=terms&action=query&view=query) | biological_process | regulation of cellular protein metabolic process | 4 | 28 | 263 | 1.002 | 5.3e-2 |
| [GO:0032269](http://amigo.geneontology.org/cgi-bin/amigo/go.cgi?query=GO%3A0032269&search_constraint=terms&action=query&view=query) | biological_process | negative regulation of cellular protein metabolic process | 7 | 28 | 130 | 2.019 | 8.6e-8 |
| [GO:0043086](http://amigo.geneontology.org/cgi-bin/amigo/go.cgi?query=GO%3A0043086&search_constraint=terms&action=query&view=query) | biological_process | negative regulation of catalytic activity | 2 | 26 | 133 | 1.879 | 3.0e-6 |
| [GO:0044092](http://amigo.geneontology.org/cgi-bin/amigo/go.cgi?query=GO%3A0044092&search_constraint=terms&action=query&view=query) | biological_process | negative regulation of molecular function | 1 | 26 | 156 | 1.649 | 6.9e-5 |
| [GO:0045861](http://amigo.geneontology.org/cgi-bin/amigo/go.cgi?query=GO%3A0045861&search_constraint=terms&action=query&view=query) | biological_process | negative regulation of proteolysis | 8 | 26 | 87 | 2.491 | 1.7e-10 |
| [GO:0051246](http://amigo.geneontology.org/cgi-bin/amigo/go.cgi?query=GO%3A0051246&search_constraint=terms&action=query&view=query) | biological_process | regulation of protein metabolic process | 3 | 29 | 275 | 0.988 | 5.0e-2 |
| [GO:0051248](http://amigo.geneontology.org/cgi-bin/amigo/go.cgi?query=GO%3A0051248&search_constraint=terms&action=query&view=query) | biological_process | negative regulation of protein metabolic process | 4 | 28 | 131 | 2.008 | 1.0e-7 |
| [GO:0051336](http://amigo.geneontology.org/cgi-bin/amigo/go.cgi?query=GO%3A0051336&search_constraint=terms&action=query&view=query) | biological_process | regulation of hydrolase activity | 1 | 27 | 248 | 1.034 | 4.8e-2 |
| [GO:0051346](http://amigo.geneontology.org/cgi-bin/amigo/go.cgi?query=GO%3A0051346&search_constraint=terms&action=query&view=query) | biological_process | negative regulation of hydrolase activity | 3 | 26 | 95 | 2.364 | 1.5e-9 |
| [GO:0052547](http://amigo.geneontology.org/cgi-bin/amigo/go.cgi?query=GO%3A0052547&search_constraint=terms&action=query&view=query) | biological_process | regulation of peptidase activity | 3 | 27 | 97 | 2.389 | 3.7e-10 |
| [GO:0052548](http://amigo.geneontology.org/cgi-bin/amigo/go.cgi?query=GO%3A0052548&search_constraint=terms&action=query&view=query) | biological_process | regulation of endopeptidase activity | 3 | 27 | 94 | 2.434 | 1.7e-10 |
| [GO:0006082](http://amigo.geneontology.org/cgi-bin/amigo/go.cgi?query=GO%3A0006082&search_constraint=terms&action=query&view=query) | biological_process | organic acid metabolic process | 5 | 35 | 252 | 1.386 | 6.8e-5 |
| [GO:0019752](http://amigo.geneontology.org/cgi-bin/amigo/go.cgi?query=GO%3A0019752&search_constraint=terms&action=query&view=query) | biological_process | carboxylic acid metabolic process | 5 | 30 | 224 | 1.333 | 8.0e-4 |
| [GO:0043436](http://amigo.geneontology.org/cgi-bin/amigo/go.cgi?query=GO%3A0043436&search_constraint=terms&action=query&view=query) | biological_process | oxoacid metabolic process | 5 | 31 | 228 | 1.355 | 4.30e-4 |
| [GO:0044281](http://amigo.geneontology.org/cgi-bin/amigo/go.cgi?query=GO%3A0044281&search_constraint=terms&action=query&view=query) | biological_process | small molecule metabolic process | 2 | 59 | 536 | 1.050 | 3.4e-5 |
| [GO:0006520](http://amigo.geneontology.org/cgi-bin/amigo/go.cgi?query=GO%3A0006520&search_constraint=terms&action=query&view=query) | biological_process | cellular amino acid metabolic process | 5 | 23 | 130 | 1.735 | 1.0e-4 |
| [GO:0009063](http://amigo.geneontology.org/cgi-bin/amigo/go.cgi?query=GO%3A0009063&search_constraint=terms&action=query&view=query) | biological_process | cellular amino acid catabolic process | 9 | 8 | 22 | 2.774 | 2.4e-3 |
| [GO:0009064](http://amigo.geneontology.org/cgi-bin/amigo/go.cgi?query=GO%3A0009064&search_constraint=terms&action=query&view=query) | biological_process | glutamine family amino acid metabolic process | 5 | 11 | 36 | 2.523 | 4.5e-4 |
| [GO:0016054](http://amigo.geneontology.org/cgi-bin/amigo/go.cgi?query=GO%3A0016054&search_constraint=terms&action=query&view=query) | biological_process | organic acid catabolic process | 8 | 9 | 42 | 2.011 | 4.5e-2 |
| [GO:0044282](http://amigo.geneontology.org/cgi-bin/amigo/go.cgi?query=GO%3A0044282&search_constraint=terms&action=query&view=query) | biological_process | small molecule catabolic process | 3 | 10 | 54 | 1.801 | 6.6e-2 |
| [GO:0044712](http://amigo.geneontology.org/cgi-bin/amigo/go.cgi?query=GO%3A0044712&search_constraint=terms&action=query&view=query) | biological_process | single-organism catabolic process | 3 | 17 | 131 | 1.288 | 7.6e-2 |
| [GO:0046395](http://amigo.geneontology.org/cgi-bin/amigo/go.cgi?query=GO%3A0046395&search_constraint=terms&action=query&view=query) | biological_process | carboxylic acid catabolic process | 9 | 9 | 38 | 2.156 | 2.4e-2 |
| [GO:1901565](http://amigo.geneontology.org/cgi-bin/amigo/go.cgi?query=GO%3A1901565&search_constraint=terms&action=query&view=query) | biological_process | organonitrogen compound catabolic process | 3 | 15 | 99 | 1.511 | 3.6e-2 |
| [GO:1901605](http://amigo.geneontology.org/cgi-bin/amigo/go.cgi?query=GO%3A1901605&search_constraint=terms&action=query&view=query) | biological_process | alpha-amino acid metabolic process | 5 | 18 | 73 | 2.214 | 1.3e-5 |
| [GO:0008168](http://amigo.geneontology.org/cgi-bin/amigo/go.cgi?query=GO%3A0008168&search_constraint=terms&action=query&view=query) | molecular_function | methyltransferase activity | 1 | 27 | 259 | 0.972 | 8.5e-2 |
| [GO:0048037](http://amigo.geneontology.org/cgi-bin/amigo/go.cgi?query=GO%3A0048037&search_constraint=terms&action=query&view=query) | molecular_function | cofactor binding | 1 | 27 | 189 | 1.426 | 7.3e-4 |
| [GO:0050660](http://amigo.geneontology.org/cgi-bin/amigo/go.cgi?query=GO%3A0050660&search_constraint=terms&action=query&view=query) | molecular_function | flavin adenine dinucleotide binding | 2 | 16 | 66 | 2.189 | 8.2e-5 |
| [GO:0050662](http://amigo.geneontology.org/cgi-bin/amigo/go.cgi?query=GO%3A0050662&search_constraint=terms&action=query&view=query) | molecular_function | coenzyme binding | 1 | 20 | 125 | 1.590 | 2.3e-3 |
| [GO:0004497](http://amigo.geneontology.org/cgi-bin/amigo/go.cgi?query=GO%3A0004497&search_constraint=terms&action=query&view=query) | molecular_function | monooxygenase activity | 1 | 11 | 54 | 1.938 | 2.0e-2 |
| [GO:0009251](http://amigo.geneontology.org/cgi-bin/amigo/go.cgi?query=GO%3A0009251&search_constraint=terms&action=query&view=query) | biological_process | glucan catabolic process | 5 | 4 | 8 | 3.234 | 6.4e-2 |
| [GO:0030243](http://amigo.geneontology.org/cgi-bin/amigo/go.cgi?query=GO%3A0030243&search_constraint=terms&action=query&view=query) | biological_process | cellulose metabolic process | 5 | 4 | 6 | 3.649 | 2.0e-2 |
| [GO:0030245](http://amigo.geneontology.org/cgi-bin/amigo/go.cgi?query=GO%3A0030245&search_constraint=terms&action=query&view=query) | biological_process | cellulose catabolic process | 9 | 4 | 6 | 3.649 | 2.0e-2 |
| [GO:0044247](http://amigo.geneontology.org/cgi-bin/amigo/go.cgi?query=GO%3A0044247&search_constraint=terms&action=query&view=query) | biological_process | cellular polysaccharide catabolic process | 7 | 4 | 8 | 3.234 | 6.4e-2 |
| [GO:0051273](http://amigo.geneontology.org/cgi-bin/amigo/go.cgi?query=GO%3A0051273&search_constraint=terms&action=query&view=query) | biological_process | beta-glucan metabolic process | 5 | 4 | 6 | 3.649 | 2.0e-2 |
| [GO:0051275](http://amigo.geneontology.org/cgi-bin/amigo/go.cgi?query=GO%3A0051275&search_constraint=terms&action=query&view=query) | biological_process | beta-glucan catabolic process | 9 | 4 | 6 | 3.649 | 2.0e-2 |
| [GO:0008810](http://amigo.geneontology.org/cgi-bin/amigo/go.cgi?query=GO%3A0008810&search_constraint=terms&action=query&view=query) | molecular_function | cellulase activity | 1 | 4 | 6 | 3.649 | 2.0e-2 |
| [GO:0006801](http://amigo.geneontology.org/cgi-bin/amigo/go.cgi?query=GO%3A0006801&search_constraint=terms&action=query&view=query) | biological_process | superoxide metabolic process | 2 | 6 | 15 | 2.912 | 1.5e-2 |
| [GO:0072593](http://amigo.geneontology.org/cgi-bin/amigo/go.cgi?query=GO%3A0072593&search_constraint=terms&action=query&view=query) | biological_process | reactive oxygen species metabolic process | 2 | 6 | 19 | 2.571 | 4.8e-2 |
| [GO:0000302](http://amigo.geneontology.org/cgi-bin/amigo/go.cgi?query=GO%3A0000302&search_constraint=terms&action=query&view=query) | biological_process | response to reactive oxygen species | 2 | 6 | 19 | 2.571 | 4.8e-2 |
| [GO:0000303](http://amigo.geneontology.org/cgi-bin/amigo/go.cgi?query=GO%3A0000303&search_constraint=terms&action=query&view=query) | biological_process | response to superoxide | 3 | 6 | 10 | 3.497 | 9.9e-4 |
| [GO:0000305](http://amigo.geneontology.org/cgi-bin/amigo/go.cgi?query=GO%3A0000305&search_constraint=terms&action=query&view=query) | biological_process | response to oxygen radical | 3 | 6 | 10 | 3.497 | 9.9e-4 |
| [GO:0009636](http://amigo.geneontology.org/cgi-bin/amigo/go.cgi?query=GO%3A0009636&search_constraint=terms&action=query&view=query) | biological_process | response to toxic substance | 1 | 7 | 29 | 2.183 | 8.5e-2 |
| [GO:0019430](http://amigo.geneontology.org/cgi-bin/amigo/go.cgi?query=GO%3A0019430&search_constraint=terms&action=query&view=query) | biological_process | removal of superoxide radicals | 9 | 6 | 9 | 3.649 | 4.5e-4 |
| [GO:0034599](http://amigo.geneontology.org/cgi-bin/amigo/go.cgi?query=GO%3A0034599&search_constraint=terms&action=query&view=query) | biological_process | cellular response to oxidative stress | 4 | 7 | 21 | 2.649 | 1.4e-2 |
| [GO:0034614](http://amigo.geneontology.org/cgi-bin/amigo/go.cgi?query=GO%3A0034614&search_constraint=terms&action=query&view=query) | biological_process | cellular response to reactive oxygen species | 5 | 6 | 13 | 3.118 | 6.0e-3 |
| [GO:0071450](http://amigo.geneontology.org/cgi-bin/amigo/go.cgi?query=GO%3A0071450&search_constraint=terms&action=query&view=query) | biological_process | cellular response to oxygen radical | 6 | 6 | 9 | 3.649 | 4.5e-4 |
| [GO:0071451](http://amigo.geneontology.org/cgi-bin/amigo/go.cgi?query=GO%3A0071451&search_constraint=terms&action=query&view=query) | biological_process | cellular response to superoxide | 7 | 6 | 9 | 3.649 | 4.5e-4 |
| [GO:0098754](http://amigo.geneontology.org/cgi-bin/amigo/go.cgi?query=GO%3A0098754&search_constraint=terms&action=query&view=query) | biological_process | detoxification | 2 | 7 | 26 | 2.341 | 4.7e-2 |
| [GO:0098869](http://amigo.geneontology.org/cgi-bin/amigo/go.cgi?query=GO%3A0098869&search_constraint=terms&action=query&view=query) | biological_process | cellular oxidant detoxification | 2 | 7 | 26 | 2.341 | 4.7e-2 |
| [GO:1990748](http://amigo.geneontology.org/cgi-bin/amigo/go.cgi?query=GO%3A1990748&search_constraint=terms&action=query&view=query) | biological_process | cellular detoxification | 2 | 7 | 26 | 2.341 | 4.7e-2 |
| [GO:0004784](http://amigo.geneontology.org/cgi-bin/amigo/go.cgi?query=GO%3A0004784&search_constraint=terms&action=query&view=query) | molecular_function | superoxide dismutase activity | 2 | 6 | 10 | 3.497 | 9.9e-4 |
| [GO:0016209](http://amigo.geneontology.org/cgi-bin/amigo/go.cgi?query=GO%3A0016209&search_constraint=terms&action=query&view=query) | molecular_function | antioxidant activity | 1 | 7 | 27 | 2.286 | 5.7e-2 |
| [GO:0016721](http://amigo.geneontology.org/cgi-bin/amigo/go.cgi?query=GO%3A0016721&search_constraint=terms&action=query&view=query) | molecular_function | oxidoreductase activity, acting on superoxide radicals as acceptor | 1 | 6 | 10 | 3.497 | 9.9e-4 |
| [GO:0004112](http://amigo.geneontology.org/cgi-bin/amigo/go.cgi?query=GO%3A0004112&search_constraint=terms&action=query&view=query) | molecular_function | cyclic-nucleotide phosphodiesterase activity | 1 | 6 | 13 | 3.118 | 6.0e-3 |
| [GO:0004114](http://amigo.geneontology.org/cgi-bin/amigo/go.cgi?query=GO%3A0004114&search_constraint=terms&action=query&view=query) | molecular_function | 3',5'-cyclic-nucleotide phosphodiesterase activity | 1 | 6 | 13 | 3.118 | 6.0e-3 |
| [GO:0016829](http://amigo.geneontology.org/cgi-bin/amigo/go.cgi?query=GO%3A0016829&search_constraint=terms&action=query&view=query) | molecular_function | lyase activity | 1 | 17 | 130 | 1.299 | 7.2e-2 |
| [GO:0008652](http://amigo.geneontology.org/cgi-bin/amigo/go.cgi?query=GO%3A0008652&search_constraint=terms&action=query&view=query) | biological_process | cellular amino acid biosynthetic process | 7 | 9 | 39 | 2.118 | 2.7e-2 |
| [GO:0006144](http://amigo.geneontology.org/cgi-bin/amigo/go.cgi?query=GO%3A0006144&search_constraint=terms&action=query&view=query) | biological_process | purine nucleobase metabolic process | 8 | 5 | 13 | 2.855 | 5.3e-2 |
| [GO:0006542](http://amigo.geneontology.org/cgi-bin/amigo/go.cgi?query=GO%3A0006542&search_constraint=terms&action=query&view=query) | biological_process | glutamine biosynthetic process | 11 | 4 | 7 | 3.426 | 3.8e-2 |
| [GO:0004356](http://amigo.geneontology.org/cgi-bin/amigo/go.cgi?query=GO%3A0004356&search_constraint=terms&action=query&view=query) | molecular_function | glutamate-ammonia ligase activity | 1 | 4 | 7 | 3.426 | 3.8e-2 |
| [GO:0016211](http://amigo.geneontology.org/cgi-bin/amigo/go.cgi?query=GO%3A0016211&search_constraint=terms&action=query&view=query) | molecular_function | ammonia ligase activity | 1 | 4 | 7 | 3.426 | 3.8e-2 |
| [GO:0016880](http://amigo.geneontology.org/cgi-bin/amigo/go.cgi?query=GO%3A0016880&search_constraint=terms&action=query&view=query) | molecular_function | acid-ammonia (or amide) ligase activity | 1 | 4 | 7 | 3.426 | 3.8e-2 |
| [GO:0016614](http://amigo.geneontology.org/cgi-bin/amigo/go.cgi?query=GO%3A0016614&search_constraint=terms&action=query&view=query) | molecular_function | oxidoreductase activity, acting on CH-OH group of donors | 1 | 16 | 65 | 2.211 | 6.9e-5 |
| [GO:0004855](http://amigo.geneontology.org/cgi-bin/amigo/go.cgi?query=GO%3A0004855&search_constraint=terms&action=query&view=query) | molecular_function | xanthine oxidase activity | 1 | 4 | 6 | 3.649 | 2.0e-2 |
| [GO:0016727](http://amigo.geneontology.org/cgi-bin/amigo/go.cgi?query=GO%3A0016727&search_constraint=terms&action=query&view=query) | molecular_function | oxidoreductase activity, acting on CH or CH2 groups, oxygen as acceptor | 1 | 4 | 6 | 3.649 | 2.0e-2 |
| [GO:0004854](http://amigo.geneontology.org/cgi-bin/amigo/go.cgi?query=GO%3A0004854&search_constraint=terms&action=query&view=query) | molecular_function | xanthine dehydrogenase activity | 1 | 4 | 6 | 3.649 | 2.0e-2 |
| [GO:0016726](http://amigo.geneontology.org/cgi-bin/amigo/go.cgi?query=GO%3A0016726&search_constraint=terms&action=query&view=query) | molecular_function | oxidoreductase activity, acting on CH or CH2 groups, NAD or NADP as acceptor | 1 | 4 | 6 | 3.649 | 2.0e-2 |

**Table S11** Highly expressed digestive enzymes (TPM ratio > 0.75) in the trophosome of *Paraescarpia echinospica.*

| **Transcript ID** | **Digestive enzymes** | | **TPM ratio** |
| --- | --- | --- | --- |
|  | **Annotation** | **Description** |  |
| **Cathepsin** |  |  |  |
| Pec_DN67578C0G1I1 | Cathepsin L1 | proteolysis; cysteine-type peptidase activity | 1.0 |
| Pec_DN67578C1G1I1 | Cathepsin L1 | proteolysis; cysteine-type peptidase activity; hydrolase activity | 1.0 |
| Pec_DN16800C0G1I1 | Cathepsin Z | proteolysis; cysteine-type peptidase activity | 1.0 |
| Pec_DN67578C2G1I1 | Cathepsin L1 | proteolysis; cysteine-type peptidase activity; hydrolase activity | 1.0 |
| Pec_DN67578C0G2I4 | Cathepsin L1 | proteolysis; cysteine-type peptidase activity | 1.0 |
| Pec_DN55058C0G2I1 | Cathepsin B | peptidase activity; proteolysis; cysteine-type peptidase activity | 1.0 |
| Pec_DN70089C0G4I1 | Cathepsin L1 | proteolysis; cysteine-type peptidase activity; hydrolase activity | 0.9 |
| **Carboxypeptidase** |  |  |  |
| Pec_DN96311C0G1I1 | Carboxypeptidase B | carboxypeptidase activity; proteolysis | 1.0 |
| Pec_DN94224C0G1I1 | Carboxypeptidase B | carboxypeptidase activity; proteolysis | 0.8 |
| **PGRPs** |  |  |  |
| Pec_DN58800C0G1I3 | Peptidoglycan-recognition protein-SC2 precursor | innate immune response; N-acetylmuramoyl-L-alanine amidase activity | 0.9 |
| Pec_DN72034C0G1I1 | Peptidoglycan recognition 2 precursor | innate immune response; N-acetylmuramoyl-L-alanine amidase activity | 0.9 |
| **Others** |  |  |  |
| Pec_DN50858C0G3I1 | Elastase-like | breaks down elastin | 0.9 |
| Pec_DN68566C6G1I3 | Lysosomal thioesterase PPT2 | palmitoyl hydrolase activity; thiolester hydrolase activity | 0.9 |
| Pec_DN59302C1G3I1 | Lysosomal alpha-glucosidase | hydrolase activity; hydrolyzing O-glycosyl compounds | 0.8 |

**Table S12** Genes involved in bacterial adaptation, infection and intracellular survival in the *Paraescarpia echinospica* symbiont genomic analysis

|  | **Gene name** | | **Accession numer** | **Description** |
| --- | --- | --- | --- | --- |
| **Bacterial adaptation** | | | | |
| Bacterial chemotaxis | *fliGMNY* | | RLJ17223.1, RLJ21210.1, RLJ21211.1 | flagellar motor switch protein |
|  | *cheABDRVWYZ* | | RDH87638.1, RLJ21223.1, RLJ16723.1, RLJ19557.1, RLJ22187.1, RLJ16426.1, RLJ22699.1, RLJ18550.1, RDH87206.1 | chemotaxis family |
|  | *mcp*, *tsr*, *tap* | | RDH83298.1, RDH84277.1, RDH85165.1 | methyl-accepting chemotaxis proteins |
|  | *aer* | | RLJ18562.1 | aerotaxis receptor |
|  | *dppA* | | RLJ21767.1 | dipeptide transport system substrate-binding protein |
|  | *motAB* | | RLJ21224.1, RLJ21678.1 | chemotaxis proteins |
| **Bacterial infection** |  | |  |  |
| Fimbriae | *fimTU* | | RDH85510.1, RLJ17112.1 | type IV fimbrial biogenesis |
|  | *pilR* | | RLJ20304.1 | fimbriae expression regulatory |
| Flagellar | *flgABCDEFGHIKLMN*,  *fliACDEFGHIKMNYOZPQRS* | | RLJ16765.1, RLJ19325.1, RLJ19324.1, RLJ16760.1, RLJ16759.1, RLJ16758.1, RLJ16757.1, RLJ17033.1, RLJ19319.1, RLJ16754.1, RLJ16753.1, RLJ16766.1, RLJ16767.1; RDH87205.1, RLJ16748.1, RLJ21762.1, RLJ17225.1, RLJ17224.1, RLJ17223.1, RDH83478.1, RLJ17221.1, RLJ20094.1, RLJ21210.1, RLJ21211.1, RLJ21212.1, RDH87198.1, RDH87199.1, RLJ21242.1, RLJ19345.1 | flagellar biosynthesis protein |
|  | *flhAB* | | RDH87202.1, RLJ22708.1 | DNA-binding transcriptional dual regulator |
|  | *motAB* | | RLJ21224.1, RLJ21678.1 | motility protein |
| Pilus | *fimV* | | RDH87418.1 | type IV pilus assembly |
|  | *pilAGHIJLRS* | | RLJ18100.1, EGV50954.1, RDH81888.1, RDH81887.1, RDH81886.1, RDH84219.1, RLJ20304.1, RLJ16133.1 | twitching motility regulator |
|  | *chpAC* | | RDH81798.1, RLJ18586.1 | chemosensory pili system |
| Adhesin/invasin | *znuA* | | RDH83557.1 | ABC-type Zn^2+^ transport system, periplasmic component/surface adhesin |
|  | *inlA, yeeJ* | | RLJ22431.1; RLJ16638.1 | bacterial invasion of epithelial cells |
|  | *fliC* | | RLJ16748.1 | surface adhesion protein, flagellin |
| OmpR family | *phoBB1DPQR* | | RLJ21157.1, RDH84219.1, RLJ18322.1, RDH90511.1, RDH86697.1, RDH88274.1 | two-component system, OmpR family |
|  | *pagO* | | RLJ18613.1 | putative membrane protein PagO |
|  | *ompR* | | WP_057956068.1 | two-component system, OmpR family, phosphate regulon response regulator OmpR, biofilm formation |
|  | *envZ* | | RLJ19801.1 | two-component system, OmpR family, osmolarity sensor histidine |
| Some characterized proteins | *yqfA*, *tlyC* | | RDH87247.1, RDH85147.1, RLJ19751.1 | putative hemolysin III homolog |
|  | *kamA* | | OEU72331.1 | lysine 2,3-aminomutase |
|  | *cvpA* | | RDH88411.1 | the colicin V production protein |
| Biofilm formation | *pgaB* | | RLJ15846.1 | biofilm PGA synthesis lipoprotein |
|  | *pgaC* | | RDH89624.1 | biofilm PGA synthesis N-glycosyltransferase |
| OmpA-OmpF Porin (OOP) family | *ompA*, *ompF* | | RLJ22410.1, RDH87629.1 | outer membrane proteins of many Gram-negative bacteria |
| **Intracellular proliferation** | |  | | |
| DNA replication | *ligAB* | | RDH89410.1, RLJ17847.1 | DNA ligase (NAD+) |
|  | *recF* | | RLJ20626.1 | DNA replication and repair protein |
|  | *DPO1*, *polA* | | RDH91133.1, | DNA polymerase I |
|  | *POLR2* | | RLJ20577.1 | DNA polymerase II |
|  | *dnaBEGNQX*, *holABC* | | RLJ15958.1, RLJ21540.1, RLJ20627.1, RLJ16249.1, RDH89695.1, RDH89363.1, RLJ16646.1, RDH88271.1 | DNA polymerase III subunit |
|  | *ssb* | | RLJ18700.1 | single-strand DNA-binding protein |
|  | *rnhAB* | | RDH87524.1, RDH88755.1 | ribonuclease HI, ribonuclease HII |
| Cell cycle | *ftsAEIQWXYZ*, *CDH1*, *CDC14* | | RDH89940.1, RDH90333.1, RDH89947.1, RDH87908.1, RLJ22578.1, RDH82207.1, RDH82205.1, RLJ18517.1; RLJ17960.1; RDH84255.1 | cellular division involvement |

**Table S13** Genes involved in immune system process with different expression levels in the plume, vestimentum and trophosome as identified in the transcriptome analysis of *P. echinospica*. The transcript sequences were classified into seven groups according to their different roles in tubeworm immune system process, GO annotation and TPM values of each transcript were listed, the TPM values in three different regions represented the different expression levels of each transcript.

| **Sequence Name** | **Accession number** | **GO Annotation** | | **TPM (transcripts per million transcripts)** | | |
| --- | --- | --- | --- | --- | --- | --- |
| **Immune effector process** | | | **E-Value** | **Plume** | **Vestimentum** | **Trophosome** |
| Pec_DN69520C1G1I4 | ELU16283.1 | 2 -5 -oligoadenylate synthase 1A-like | 0.0 | 24.7 | 2.4 | 5.7 |
| Pec_DN6118C0G1I1 | XP_003942586.1 | S22902Ig kappa chain V region - human | 1.6E-73 | 0.0 | 0.0 | 4.7 |
| Pec_DN72831C1G2I2 | XP_019638355.1 | deoxynucleoside triphosphate triphosphohydrolase SAMHD1 isoform X2 | 1.5E-66 | 16.9 | 0.0 | 0.0 |
| Pec_DN35267C0G2I1 | XP_019624137.1 | neurotrophic tyrosine kinase receptor precursor | 1.4E-43 | 0.6 | 2.0 | 0.0 |
| **Activation of immune response** | | | | | | |
| Pec_DN73628C1G3I1 | XP_022287649.1 | toll-like receptor 6 | 9.2E-66 | 4.2 | 4.8 | 35.6 |
| Pec_DN9838C0G1I1 | XP_002435854.1 | N-acetylmuramoyl-L-alanine amidase | 1.3E-12 | 3.2 | 0.0 | 0.0 |
| **Immune system development** | | | | | | |
| Pec_DN60579C1G1I1 | ADC35037.1 | transcription factor GATA-3-like isoform X2 | 5.2E-133 | 235.5 | 1.9 | 19.1 |
| Pec_DN60732C0G2I3 | XP_004751552.1 | 60 kDa SS-A Ro ribonucleo | 8.7E-166 | 23.1 | 17.2 | 15.0 |
| Pec_DN65954C0G1I1 | XP_023929947.1 | NFX1-type zinc finger-containing 1-like | 0.0 | 1.3 | 1.4 | 8.3 |
| Pec_DN67744C0G2I2 | XP_023664605.1 | sperm flagellar 2 isoform X3 | 3.1E-12 | 1.1 | 0.7 | 7.6 |
| Pec_DN60946C0G1I1 | XP_022320796.1 | zinc finger 16-like isoform X2 | 1.0E-94 | 44.4 | 4.4 | 4.7 |
| Pec_DN58607C0G1I2 | XP_013387783.1 | inhibin beta A chain | 5.2E-20 | 6.0 | 0.3 | 0.0 |
| Pec_DN64064C0G3I1 | XP_021355273.1 | PR domain zinc finger 1 isoform X1 | 4.1E-143 | 58.4 | 0.2 | 0.0 |
| **Immune response** | | | | | | |
| Pec_DN44258C0G1I1 | ABW24373.1 | B cell translocation | 7.8E-88 | 259.7 | 480.9 | 191.6 |
| Pec_DN64702C0G1I1 | ELT94637.1 | serpin B9 isoform X1 | 3.7E-92 | 61.1 | 176.6 | 181.3 |
| Pec_DN64663C0G1I1 | ELU07807.1 | somatomedin-B and thrombospondin type-1 domain-containing -like | 3.5E-94 | 352.1 | 186.4 | 156.9 |
| Pec_DN73539C3G3I1 | XP_019617847.1 | interferon-induced GTP-binding mx | 7.8E-142 | 64.2 | 45.4 | 98.7 |
| Pec_DN72034C0G1I1 | AAY27974.1 | peptidoglycan recognition 2 precursor | 5.4E-58 | 2.1 | 11.3 | 87.4 |
| Pec_DN52517C0G1I1 | XP_014768681.1 | NF-kappa-B-repressing factor-like | 2.1E-63 | 149.3 | 102.0 | 68.0 |
| Pec_DN73892C0G4I1 | XP_004988329.1 | hypothetical protein PTSG_10280 | 1.8E-10 | 15.4 | 51.8 | 51.5 |
| Pec_DN58800C0G1I3 | ACV67267.1 | peptidoglycan-recognition -SC2 precursor | 5.7E-62 | 5.1 | 1.2 | 47.0 |
| Pec_DN56674C0G1I1 | XP_013399271.1 | NF-kappaB inhibitor | 9.7E-40 | 16.8 | 52.8 | 42.6 |
| Pec_DN70022C3G1I1 | ELT87410.1 | NF-kappa-B inhibitor-interacting Ras 1 | 2.7E-93 | 46.8 | 22.7 | 31.0 |
| Pec_DN63881C0G1I1 | XP_014767689.1 | ras-related Rab-14 | 5.0E-154 | 44.2 | 28.6 | 30.6 |
| Pec_DN60043C1G1I1 | ELU04547.1 | NF-kappaB p65 | 7.1E-127 | 23.6 | 13.6 | 29.9 |
| Pec_DN58696C0G1I1 | 5IKB_A | AChain Crystal Structure Of The Kainate Receptor Gluk4 Ligand Binding Domain In Complex With Kainate | 1.6E-38 | 190.4 | 4.3 | 29.7 |
| Pec_DN64881C0G1I1 | ACF93446.1 | NF-kappaB inhibitor | 2.4E-53 | 36.5 | 19.4 | 27.5 |
| Pec_DN58417C0G1I1 | ELU03643.1 | interleukin enhancer-binding factor 2-like | 0.0 | 70.7 | 25.3 | 19.3 |
| Pec_DN69533C2G1I1 | XP_022783794.1 | 60 kDa SS-A Ro ribonucleo | 6.5E-66 | 13.6 | 11.1 | 18.6 |
| Pec_DN73196C1G1I1 | XP_022315387.1 | Ectonucleotide pyrophosphatase phosphodiesterase family member 3 | 0.0 | 6.7 | 15.9 | 16.6 |
| Pec_DN64086C0G1I1 | XP_013406851.1 | tyrosine- kinase SRK2-like isoform X3 | 0.0 | 22.6 | 12.5 | 12.2 |
| Pec_DN70258C6G2I1 | ELT89677.1 | stimulator of interferon genes -like isoform X3 | 4.0E-57 | 15.8 | 7.6 | 10.8 |
| Pec_DN61282C0G1I4 | XP_020618305.1 | pantetheinase isoform X2 | 4.0E-127 | 0.1 | 2.4 | 5.6 |
| Pec_DN68490C2G1I1 | XP_002599109.1 | interleukin-1 receptor-associated kinase 1-binding 1-like | 5.4E-30 | 17.4 | 7.1 | 5.6 |
| Pec_DN73271C5G2I1 | XP_013403376.1 | tyrosine- kinase SRK2-like isoform X3 | 7.0E-29 | 1.7 | 3.3 | 5.6 |
| Pec_DN58122C0G1I2 | ELU16063.1 | poly(U)-specific endoribonuclease-C-like | 3.6E-78 | 37.4 | 10.3 | 4.9 |
| Pec_DN35964C0G1I1 | XP_013396924.1 | bactericidal permeability-increasing -like | 8.0E-57 | 1.0 | 0.8 | 4.8 |
| Pec_DN70783C1G2I2 | XP_019619643.1 | cytochrome b-245 heavy chain | 0.0 | 9.2 | 5.0 | 4.7 |
| Pec_DN598C0G1I1 | ELU10687.1 | AChain Solution Structure Of The Brca1 bard1 Ring-domain Heterodimer | 1.3E-30 | 0.5 | 0.6 | 4.1 |
| Pec_DN62581C0G1I1 | XP_009011054.1 | class B secretin-like G- coupled receptor | 5.7E-13 | 6.8 | 6.5 | 3.0 |
| Pec_DN62902C1G2I2 | XP_013382572.1 | DNA-directed RNA polymerase III subunit RPC6 | 7.0E-170 | 19.9 | 5.0 | 3.0 |
| Pec_DN29875C0G1I1 | XP_013413387.1 | NF-kappa-B-repressing factor | 1.1E-14 | 8.5 | 2.6 | 3.0 |
| Pec_DN71593C0G2I4 | XP_009024752.1 | caspase recruitment domain-containing 11 isoform X2 | 9.1E-12 | 5.6 | 4.9 | 2.7 |
| Pec_DN60759C0G1I2 | XP_019618428.1 | NF-kappa-B inhibitor 1 isoform X1 | 1.3E-70 | 9.9 | 4.6 | 2.4 |
| Pec_DN68058C1G1I12 | ELU18603.1 | toll-like receptor 2 | 3.0E-34 | 0.8 | 6.3 | 2.2 |
| Pec_DN72986C0G1I4 | XP_013382439.1 | NF-kappa-B inhibitor 2 | 0.0 | 6.5 | 1.9 | 2.0 |
| Pec_DN70173C1G4I1 | AAY27974.1 | peptidoglycan recognition 2 precursor | 2.0E-08 | 1.1 | 12.1 | 1.9 |
| Pec_DN62843C0G1I1 | XP_019643620.1 | AChain Trivalent Recognition Unit Of Innate Immunity System Crystal Structure Of Human M-Ficolin Fibrinogen-Like Domain | 1.6E-69 | 7.4 | 13.4 | 1.4 |
| Pec_DN71388C2G7I5 | XP_025096141.1 | B-cell linker -like | 4.4E-19 | 22.5 | 3.8 | 0.8 |
| Pec_DN58692C0G1I1 | ELU15484.1 | von Willebrand factor D and EGF domain-containing -like | 0.0 | 172.0 | 19.0 | 0.5 |
| Pec_DN54410C0G2I1 | ELT93940.1 | ANN1_AREMA ame: Full=Arenicin-1 Flags: Precursor | 3.4E-13 | 13.6 | 927.9 | 0.4 |
| Pec_DN72741C0G2I1 | ELT90079.1 | tubulointerstitial nephritis antigen | 4.6E-16 | 117.1 | 23.3 | 0.0 |
| Pec_DN58899C0G2I1 | XP_019647301.1 | sequestosome-1-like isoform X1 | 2.3E-43 | 2.1 | 0.3 | 0.0 |
| Pec_DN61724C0G1I2 | ELU02226.1 | tumor necrosis factor ligand superfamily member 14 isoform X3 | 1.6E-16 | 0.9 | 13.8 | 0.0 |
| Pec_DN61979C0G1I3 | XP_010971568.1 | proteoglycan 4 | 3.4E-44 | 0.0 | 45.0 | 0.0 |
| Pec_DN88647C0G1I1 | KGL88474.1 | tumor necrosis factor-inducible gene 6 | 3.8E-16 | 0.0 | 1.7 | 0.0 |
| Pec_DN67394C0G1I1 | ELU15564.1 | macrophage mannose receptor 1 | 3.1E-26 | 0.2 | 27.9 | 0.0 |
| Pec_DN69302C4G1I1 | XP_011448072.1 | peptidoglycan-recognition SC2-like isoform X4 | 3.2E-65 | 3.9 | 283.3 | 0.0 |
| Pec_DN51766C0G2I1 | AGS55438.1 | hairy enhancer of split 4 | 1.7E-48 | 8.5 | 12.6 | 0.0 |
| \| **Lectin group** \| \| --- \| | | | | | | |
| Pec_DN71465C0G1I5 | ELT94404.1 | 29-kDa galactose-binding lectin | 2.9E-65 | 121.4 | 91.1 | 132.4 |
| Pec_DN67563C0G1I1 | ELT97583.1 | chitinase-like lectin | 1.8E-119 | 73.7 | 28.2 | 51.7 |
| Pec_DN51517C0G4I1 | AWH12508.1 | C-type lectin 6 | 3.9E-13 | 67.0 | 64.8 | 25.0 |
| Pec_DN72088C2G1I2 | AWH12508.1 | C-type lectin domain family 4 member A-like | 2.2E-10 | 14.2 | 2.6 | 11.6 |
| Pec_DN68001C1G1I1 | SKA16809.1 | C-type lectin domain family 4 member M-like isoform X4 | 2.5E-13 | 4.5 | 8.7 | 77.6 |
| Pec_DN69154C0G1I1 | XP_013405149.1 | ERGIC-53-like isoform X2 | 2.5E-158 | 9.5 | 9.2 | 22.0 |
| Pec_DN25268C0G1I1 | XP_006818466.1 | F-type lectin | 1.8E-08 | 0.90 | 0.0 | 3.0 |
| Pec_DN34962C0G1I1 | ELU13756.1 | galactose-binding lectin | 1.3E-73 | 1.5 | 0.1 | 1.9 |
| Pec_DN71587C0G3I1 | AIG13081.1 | galectin-12 isoform X2 | 0.0 | 75.5 | 47.7 | 87.6 |
| Pec_DN70272C2G2I1 | ELU07244.1 | I-type lectin 3 | 4.2E-150 | 97.6 | 13.8 | 17.9 |
| Pec_DN63632C0G1I6 | XP_015768378.1 | rhamnose-binding lectin-like | 1.9E-13 | 39.1 | 16.4 | 16.4 |
| Pec_DN58640C0G1I1 | ELU14932.1 | Vesicular integral-membrane VIP36 | 2E-146 | 64.7 | 32.8 | 28.7 |
| **Antigen processing and presentation** | | | | | | |
| Pec_DN58601C0G1I1 | ELU01470.1 | CD9 antigen-like isoform X1 | 1.4E-55 | 3.1 | 0.7 | 136.6 |
| Pec_DN63631C0G1I1 | ELU16072.1 | 26S protease regulatory subunit 7 | 0.0 | 92.1 | 45.7 | 33.4 |
| Pec_DN73314C1G1I2 | ELT90449.1 | CD109 antigen-like isoform X3 | 1.6E-93 | 0.0 | 0.0 | 18.6 |
| Pec_DN66839C6G2I1 | XP_016392436.1 | squamous cell carcinoma antigen recognized by T-cells 3 | 1.2E-70 | 9.7 | 8.6 | 18.1 |
| Pec_DN73219C0G5I2 | ELU12898.1 | serologically defined colon cancer antigen 8 homolog | 8.9E-71 | 5.2 | 4.0 | 9.8 |
| Pec_DN63892C0G2I1 | ELU01489.1 | CD82 antigen-like | 4.7E-37 | 0.2 | 0.0 | 9.8 |
| Pec_DN64919C0G1I1 | SOV74292.1 | erythrocyte membrane-associated antigen | 1.1E-13 | 1.6 | 5.0 | 8.1 |
| Pec_DN49515C0G1I1 | PVD39429.1 | multidrug resistance 1 isoform X3 | 2.6E-162 | 0.0 | 0.0 | 4.1 |
| Pec_DN59035C0G1I2 | XP_013390853.1 | early endosome antigen 1-like isoform X2 | 1.7E-33 | 1.5 | 0.3 | 3.0 |
| **Immunoglobulin superfamily** | | | | | |  |
| Pec_DN73629C1G12I1 | PSN55937.1 | alpha-2-macroglobulin 1 isoform X1 | 4.8E-20 | 17.1 | 132.8 | 2991.6 |
| Pec_DN74029C5G5I1 | XP_013397771.1 | alpha macroglobulin | 1.8E-23 | 23.3 | 193.7 | 1588.6 |
| Pec_DN73629C1G2I1 | XP_013397771.1 | C3 and PZP-like alpha-2-macroglobulin domain-containing 8 | 1.4E-15 | 0.5 | 8.6 | 1453.8 |
| Pec_DN74029C5G3I1 | AVP12670.1 | PREDICTED: alpha-2-macroglobulin-like | 8.8E-67 | 26.2 | 146.8 | 1304.6 |
| Pec_DN69421C1G4I1 | XP_013397771.1 | alpha-2-macroglobulin 2 | 3.5E-07 | 6.1 | 17.4 | 434.4 |
| Pec_DN71153C3G3I1 | AVP12670.1 | alpha-2-macroglobulin precursor splice variant 1 | 6.4E-14 | 0.7 | 4.6 | 410.8 |
| Pec_DN67858C0G1I1 | XP_021352803.1 | alpha-2-macroglobulin receptor-associated -like | 1.9E-43 | 37.3 | 42.1 | 54.9 |
| Pec_DN68093C3G1I1 | XP_025106837.1 | leucine-rich repeats and immunoglobulin-like domains 2 | 0.0 | 33.8 | 19.6 | 15.5 |
| Pec_DN67924C3G1I3 | ELT88114.1 | leucine-rich repeat and immunoglobulin-like domain-containing nogo receptor-interacting 4 | 8.1E-102 | 76.8 | 14.0 | 13.8 |
| Pec_DN59559C1G2I1 | ELT88274.1 | immunoglobulin-binding 1-like | 3.7E-88 | 19.5 | 11.7 | 10.7 |
| Pec_DN74157C5G3I1 | XP_019929631.1 | immunoglobulin superfamily DCC subclass member 4 | 4.5E-14 | 26.3 | 40.2 | 9.4 |
| Pec_DN59122C1G1I1 | ELU10778.1 | leucine-rich immunoglobulin-like domain and transmembrane domain-containing 3 isoform X3 | 2.9E-26 | 68.8 | 970.2 | 7.1 |
| Pec_DN72064C1G1I1 | ELU15044.1 | leucine-rich repeats and immunoglobulin-like domains 3 | 1.2E-105 | 7.1 | 4.4 | 6.2 |
| Pec_DN65459C0G4I1 | XP_022294895.1 | C3 and PZP-like alpha-2-macroglobulin domain-containing | 2.5E-16 | 20.8 | 20.0 | 1.3 |
| Pec_DN53841C0G2I1 | ELU07850.1 | leucine-rich repeat and immunoglobulin-like domain-containing nogo receptor-interacting 3 isoform X2 | 2.2E-67 | 9.2 | 14.4 | 1.3 |
| Pec_DN70877C2G2I1 | ELT90637.1 | leucine-rich repeat and immunoglobulin-like domain-containing nogo receptor-interacting 1 | 7.7E-46 | 14.3 | 13.0 | 0.4 |

Abbreviation list

1,3-BPG 1,3-bisphosphoglycerate

2PG 2-phosphoglycerate

3PG 3-P-Glycerate

67LR 40S ribosomal protein SA (P40)/67kD laminin receptor precursor

AAA MoxR AAA+ ATPases

ACLY ATP citratelyase

AcnA Aconitase A

ACON/ACN Aconitase

ACTG1 Actin, cytoplasmic 2

ACTP Actin-related proteins

ADK adenylate kinase

AdoHcyase S-adenosylhomocysteine hydrolase

AFP Actin filament-coating protein tropomyosin

ALDO Fructose-biphosphate aldolase

AlgG mannuronan 5-epimerase

AMPH Amphiphysin

ANKR Ankyrin repeat

APJUN Transcriptional activator of the JUN family

Apr Adenylyl-sulfate reductase

APS Adenylyl sulfate

AS Anthranilate/para-aminobenzoate synthases component I

ASK Aspartokinase

Asr Anaerobic sulfite reductase (asrABC)

ATP7 ATP synthase subunit mitochondrial-like

ATPF1A ATP synthase subunit alpha

ATP-PFK 6-phosphofructokinase

ATP-synt F0F1-type ATP synthase, alpha subunit

BamC outer membrane protein assembly factor BamC

BarA Hybrid sensor histidine kinase/response regulator

BDS1 Alkyl sulfatase BDS1 and related hydrolases, metallo-beta-lactamase superfamily

CA carbonic anhydrase

CAP1 Adenylyl cyclase-associated 1

CAPTEDRAFT_198366 hypothetical protein CAPTEDRAFT_198366

CAPTEDRAFT_199465 hypothetical protein CAPTEDRAFT_199465, partial

Cbb3-Cox subunit I Cytochrome c cbb3-type subunit I

CbiA Cobyrinate a,c-diamide synthase

CCP Cytochrome c peroxidase

CEP52 Ubiquitin-60S ribosomal protein L40

CHI chitinase

CNN Calponin

COL4A4 collagen alpha-4(VI) chain-like

COX5A cytochrome c oxidase subunit Va

Cox6c2 Cytochrome c oxidase subunit 6C-2

CPSase carbamoyl-phosphate synthase small subunit

CpSecY Preprotein translocase subunit SecY

CS Citrate synthase

CTF1 Beta-1,6-N-acetylglucosaminyltransferase, contains WSC domain

CTSD cathepsin D

CTSL cathepsin L1-like

CTSL1 cathepsin L1

CYCS Cytochrome c

CYTC Cytochrome c553

DapB 4-hydroxy-tetrahydrodipicolinate reductase

DDAH1 N(G), N(G)-dimethylarginine dimethylaminohydrolase 1-like isoform X1

DDO D-aspartate oxidase-like

DHAP Dihydroxyacetone phosphate

DHX9 ATP-dependent RNA helicase A

DsbA thiol: disulfide interchange protein DsbA

DsbC thiol:disulfide interchange protein DsbC

Dsr Dissimilatory sulfite reductase (dsrAB)

DsrC Dissimilatory sulfite reductase (Desulfoviridin), gamma subunit

DTD D-Tyr-tRNAtyr deacylase

E4P Erythrose-4-P

ECHS1 enoyl-CoA hydratase

EF-2 Elongation factor 2

EmrA RND family efflux MFP subunit

ENO Phosphopyruvate hydratase (Enolase)

EnvZ osmolarity sensor histidine kinase

ETHE1 Per sulfide dioxygenase

F1,6BP Fructose 1,6-bisphosphate

F6P Fructose-6-P

FabB 3-oxoacyl-[acyl-carrier-protein] synthase

FBA Fructose-bisphosphate aldolase

FBP Fructose-bisphosphatase

FccA Cytochrome subunit of sulfide dehydrogenase

FccB Sulfide dehydrogenase flavoprotein chain

FDH Formate dehydrogenase (cytochrome-c-553)

FeS Fe-S-cluster oxidoreductase

FH Fumarate hydratase

FLNA filamin-A-like isoform X8

FP2_01370 Membrane protease subunits, stomatin/prohibitin homologs

FR Fumarate reductase

FstH Integral membrane protein, interacts with FtsH

FUM Fumarase

G3P Glyceraldehyde-3-P

G6P Glucose 6-phosphate

GADP Glyceraldehyde 3-phosphate

GADPH Glyceraldehyde-3-phosphate dehydrogenase

GBL 29-kDa galactose-binding lectin

GGT Gamma-glutamyltransferase

Gld glucose dehydrogenase [FAD, quinone]-like isoform X6

GlnA Glutamine synthetase

GlnB Nitrogen regulatory protein P-II

GLT Glutamate synthase

GNB2L1 Guanine nucleotide-binding protein subunit beta-2-like 1

GNB2L1 guanine nucleotide-binding subunit beta-2-like 1

Group_II_RT_mat Group II intron reverse transcriptase/maturase

H+, Na+-PPase Hydrogen, sodium-translocating pyrophosphatase

H4L histone H4-like

HBA1 Hemoglobin subunit A1

HBA2 Hemoglobin subunit A2

HBB1 Hemoglobin subunit B1

HBB2 hemoglobin subunit B2

HBV1A1 A1 globin chain of V1 giant hemoglobin

HBV1L1 L1 linker of V1 giant hemoglobin

HBV1L4 L4 linker of V1 giant hemoglobin

HBV2B1 B1 globin chain of giant V2 hemoglobin

HdrA Heterodisulfide reductase, subunit A and related polyferredoxins

HdrB Heterodisulfide reductase, subunit B

HflK membrane protease subunit HflK

HIF1AN hypoxia-inducible factor 1-alpha inhibitor

HINT1 histidine triad nucleotide-binding 1

HK Hexokinase

HNRNPA3 heterogeneous nuclear ribonucleo A3-like

HPDL 4-hydroxyphenylpyruvate dioxygenase-like

HSP Molecular chaperone

HSP10 10 kDa heat shock mitochondrial-like

HSP20 Small heat shock protein HSP20

HSP60 Chaperonin GroEL (HSP60 family)

HSP60 60 kDa heat shock mitochondrial-like

HSP70 Molecular chaperones GRP78/BiP/KAR2, HSP70 superfamily

HSP70 heat shock -70kDa

HyuB N-methylhydantoinase B/acetone carboxylase, alpha subunit

IDH Isocitrate dehydrogenase

I-FABP fatty acid-binding intestinal-like

ILVC ketol-acid reductoisomerase

IS1595 IS1595 family

JmjC DNA-binding protein jumonji/RBP2/SMCY, contains JmjC domain

KGOR 2-oxoglutarate: ferredoxin oxidoreductase (korAB)

KorB 2-oxoglutarate oxidoreductase

L1CAM Neural cell adhesion molecule L1

LaminIF Putative nuclear envelope protein lamin intermediate filament superfamily

LMNB1 lamin-B1-like isoform X2

Lyase_II Polysaccharide lyase family domain II

M48 peptidase M48

MCHLO_11918 RhoA GTPase effector DIA/Diaphanous

MDH Malate dehydrogenase

MDR Multidrug resistance efflux pump

Melm myosin essential light striated adductor muscle-like

MhmA Myosin heavy striated muscle-like isoform X4

MhmA myosin heavy striated muscle-like isoform X2

MHr Myohemerythrin

MHr HEMTM_RIFPA ame: Full=Myohemerythrin Short=MHr

Mla ABC-type transporter Mla maintaining outer membrane lipid periplasmic component

MPST 3-mercaptopyruvate sulfurtransferase

MRI1 methylthioribose-1-phosphate isomerase

MrlC Myosin regulatory light chain

MRM rRNA methyltransferase mitochondrial-like

Mrp Chromosome partitioning Mrp family

MS4A4A Membrane-spanning 4-domains subfamily A member 4A isoform X1

MyoL PREDICTED: myophilin-like

MyoL myophilin-like isoform X1

N2OR Nitrous-oxide reductase

NACTSN Transcription factor containing NAC and TS-N domains

NadB L-aspartate oxidase

Nap Periplasmic nitrate reductase (napAB)

Nar Respiratory nitrate reductase

NCX sodium-calcium exchanger

NirBD Nitrite reductase (NADH)

NirKS Nitrite reductase (NO-forming)

NIT Nitrilase

NIT2 Nitrilase member 2

Nor Nitric-oxide reductase

NR Nitrite reductase

NrfAH Nitrite reductase (cytochrome; ammonia-forming)

NuoG NADH-quinone oxidoreductase subunit G

OAA Oxaloacetate

oadA Pyruvate/oxaloacetate carboxyltransferase

Orf91 ISxac3 transposase

PAPS 3’-phosphoadenylyl sulfate

PD Pyruvate dehydrogenase

PDIA3 disulfide-isomerase A3-like

PDLIM7 PDZ and LIM domain 7-like

PdxS phosphate synthase pdxS subunit

PEP Phosphoenolpyruvate

PEPC Phosphoenolpyruvate carboxylase

PEPCK Phosphoenolpyruvate carboxykinase [GTP]

PFK Phosphofructokinase-1

PFOR Pyruvate: ferredoxin oxidoreductase

PGI Glucose-6-phosphate isomerase

PGK Phosphoglycerate kinase

PGM Phosphoglycerate mutase

PHD procollagen-proline dioxygenase beta subunit

PHGDH D-3-phosphoglycerate dehydrogenase

Phs Thiosulfate reductase (quinone)

PHYHD1 phytanoyl- dioxygenase domain-containing 1-like

PilQ pilus assembly

PK Pyruvate kinase

PKD Polycystic kidney disease (PKD) domain

PLC Phospholipase C

PMLI Paramyosin-like isoform X3

PMPCA mitochondrial-processing peptidase subunit alpha

PNP polyribonucleotide nucleotidyltransferase

PPIB Peptidyl-prolyl cis-trans isomerase

PPIL peptidyl-prolyl cis-trans isomerase-like

PPIs Parvulin-like peptidyl-prolyl isomerase

PPK Polyphosphate kinase

PPS Phosphoenolpyruvate synthase

PRDX6 peroxiredoxin 6

PRK Phosphoribulokinase

PSE Putative secreted esterase

PSMA4 proteasome subunit alpha type-4

PSMA6 proteasome subunit alpha type-6-like

PSP Predicted periplasmic/secreted protein

PstS ABC-type phosphate transport system, periplasmic component

PurH Phosphoribosylaminoimidazolecarboxamide formyltransferase/IMP cyclohydrolase

R5P Ribose-5-P

RAB7A ras-related Rab-7a

Rho transcription termination factor Rho

RISP Ubiquinol-cytochrome c reductase iron-sulfur subunit

RPE Ribose-phosphate epimerase

Rpi Ribose-phosphate isomerase

RPL30 60S ribosomal L30

RPL4 60S ribosomal protein L4

RplB 50S ribosomal protein L2

RplS 50S ribosomal protein L19

RplY Ribosomal protein L25 (general stress protein Ctc)

RpoD DNA-directed RNA polymerase sigma subunit (Sigma70/sigma32)

RpS27A Ubiquitin-40S ribosomal protein S27a

RpS30 Ubiquitin-like/40S ribosomal S30 protein fusion

RPS7 30S ribosomal S7

RPS8 40S ribosomal protein S8

rrtT Retron-type reverse transcriptase

RTO_06290 NADPH-dependent glutamate synthase beta chain and related oxidoreductases

Ru5P Ribulose-5-P

RuBisCO Ribulose-1,5-bisphosphate carboxylase/oxygenase

RuBP Ribulose-1,5-bis-P

S-1,7-BP Sedoheptulose-1,7-bis-P

S7P Sedoheptulose-7-P

SBA Sedoheptulose-bisphosphate aldolase

SBP Sedoheptulose-bisphosphatase

SCP Sarcoplasmic calcium-binding protein

SCS Succinyl coenzyme A synthetase (sucCD)

SDH Succinate dehydrogenase

SDHA Succinate dehydrogenase or fumarate flavo subunit

SELENBP1 selenium-binding 1

Sgp Sulfur globule protein

SHLC Myosin, essential light chain, adductor muscle

SM20 20 kDa calcium-binding (Antigen SM20)

SOD1 Cu2+/Zn2+ superoxide dismutase

SORD Sorbitol dehydrogenase

Sox Sulfur oxidation

Spase signal peptidase I

SPINT Kunitz-type protease inhibitor

Sqr Sulfide: quinone reductase

SseA Rhodanese-related sulfurtransferase

Staufen Double-stranded RNA-binding Staufen homolog 2-like isoform X1

SucC Succinyl-CoA synthetase, beta subunit

Succ_Dhase_FrdA_Gneg Succinate dehydrogenase/fumarate reductase, flavoprotein subunit

SucD Succinyl-CoA synthetase, alpha subunit

TC.GBP Outer membrane protein (porin)

TC.OOP OmpA-OmpF porin, OOP family

TCTP Microtubule-binding protein (translationally controlled tumor protein)

TEF Transcription elongation factor GreA

TEF1 translation elongation factor EF-1 alpha

TF_bZIP Transcriptional activator FOSB/c-Fos and related bZIP transcription factors

TKT Transketolase

Tnni Troponin I

TnT Troponin T

TPI Triose-phosphate isomerase

TPI triosephosphate isomerase B-like

Tpm TPM_HALRU ame: Full=Tropomyosin

TpxA thioredoxin peroxidase

Transgelin Transgelin-3-like isoform X3

TrpB tryptophan synthase subunit beta

TRX Thioredoxin 1

TrxB thioredoxin reductase

Tscrpt_reg_Rrf2 Rrf2 family transcriptional regulator

TST Thiosulfate sulfurtransferase

Ttr Tetrathionate reductase

TUBA Tubulin alpha chain

TUBB tubulin beta chain isoform X1

USP universal stress A isoform X2

VDAC2 voltage-dependent anion channel protein 2

VprBP HIV-1 Vpr-binding protein

WbpV NAD-dependent epimerase/dehydratase

X5P Xylulose-5-P

YWHAZ 14-3-3 zeta

α-KGDH α-ketoglutarate dehydrogenase
